# Expanding the Chemoproteomic Toolkit to Asparagine and Glutamine

**DOI:** 10.1021/acschembio.6c00173

**Published:** 2026-04-09

**Authors:** Benjamin Emenike, John M. Talbott, Zachary E. Paikin, Christian M. Beusch, Sohail Khoshnevis, David E. Gordon, Monika Raj

**Affiliations:** † Department of Chemistry, 1371 Emory University, Atlanta, Georgia 30322, United States; ‡ Department of Pathology and Laboratory Medicine, Emory University, Atlanta, Georgia 30322, United States; § Department of Surgical Sciences, Uppsala University, Uppsala 751 85, Sweden; ∥ Department of Biochemistry, 12239 Emory University School of Medicine, Atlanta, Georgia 30322, United States

## Abstract

Chemoproteomic strategies have revolutionized proteome annotation by targeting nucleophilic and redox-active side chains. However, the primary amides of asparagine (Asn) and glutamine (Gln) have long lacked robust chemical tools for proteome-wide interrogation. We report a chemoselective palladium-mediated dehydration that converts Asn/Gln amides to nitriles under mild aqueous conditions. This transformation enables the first proteome-wide mapping of chemically addressable Asn/Gln sites in lysates and living cells. Leveraging this reactivity, we establish an inverse chemoproteomic framework in which reduced nitrile formation reports PTM-mediated protection of Asn/Gln sites, including those impacted by deamidation and N-glycosylation. This approach reveals sites masked by post-translational modifications (PTMs), specifically those associated with deamidation and N-glycosylation. In yeast, this framework expanded the known N-glycoproteome, identifying numerous candidates missed by traditional glycopeptide enrichment due to low abundance or noncanonical motifs. Furthermore, comparative profiling in *Candida albicans* captured the dynamic remodeling of glycosylation patterns during morphogenesis. This dehydration-to-nitrile platform establishes a scalable handle on the amide proteome to map residue accessibility and PTM-linked site dynamics across biological states.

## Graphical abstract

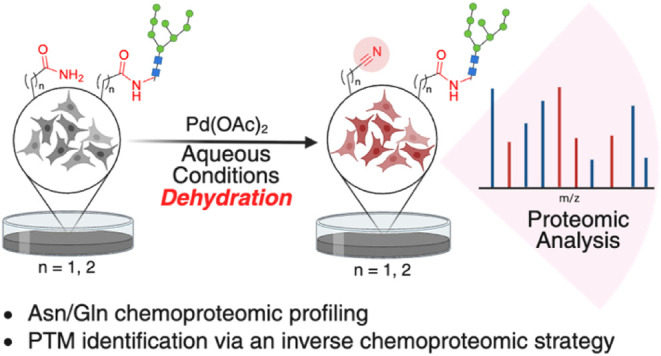

## Introduction

The human proteome is chemically diverse, yet nearly 9% of its residue side chains are the primary amides of asparagine (Asn) and glutamine (Gln), which remain under-profiled because they are difficult to access by global chemical profiling.^1^ While chemoproteomic platforms have successfully mapped the reactivity and ligandability of nucleophilic and redox-sensitive residues,^2–8^ the inherent chemical inertness of Asn and Gln amides has kept them largely out of reach. This technological gap has constrained proteome-wide efforts to connect Asn/Gln chemistry to protein structure and regulation.^9^

Herein, we introduce a platform for global Asn/Gln profiling via a palladium-mediated dehydration reaction that converts primary amides to nitrile products under mild, aqueous conditions. This transformation proceeds through the net loss of water (−H_2_O) to generate a compact nitrile handle that is stable and readily detected by LC-MS. By enabling proteome-scale conversion of Asn/Gln amides to a common nitrile end point, this chemistry supports global profiling in both lysates and live cells enabling mapping of these residues in complex biological systems (Figure 1A). The ability to selectively modify Asn and Gln amides enables a powerful inverse chemoproteomic strategy for interrogating post-translational modifications (PTMs). In this framework, PTMs that mask or alter the amide side chain, such as deamidation or N-glycosylation, inhibit conversion to the nitrile product, yielding a protection signature that can be used to identify PTM-associated candidates. This protection-based framework offers a practical advantage for studying deamidation-associated changes by converting available amides to nitriles early in the workflow, thereby reducing the opportunity for artifactual, sample-preparation-associated deamidation to obscure biological patterns.^10,11^ In addition, the approach provides an orthogonal perspective to enzyme- and enrichment-based methods by reporting on chemically addressable amide sites without relying on enzymatic substrate preferences, metabolic tag incorporation, or enrichment biases.^12–22^ In this work, we apply a palladium-mediated dehydration strategy to prioritize deamidation-associated candidates and to profile remodeling of glycosylation-associated protection patterns in *Saccharomyces cerevisiae* and *Candida albicans*, demonstrating the utility of this chemical strategy for comparative analysis of PTM-associated landscapes across biological states linked to stress responses and fungal virulence.

**Figure 1:**
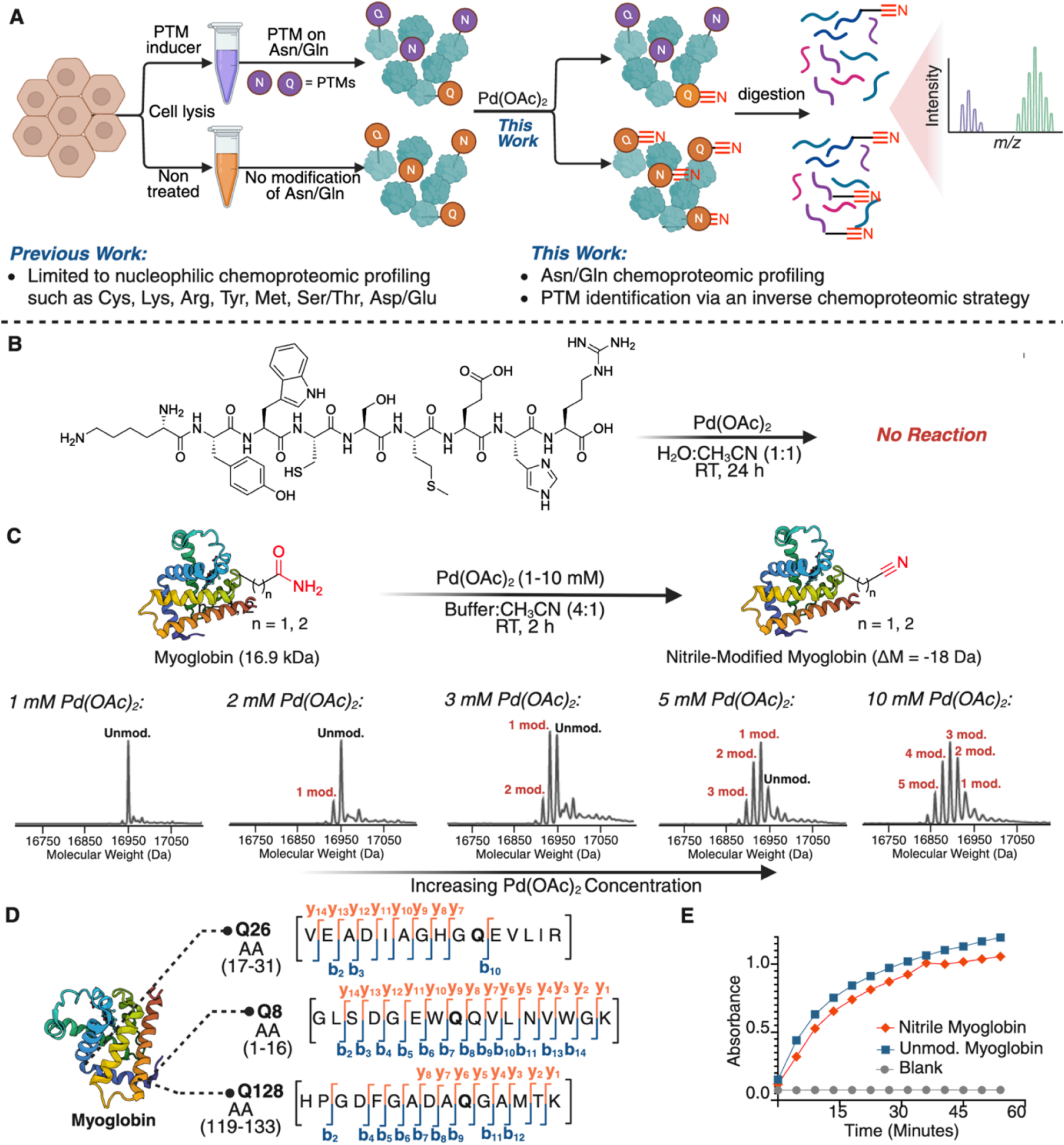
Development of the Asn/Gln dehydration reaction on proteins. A) Previous work established chemoproteomic strategies targeting nucleophilic and redox-sensitive amino acid side chains, including cysteine, lysine, arginine, tyrosine, methionine, serine/threonine, and aspartic/glutamic acid. The present work expands the chemoproteomic toolbox to chemically inert primary amide side chains of asparagine and glutamine through palladium-mediated dehydration chemistry, enabling profiling of deamidation and N-glycosylation PTMs through an inverse chemoproteomics approach. B) Chemoselectivity: peptide KYWSMEHR (S3) does not undergo any modification under the reaction conditions. C) Optimization of the dehydration reaction on myoglobin as a function of Pd­(OAc)_2_ concentration (1–10 mM), monitored by intact protein mass spectrometry, revealing dose-dependent nitrile formation and heterogeneous modification at elevated palladium concentrations. Homogeneous single modification was observed using a lower concentration of Pd­(OAc)_2_ (3 mM). (D) Optimization of the dehydration reaction of myoglobin using a lower concentration of 3 mM of Pd­(OAc)_2_ for 2 h yielded a homogeneously modified nitrile protein. MS/MS analysis identified Gln8, Gln26, and Gln128 as the sites of modification. Observed y and b ions are labeled. (E) Homogeneously modified myoglobin demonstrates a similar ability to oxidize *o*-phenylenediamine compared to unmodified myoglobin. These data support the hypothesis that the 3D structure of the myoglobin remained intact after the modification under the reaction conditions. Created in BioRender. Lab, R. (2026) https://BioRender.com/978dmxn.

## Results

### Development of Dehydration Chemistry for Protein Modification

To establish palladium-mediated dehydration as a chemoproteomic platform for targeting primary amide side chains of Asn/Gln,^23–26^ we first evaluated reaction performance under mild, aqueous conditions using small-molecule and peptide benchmarks. A model substrate, 2-phenylacetamide, underwent efficient conversion to 2-phenylacetonitrile (98% yield) using 10 mol % Pd­(OAc)_2_ in 1:1 H_2_O:CH_3_CN at RT, consistent with productive dehydration to the nitrile product (Supplementary Figure S1). Based on our previously established conditions for peptide nitrile formation utilizing 3 equiv. Pd­(OAc)_2_ in 1:1 H_2_O:CH_3_CN at RT for 2 h,^27^ peptide WRFNGLRG (S1) was quantitatively converted to the corresponding nitrile peptide WRFN­(CN)­GRLG (S2) (Supplementary Figure S2a-d). MS/MS analysis confirmed site-specific conversion at the Asn residue (Supplementary Figure S2e). We then evaluated chemoselectivity on a multifunctional model peptide (KYWSMEHR, S3) that contains reactive side chains commonly targeted by residue-centric probes (Lys, Tyr, Trp, Ser, Met, His, and Arg) (Figure 1B, Supplementary Figure S2e-g). Under dehydration conditions, using Pd­(OAc)_2_, we observed no detectable modification of any of these residues, as analyzed by LC-MS, supporting preferential reactivity toward primary amides and no cross-reactivity with any reactive functional groups exploited by established chemoproteomic tools (Figure 1B, Supplementary Figure S2).

We then optimized the reaction on a model protein, myoglobin, to evaluate its performance in the context of folded protein architectures. Titration of Pd­(OAc)_2_ revealed a clear concentration dependence in the extent and heterogeneity of modification (Figure 1C, Supplementary Figure S3). At higher palladium concentrations (10 mM), labeling became heterogeneous (up to 5–6 modifications), whereas 3 mM Pd­(OAc)_2_ produced predominantly one to two nitrile conversions per protein molecule, providing a practical operating window for controlled modification.

High-resolution mass spectrometry confirmed mass shifts corresponding to dehydration in all screened concentration conditions, and MS/MS analysis identified Gln8, Gln26, and Gln128 as the sites of modification in the homogeneous (3 mM Pd­(OAc)_2_) sample (Figure 1D, Supplementary Figure S3).

A key feature of this transformation is its compact chemical outcome: dehydration converts the amide to a nitrile through a net loss of water (−H_2_O), yielding a small product that avoids bulky appendages. To probe whether nitrile formation is compatible with retention of protein function in this model system, we evaluated the peroxidase-like activity of nitrile-modified myoglobin. The modified protein retained activity comparable to the native protein, consistent with preservation of the heme environment and overall fold under the optimized conditions (Figure 1E, Supplementary Figure S4). Together, these results establish palladium-mediated amide dehydration as a chemoselective, protein-compatible route to access Asn/Gln-derived nitriles and motivate its extension to proteome-scale profiling.

### Asn/Gln Chemoproteomic Profiling

We next applied palladium-mediated amide dehydration to proteome-scale profiling of Asn/Gln sites in cell lysates, leveraging nitrile formation as a direct LC-MS readout of chemically addressable primary amides in complex mixtures.

To define an experimental reactivity landscape, we treated T-47D breast cancer cell lysates with increasing concentrations of Pd­(OAc)_2_ (100 μM to 1 mM), followed by quenching of palladium, trypsin digestion, and LC-MS/MS analysis. Across the proteome, we observed clear dose-dependent conversion of Asn/Gln to nitriles and identified 2,544 unique proteins at 1 mM Pd­(OAc)_2_ (Figure 2A, Supplementary Figure S5). An analogous control sample prepared without Pd­(OAc)_2_ yielded only two unique proteins containing a confidently localized Gln (−18.0106 Da) modification, indicating a very low background level in the absence of palladium (Supplementary Figure S5a). In addition, open-search ΔMass analysis identified −18.0106 Da as the dominant treatment-dependent mass shift across Pd concentrations, supporting assignment of this signal to the palladium-mediated dehydration chemistry rather than to a search artifact (Supplementary Figure S5b). Furthermore, we observed a dose-dependent increase in the percentage of peptides bearing a – 18.0106 Da modification on Asn/Gln, whereas the percentage of peptides assigned this mass shift on other potentially dehydrating residues, including Ser, Thr, Asp, and Glu, remained largely unchanged (Supplementary Figure S5a). To focus on the most consistently observed sites, we prioritized high-occupancy conversion events detected across all dosing conditions, yielding 445 recurrent Asn/Gln conversion sites across 256 proteins spanning enzymes, regulatory factors, and structural proteins (Figure 2A, Supplementary Figure S5). Peptides were identified with high-confidence b and y ions (Supplementary Figure S5c). Sequence-context analysis of these recurrent sites revealed enrichment of nearby acidic (E, D) and polar (T) residues, consistent with local electrostatic and hydrogen-bonding environments that may modulate amide conversion efficiency (Figure 2A). We then extended the workflow to intact-cell labeling to assess compatibility with live-cell proteomics. Exposure of T-47D cells to varying concentrations of Pd­(OAc)_2_ (ranging from 10 μM to 100 μM) and 2% acetonitrile for 2 h revealed 92–96% cell viability at different concentrations (Supplementary Figure S6). Subsequent cellular lysis, digestion, and LC–MS/MS analysis of treated cells revealed dose-dependent Asn/Gln conversion and identified 204 recurrent nitrile-bearing peptides mapping to 141 proteins in the intact-cell context (Figure 2B, Supplementary Figure S7). Peptide identification was confirmed through high-confidence b and y ion spectra (Supplementary Figure S7). Comparison of lysate and live-cell data sets identified 66 overlapping proteins, with additional proteins observed uniquely in the live-cell condition (Figure 2B). Notably, the live-cell data set was enriched for membrane-associated proteins (including anchoring junctions and focal adhesion proteins), yielding a profile distinct from the lysate data set, which contained a larger fraction of soluble/cytosolic proteins (Figure 2B).

**Figure 2:**
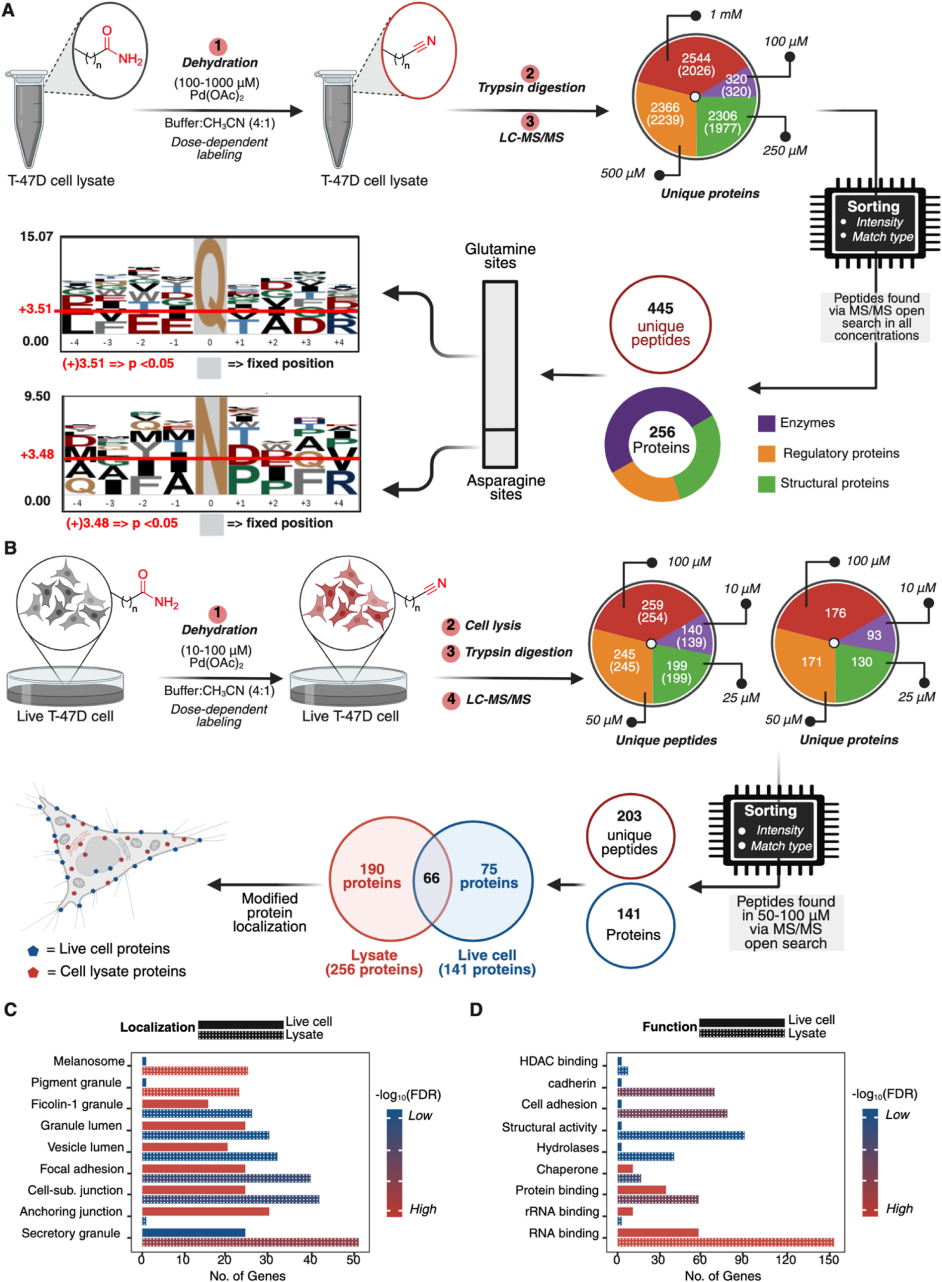
Palladium-mediated dehydration for chemoproteomic profiling of asparagine and glutamine in lysates and in live cells. (A) Reactive asparagine and glutamine profiling with a dehydration reaction through treatment of T-47D breast cancer cell lysate with low (100 μM), medium (250 μM), high (500 μM), and superhigh doses (1 mM) of Pd­(OAc)_2_ in a 4:1 NaP buffer (10 mM, pH 7.2):CH_3_CN, followed by palladium quenching, trypsin digestion, and LC-MS/MS analysis. The analysis of results identified the modification of 320 unique proteins at 100 μM, 2306 at 250 μM, 2366 at 500 μM, and 2544 at 1 mM; values in parentheses represent the number of proteins identified through MS/MS peptides alone, while the total number represents MS/MS and MBR peptides. The identification of hyperreactive Asn/Gln sites and sequence motif analysis through sorting and retaining peptides with nonzero intensity and a match type of “MS/MS” across 100 μM to 1 mM. This analysis led to the identification of 445 unique peptides (256 unique proteins) with a broad functional distribution (enzymes/regulatory proteins and structural proteins). (B) Live cell chemoproteomic profiling of asparagine and glutamine residues in T-47D cells. 140–259 peptides and 93–176 proteins identified across 10–100 μM of Pd­(OAc)_2_ (Supplementary Figure S7). Identification of hyperreactive Asn/Gln sites across 50 μM to 100 μM led to the identification of 203 unique peptides (141 unique proteins); values in parentheses represent the number of MS/MS peptides alone, while the total number represents MS/MS and MBR peptides. A comparison of hyperreactive Asn/Gln sites in live cells and cell lysates identified 66 common proteins and 75 unique proteins within live cell samples only. Evaluation of the localization of modified proteins showed significant membrane localization of live cell-modified proteins. (C) Gene ontology analysis of modified proteins (live cell and lysate) clearly shows significant modification of membrane-bound proteins, such as anchoring junction, cell–substrate junction, and focal adhesion proteins, in live cells over lysate-modified samples (−log_10_(FDR) > 2.8); heat scale −log_10_(FDR) for live cells (1.6–2.8); −log_10_(FDR) for cell lysate is (6–10). (D) Functional categorization of modified proteins showed a broad diversity of modified proteins, with significant modification of RNA-binding proteins with −log_10_(FDR) > 1.5 for live cells and −log_10_(FDR) > 15 for lysates. heat scale −log_10_(FDR) for live cells (1.3–1.5) and −log_10_(FDR) for cell lysate is (5–15). Created in BioRender. Lab, R. (2026) https://BioRender.com/yzdjonj.

Because intact-cell conversion can be influenced by reagent access, subcellular compartmentalization, and protein turnover, in addition to intrinsic site chemistry, we interpret these differences as access-weighted rather than definitive measures of intrinsic reactivity (Figure 2C). Functional categorization also highlighted RNA-binding proteins among the modified targets, consistent with the method’s ability to report on chemically addressable Asn/Gln sites across diverse functional classes in native cellular contexts (Figure 2D). This result is consistent with prior studies showing a high abundance of Asn/Gln in RNA-binding proteins,^28^ supporting the utility of this dehydration reaction for residue-centric profiling of Asn/Gln sites.

### Profiling and Identification of Deamidation Post-Translational Modifications

Beyond global Asn/Gln conversion mapping, we asked whether palladium-mediated amide dehydration could provide a practical, orthogonal readout for deamidation-associated changes in complex proteomes. Deamidation, a relatively understudied post-translational modification (PTM), converts Asn and Gln amides to carboxylates (Asp/Glu),^29,30^ altering charge and potentially affecting protein stability, turnover, and function.^31–35^ In proteomics workflows, deamidation is typically inferred from a small mass increase (+0.9840 Da),^36^ but this signal can be difficult to interpret in complex samples and is often confounded by sample-preparation-associated spontaneous deamidation, which inflates false discoveries. We therefore evaluated an inverse chemoproteomic strategy where the absence of chemical modification, a protection signature, signals the presence of a PTM.^37–40^ Under this framework, Asn/Gln sites that have been modified to carboxylates through deamidation will not be nitriles under dehydration conditions. This loss of reactivity serves as a diagnostic tool to identify candidate sites of biological deamidation.

To validate this inverse chemoproteomic approach, we generated dose-dependent deamidation profiles by incubating T-47D cell lysates under accelerated deamidation conditions (pH 8.8, 65 °C; Figure 3A, Supplementary Figure S8) Subsequent treatment with Pd­(OAc)_2_ (500 μM) in 4:1 NaP buffer (10 mM, pH 7.2):CH_3_CN for 2 h revealed a stark, time-dependent decrease in nitrile-modified peptides—from 7,031 in the control to 668 after 120 min of incubation—consistent with progressive conversion of amides to carboxylates and loss of chemical reactivity (Figure 3A, Supplementary Figure S8).

**Figure 3:**
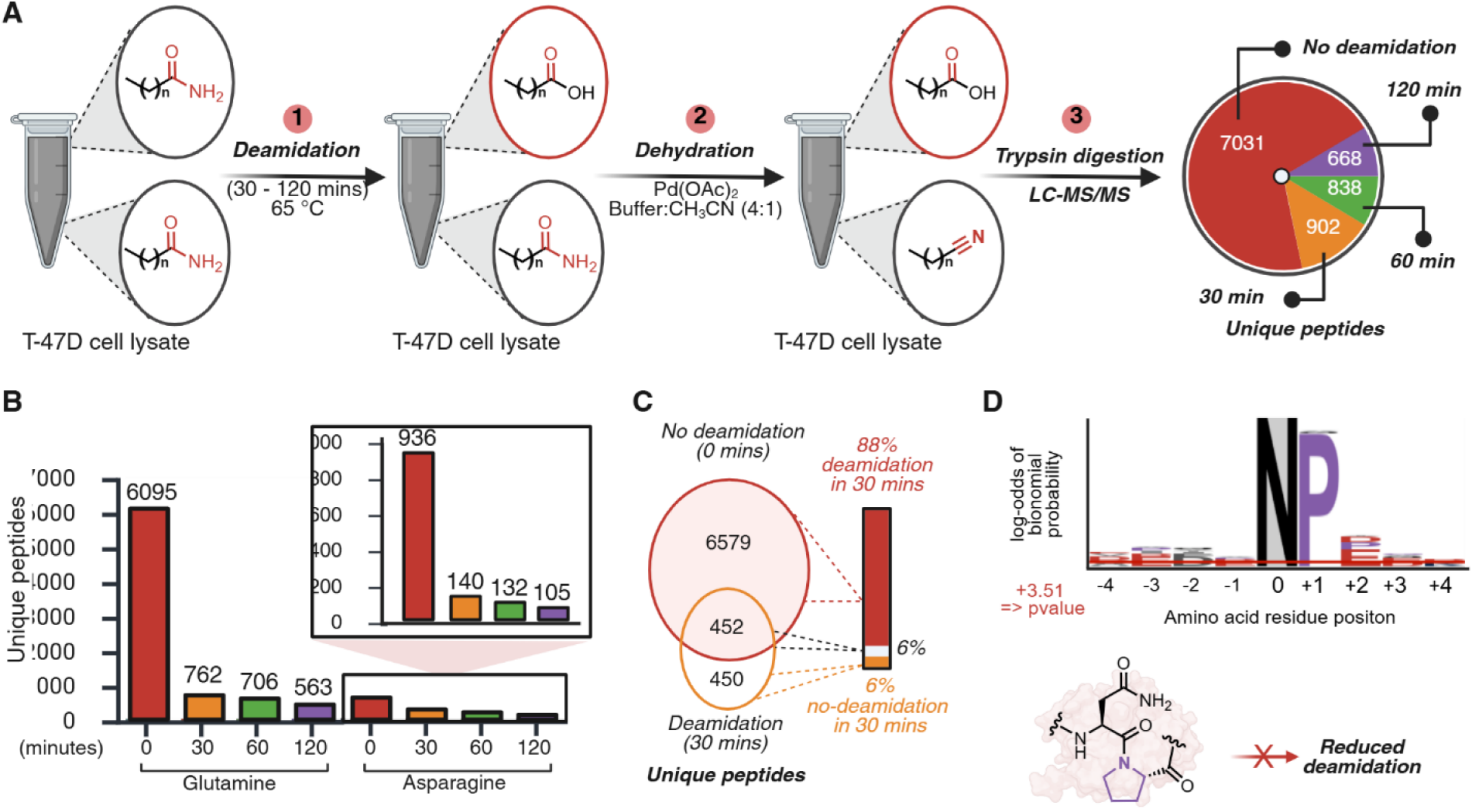
Chemoproteomics profiling of asparagine and glutamine deamidation. Note: the analysis of deamidation sites is based on the inverse correlation between nitrile modification and deamidation. (A) Schematic of the application of the dehydration reaction for chemoproteomics profiling of deamidation of Asn and Gln in T-47D cell lysate. Dose-dependent deamidation was incorporated by incubating T-47D lysates in ammonium bicarbonate buffer (pH 8.8) at 65 °C for 30, 60, and 120 min, followed by treatment with 500 μM of Pd­(OAc)_2_ in a 4:1 NaP buffer (10 mM, pH 7.2):CH_3_CN. Palladium quenching, trypsin digestion, and LC-MS/MS analysis identified the modification of 7031 unique peptides for the non-deamidated control (0 min), and 902 unique peptides for 30 min, 838 unique peptides for 60 min, and 668 unique peptides for 120 min. (B) Nitrile modification distribution of Asn and Gln across different deamidated samples. Significant modification of Gln and Asn was observed, indicating deamidation of both Asn and Gln. (C) Identification of unique deamidation sites observed after 30 min of deamidation. Nitrile peptides only observed in control samples but not in deamidated samples were identified as sites of deamidation, with an 88% deamidation rate after 30 min. (D) Sequence motif analysis of nitrile-modified Asn in the 88% deamidated set identified a predominant presence of proline adjacent to nitrile-modified Asn. Sequence motif analysis was performed by considering 4 residues from the right and left of the modified Asn. Asn was set as the fixed position with a *p*-value <0.05. Also shown is the schematic showing the plausible explanation for the predominant observation of proline adjacent to nitrile-modified Asn. Created in BioRender. Lab, R. (2026) https://BioRender.com/t49t2dr.

Across sites, nitrile formation was more frequently observed on glutamine than on asparagine, consistent with a higher apparent susceptibility of Asn to deamidation under these conditions (Figure 3B). This observation is consistent with previous literature reports where Asn is more prone to undergo deamidation due to the formation of a succinimide intermediate with a backbone.^30^ We next compared site identities between the nonheated control and the 30 min heated sample to assess how strongly the dehydration readout tracked deamidation stress. We found that 6,579 sites (88%) that were observed as nitrile modified in the control were not detected as nitrile modified after 30 min of stress (Figure 3C), consistent with widespread conversion of amides to nonreactive deamidation products.

The high fidelity of our inverse chemoproteomics strategy enabled us to uncover fundamental structural determinants of deamidation. Sequence motif analysis of the residues that remained amidated (nitrile-labeled) despite deamidation stress revealed a predominant enrichment of proline directly adjacent to Asn (Asn-Pro) (Figure 3D, Supplementary Figure S8). This pattern is consistent with established deamidation mechanisms, in which proline disfavors formation of the succinimide intermediate required for Asn deamidation, thereby reducing deamidation propensity at Asn-Pro sequences.^41^ Together, these results demonstrate that palladium-mediated amide dehydration provides a complementary chemoproteomic route to profile deamidation PTM at scale and to recover the mechanistically informative sequence context in complex proteomes.

### Tracking Asn N-Glycosylation PTM and Proteome Alterations in Yeast under Chemical Stress

To demonstrate the power of palladium-mediated dehydration of Asn/Gln in mapping complex N-glycosylation landscapes, we applied our platform to *Saccharomyces cerevisiae* due to its extensive glycosylation characteristics.^42,43^ We utilized an inverse chemoproteomic strategy to identify N-glycosylation sites on asparagine residues. To validate this, we induced ER stress using tunicamycin (TM), a potent inhibitor of N-linked glycosylation,^44,45^ to create a landscape of lower N-glycans and compared it with a control (without TM), generating high N-glycans (Figure 4A, Supplementary Figure S9).

**Figure 4:**
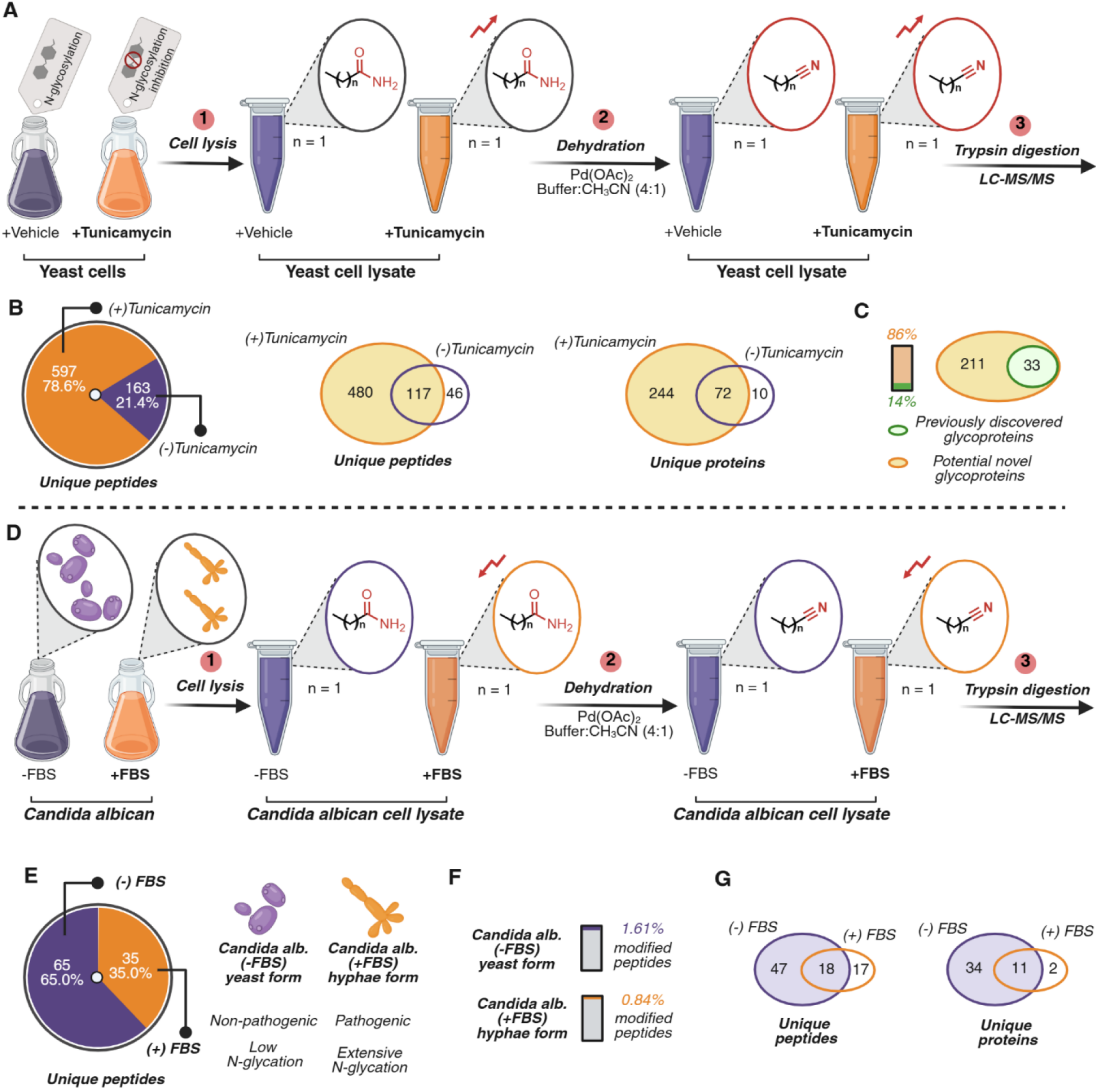
Chemoproteomics profiling of asparagine N-glycosylation and changes in proteome associated with pathogenesis. Note: the analysis of N-glycosylation sites is based on the inverse correlation between nitrile modification and N-glycosylation. (A) Schematic of the application of palladium-mediated dehydration for chemoproteomics profiling of N-glycosylation post-translational modification of Asn in yeast. Cells with downregulation of N-glycosylation were obtained by treatment with 2 μg/mL tunicamycin, followed by treatment with 500 μM Pd­(OAc)_2_ in a 4:1 NaP buffer (10 mM, pH 7.2):CH_3_CN. (B) Trypsin digestion and LC-MS/MS analysis identified Asn modification of 597 unique peptides for tunicamycin-treated samples and 163 unique peptides for nontunicamycin-treated samples, with 117 unique peptides found in both samples. Asn nitrile modification distribution across TM- and non-TM-treated samples showed extensive nitrile modification in TM-treated samples, indicative of downregulation of N-glycosylation. (C) Glycoprotein analysis of N-glycosylated proteins in both TM- and non-TM-treated samples. 33 (14%) previously reported glycoproteins were observed in TM-treated with the additional identification of 211 (86%) potentially novel glycoproteins. (D) Schematic of the palladium-mediated dehydration reaction for profiling N-glycosylation of Asn in pathogenic and nonpathogenic *Candida albicans*. Nonpathogenic yeast form of *C. albicans* and pathogenic hyphae form of *C. albicans* were generated by growing *C. albicans* with and without FBS. Cells were harvested, followed by treatment with 100 μM of Pd­(OAc)_2_ in 4:1 NaP buffer (10 mM, pH 7.2):CH_3_CN. (E) Trypsin digestion and LC-MS/MS analysis identified the modification of Asn in 65 unique peptides (65%) for nonpathogenic *C. albicans* samples and 35 unique peptides (35%) for pathogenic *C. albicans* samples. Glycoprotein schematic highlighting the glycosylation profile between nonpathogenic *C. albicans* and pathogenic *C. albicans*. (F) Evaluation of the extent of modification for yeast and hyphae forms. Comparison of nitrile-modified peptides to the total identified peptides for each group identified 1.61% of nitrile modification in yeast and 0.84% in hyphae, suggesting extensive N-glycosylation in the hyphae form of *C. albicans*. (G) Nitrile modification distribution across nonpathogenic *C. albicans* and pathogenic *C. albicans* samples. Significantly reduced Asn nitrile modification in pathogenic *C. albicans* is indicative of upregulation of N-glycosylation. Experiments presented in this figure were performed with *n* = 2 biological replicates. Created in BioRender. Lab, R. (2026). https://BioRender.com/f81fub3.

Following TM or control (DMSO) treatment, yeast were lysed and subjected to reaction conditions using 500 μM Pd­(OAc)_2_ in a 4:1 mixture of sodium phosphate buffer (10 mM, pH 7.2):CH_3_CN for 2 h, followed by palladium chelation, trypsin digestion, and LC–MS/MS analysis (Figure 4A). Consistent with increased accessibility of Asn amides upon glycosylation inhibition, we observed substantially higher numbers of nitrile-bearing peptides in TM-treated samples (597 unique peptides; 78.6%) relative to DMSO controls (163 unique peptides; 21.4%) (Figure 4B, Supplementary Figure S9), directly reflecting the loss of glycan protection at these sites. When focusing specifically on sites and proteins exhibiting patterns consistent with reduced glycan protection, TM-treated samples showed 480 unique peptides mapping to 244 proteins compared to 46 unique peptides mapping to 10 proteins in the DMSO condition (Figure 4B).

To place these TM-associated candidates in the context of existing annotations, we cross-referenced modified proteins against the GlyCosmos Glycoproteins database.^46^ Under our filtering criteria, 33 proteins (14%) matched previously reported glycoproteins, whereas 211 proteins (86%) were not annotated as glycoproteins in the database (Figure 4C, Supplementary Figure S9). This latter set can be interpreted as potential N-glycosylation-associated candidates, which may include true glycoproteins missed by enrichment-centric workflows and enzymatic tools. Gene ontology analysis of annotated and candidate proteins revealed enrichment in processes and compartments linked to secretion, ER homeostasis, and stress response (Supplementary Figure S9), consistent with the expected cellular consequences of tunicamycin treatment and unfolded protein response activation.^47,48^ Together, these data illustrate that amide dehydration provides a scalable, orthogonal route to profile site-dependent remodeling of glycosylation in eukaryotic proteomes.

### Profiling Alterations in N-Glycosylation and Proteome of *Candida Albicans* During Pathogenesis

Having established that amide dehydration can report glycosylation-associated protection patterns in yeast, we next applied the approach to the human fungal pathogen *Candida albicans*, which transitions between a commensal yeast form and a pathogenic hyphal form.^49–51^ Morphogenesis is accompanied by extensive cell-wall and secretory-pathway remodeling, and N-glycosylation has been implicated in these processes, but proteome-scale, state-dependent changes remain challenging to capture with a single workflow. We therefore used palladium-mediated amide dehydration as an orthogonal, chemistry-based readout to compare Asn-site addressability between yeast and hyphal states.

Cells grown under yeast or hyphal conditions (−FBS or + FBS) were lysed and subjected to reaction conditions using 100 μM Pd­(OAc)_2_ in a 4:1 mixture of sodium phosphate buffer (10 mM, pH 7.2):CH_3_CN, followed by palladium quenching, trypsin digestion, and LC–MS/MS analysis (Figure 4D, Supplementary Figure S10). Across data sets, we observed a shift consistent with increased glycosylation of Asn sites in the hyphal state: the hyphal condition showed fewer nitrile-modified peptides (35.0%) relative to the yeast condition (65.0%) (Figure 4E, Supplementary Figure S10). To assess whether this difference could be explained solely by broad proteome expression changes between states, we additionally evaluated the ratio of nitrile modified to unmodified peptides within each form. Consistent with increased glycosylation in hyphae, the fraction of nitrile modification was lower in the hyphal form (0.84%) than in the yeast form (1.61%; Figure 4F).

We next compared Asn-site conversion patterns between the two morphologies to nominate candidates exhibiting hyphae-associated protection signatures. This analysis identified 34 proteins comprising 47 peptides whose Asn nitrile signals were observed in the yeast state but were reduced or absent in the hypha state (Figure 4G), consistent with increased glycosylation of these sites during morphogenesis. Heatmap analysis of peptide-level modification extent further supported the global trend toward increased protection in hyphae (Supplementary Figure S10). Gene ontology analysis of proteins associated with hyphae-linked protection signatures highlighted enrichment in processes and compartments related to cell-wall organization, adhesion, and secretion (Supplementary Figure S10), consistent with known functional hallmarks of the hyphal program. Because changes in nitrile formation can reflect a combination of glycan masking, local accessibility, and protein abundance, we interpret these proteins as glycosylation-associated candidates that prioritize pathways remodeled during morphogenesis rather than as definitive assignments of site-specific N-glycosylation occupancy. Together, these data illustrate the utility of amide dehydration for comparative profiling of N-glycosylation-associated patterns across pathogenic and nonpathogenic states in *C. albicans*.

## Conclusions

This work establishes palladium-mediated amide dehydration as a scalable chemoproteomic entry point for profiling a large and comparatively underaddressed residue class: primary amides in asparagine and glutamine. By enabling the conversion of Asn/Gln side-chain amides to nitrile products through net dehydration (−H_2_O) under mild, aqueous conditions, the method provides direct LC-MS access to chemically addressable Asn/Gln sites across complex lysates and intact cells. In doing so, it expands residue-centric chemoproteomics beyond canonical nucleophiles and offers an experimentally grounded map of Asn-Gln conversion landscapes in native proteomes.

Beyond residue mapping, we introduce an inverse chemoproteomics framework in which reduced nitrile formation is used to prioritize candidate sites whose amides are masked or chemically altered, including by PTMs such as deamidation and N-glycosylation. Applied to controlled deamidation stress, this readout captured large, time-dependent losses of nitrile formation and recovered mechanistically informative sequence context, including enrichment of Asn-Pro motifs consistent with suppression of succinimide formation. In yeast, inhibition of N-linked glycosylation produced the expected shift toward increased nitrile formation and nominated an expanded set of glycosylation-associated candidates not annotated as glycoproteins in a standard resource, underscoring the complementarity of chemistry-based readouts to enrichment-centric workflows. Finally, comparative profiling in *Candida albicans* revealed morphogenesis-linked remodeling of N-glycosylation patterns and prioritized candidate proteins enriched in pathways relevant to cell-wall organization, adhesion, and secretion.

Together, these results position palladium-mediated amide dehydration as a practical complement to existing proteomic strategies, as it enables global access to Asn/Gln primary amides and provides a scalable route to prioritize PTM-associated candidates and state-dependent remodeling signatures in native systems. More broadly, the work opens a path toward residue-centric interrogation of Asn/Gln as a functional dimension of proteome regulation across physiology and disease. By converting a previously hard-to-interrogate functional group into a proteome-wide chemical readout, this platform broadens the experimental vocabulary for connecting residue chemistry to the biological state.

## Supplementary Material













## Data Availability

The mass spectrometry proteomics data generated in this study have been deposited to the ProteomeXchange Consortium via the PRIDE partner repository^52^ with the data set identifier PXD055899.

## Notes

Optimization of the reaction conditions on protein, a peptide chemoselectivity screen, and product characterization by NMR, HPLC, LC-MS, MS/MS, and HRMS (PDF)
Summary of dose-dependent chemoproteomics application of dehydration for modification of Asn/Gln sites in cell lysate. Modified Asn/Gln residues possess a mass shift of [−18.0160] right after. The protein accession numbers, peptide sequence, peptide start index number, protein full name, gene ID, intensity, and match type are shown in the table. Each concentration is represented on a different sheet. List of hyperreactive proteins found across all samples and list of Genes for GO and pLogo analysis (XLSX)
Summary of dose-dependent chemoproteomics application of dehydration for modification of Asn/Gln sites in live cells. Modified Asn/Gln residues possess a mass shift of [−18.0160] right after. The protein accession numbers, peptide sequence, peptide start index number, protein full name, gene ID, intensity, and match type are shown in the table. Each concentration is represented on a different sheet. List of hyperreactive proteins found across all samples and list of Genes for GO and pLogo analysis (XLSX)
Summary of chemoproteomics application of dehydration for profiling of deamidation post-translational modification of Asn and Gln. Modified Asn/Gln residues possess a mass shift of [−18.0160] right after. The protein accession numbers, peptide sequence, peptide start index number, protein full name, gene ID, intensity, and match type are shown in the table. Each concentration is represented on a different sheet (XLSX)
Summary of chemoproteomics application of dehydration for profiling of N-glycosylation post-translational modification of Asn. Modified Asn residues possess a mass shift of [−18.0160] right after. The protein accession numbers, peptide sequence, peptide start index number, protein full name, gene ID, intensity, and match type are shown in the table. Each concentration is represented on a different sheet (XLSX)
Summary of chemoproteomics application of dehydration for profiling of N-glycosylation profile of pathogenic and non-pathogenic yeast. Modified Asn residues possess a mass shift of [−18.0160] right after. The protein accession numbers, peptide sequence, peptide start index number, protein full name, gene ID, intensity, and match type are shown in the table. Each concentration is represented on a different sheet (XLSX)

## Abbreviations

PTM: post-translational modification
TM: tunicamycin
FBS: fetal bovine serum
