# Supplementary material for: Expanding the Chemoproteomic Toolkit to Asparagine and Glutamine
Source: ACS Chem Biol. 2026 Apr 9;21(5):1189–98. doi: 10.1021/acschembio.6c00173 (PMC13184937; doi:10.1021/acschembio.6c00173)
Supplement: Supplementary file 1 [file cb6c00173_si_001.pdf]

Supporting Information

Expanding the Chemoproteomic Toolkit to Asparagine and Glutamine

Benjamin Emenike[1], John M. Talbott[1], Zachary E. Paikin[1], Christian M. Beusch[2,3], Sohail Khoshnevis[4], David E. Gordon[2], Monika Raj[1]*

[1]Department of Chemistry, Emory University, Atlanta, GA, 30322, United States
[2]Department of Pathology and Laboratory Medicine, Emory University, Atlanta, GA, 30322 United States
[3]Department of Surgical Sciences, Uppsala University, Uppsala, 751 05, Sweden
[4]Department of Biochemistry, Emory University School of Medicine, Atlanta, GA, 30322, United States

Table of Contents

I. General.......... 2
II. Materials.......... 2
III. Analytical Methods.......... 3
IV. Fmoc Solid-Phase Peptide Synthesis (Fmoc-SPPS).......... 3
V. Purification.......... 3
VI. Cell Culture Technique.......... 4
Figure S1: Small Molecule Characterization and Mechanism for Nitrile Formation.......... 4
Figure S1a: ^{1}H NMR of 2-Phenylacetonitrile.......... 5
Figure S1b: ^{13}C NMR of 2-Phenylacetonitrile.......... 5
Figure S1c: Proposed Mechanism for Nitrile Formation.......... 6
Figure S2: Peptide Validation of Asn/Gln Dehydration to Nitrile.......... 6
Figure S2a: HPLC Trace for H_2N-WRFNGLRG-CO_2H.......... 7
Figure S2b: HRMS for H_2N-WRFNGLRG-CO_2H.......... 7
Figure S2c: HPLC Trace for H_2N-WRFN(CN)GLRG-CO_2H.......... 8
Figure S2d: HRMS for H_2N-WRFN(CN)GLRG-CO_2H.......... 8
Figure S2e: MS/MS of H_2N-WRFN(CN)GLRG-CO_2H.......... 9
Figure S2f: HPLC Trace for H_2N-KYWCSMEHR-CO_2H.......... 10
Figure S2g: HRMS for H_2N-KYWCSMEHR-CO_2H.......... 10
Figure S2h: Crude HPLC Trace for Nitrile Formation Chemoselectivity.......... 11
Figure S3: Asn/Gln Nitrile Formation on Myoglobin.......... 11
Figure S3a: Intact MS of Modified Myoglobin –1 mM $Pd(OAc)_2$.......... 12
Figure S3b: Deconvoluted MS of Modified Myoglobin – 1 mM $Pd(OAc)_2$.......... 13
Figure S3c: Intact MS of Modified Myoglobin –2 mM $Pd(OAc)_2$.......... 13
Figure S3d: Deconvoluted MS of Modified Myoglobin –2 mM $Pd(OAc)_2$.......... 14
Figure S3e: Intact MS of Modified Myoglobin –3 mM $Pd(OAc)_2$.......... 14
Figure S3f: Deconvoluted MS of Modified Myoglobin –3 mM $Pd(OAc)_2$.......... 15
Figure S3g: Intact MS of Modified Myoglobin –5 mM $Pd(OAc)_2$.......... 15

Figure S3h: Deconvoluted MS of Modified Myoglobin –5 mM $Pd(OAc)_2$ 16
Figure S3i: Intact MS of Modified Myoglobin –10 mM $Pd(OAc)_2$ 16
Figure S3j: Deconvoluted MS of Modified Myoglobin –10 mM $Pd(OAc)_2$ 17
Figure S3k: MS/MS Analysis of Digested Myoglobin 17
Figure S4: Activity Assay Using Modified Myoglobin 21
Figure S4a: Absorbance spectra of oxidation of *o*-phenylenediamine 22
Figure S5: Nitrile Formation Dose-Dependent Proteomics – Lysate Level 22
Figure S5a: Mass Change Specificity towards Asn/Gln 24
Figure S5b: Mass Change Search Histogram 25
Figure S5c: Representative y and b ion spectra 26
Figure S5d: Hyper-reactive Asn/Gln sites 30
Figure S5e: Sequence motif of modified Asn/Gln sites 30
Figure S5f: Gene Ontology (GO) Analysis 31
Figure S6: Cell Viability Studies 32
Figure S6a: Flow gating protocol 34
Figure S6b: Cell viability with varying ACN dosage 34
Figure S6c: Representative flow cytometry spectra 35
Figure S6d: Cell viability with varying Pd dosage 36
Figure S6e: Representative flow cytometry spectra 37
Figure S7: Nitrile Formation Dose-Dependent Proteomics – Live Cells 37
Figure S7a: Representative y and b ion spectra 38
Figure S7b: Hyper-reactive Asn/Gln sites 40
Figure S7c: Gene Ontology (GO) Analysis 40
Figure S8: Nitrile Dehydration for Profiling Deamidation Post-Translational Modifications 41
Figure S9: Nitrile Dehydration for Profiling N-Glycosylation Post-Translational Modification. 43
Figure S9a: Gene Ontology (GO) Analysis 45
Figure S10: Nitrile Dehydration for Profiling N-Glycosylation in Pathogenic and Non-Pathogenic Yeast 47
Figure S10a: HeatMap analysis of N-glycosylation Ratio 49
Figure S10b: Gene Ontology (GO) Analysis 49
References 51

I. General.
All commercial materials (Sigma-Aldrich, Ambeed, and ThermoFisher) were used without further purification. All solvents were reagent or HPLC (Fisher) grade. Percent conversions refer to chromatographically pure compounds. Reaction progress was monitored by TLC plates (TLC Silica gel 60 F_{254}) and visualized with UV lamps.

II. Materials.
Fmoc-amino acids, Rink amide resin, and hexafluorophosphate benzotriazole tetramethyl uronium (HBTU) were obtained from CreoSalus (Louisville, Kentucky). Wang resin was obtained from Sigma Aldrich (St. Louis, Missouri). N,N′-diisopropylethylamine (DIPEA), 3-phenylpropionitrile, and 2,2'-bipyridyl ligand was obtained from TCI (Portland, Oregon). Piperidine and trifluoroacetic acid (TFA) were obtained from Alfa Aesar (Ward Hill, Massachusetts). N,N-dimethylformamide (DMF), dichloromethane (DCM), methanol (MeOH),

and acetonitrile (ACN) were obtained from VWR (100 Matsonford Road Radnor, Pennsylvania). All other small molecules were obtained from Sigma and Combi-Blocks (San Diego, California).

III. Analytical Methods

NMR: NMR spectra were recorded on a 400 MHz or 600 MHz Bruker NMR spectrometer. Proton chemical shifts were referenced to residual $CDCl_3$ at 7.26 ppm and carbon chemical shifts were referenced to $CDCl_3$ at 77.16 ppm. Spectra were processed using MestReNova ver. 12.0.4 and TOPSPIN software. The following abbreviations (or combinations thereof) are used to refer to multiplicities: s = singlet, d = doublet, t = triplet, q = quartet, p = quintet, and m = multiplet. Coupling constants (*J*), are reported in Hertz units (Hz).

HPLC: Peptide reactions were analyzed using high performance liquid chromatography (HPLC) on an Agilent 1100 series equipped with a 5 µm particle size, C-18 reversed-phase column. All separations involved a mobile phase of water with 0.1% formic acid (solvent A) and acetonitrile with 0.1% formic acid (solvent B) with a flow rate of 1 mL/min. The eluent was monitored by absorbance at 220 nm and 254 nm. **HPLC METHOD A:** Gradient: 2-80 % B over 30 min. **HPLC METHOD B:** Gradient: 2-60 % B over 30 min.

HRMS. High resolution MS data were acquired on Thermo Exactive Plus using a heated electrospray source. The solution was infused at a rate of 10-25 µL min^{-1} electrospray using 3.3 kV. The typical settings were Capillary temp 320 °C. S-lens RF level was between 30-80 with an AGC setting of 1 E6. The maximum injection time was set to 50 ms. Spectra were taken at 140,000 resolutions at m/z 200 using Tune software and analyzed with ThermoFischer's Freestyle software. ver. 1.8.63.0.

IV. Fmoc Solid-Phase Peptide Synthesis (Fmoc-SPPS).[1]

Peptides were synthesized using standard protocols. Peptides were synthesized manually on a 0.25 or 0.40 mmol scale using Rink amide resin or Wang resin. Resin was swollen with DCM for 1 hour at RT. Fmoc was deprotected using 20 % piperidine in DMF for 30 min to obtain a deprotected resin. Fmoc protected amino acid (1.25 mmol or 2.00 mmol, 5 equiv.) was coupled using HBTU (1.25 mmol or 2.00 mmol, 5 equiv.) and DIPEA (1.25 mmol or 2.00 mmol, 5 equiv.) in DMF for 25 min at RT. Fmoc deprotection was achieved using 20% piperidine in DMF for 20 min at RT. Peptides were cleaved from the resin using 10 mL of a cocktail consisting of 95:2.5:2.5 trifluoroacetic acid : water : triethylsilane for 2 hours. The resin was removed by filtration and the resulting solution was concentrated via air. Peptides were precipitated and centrifugated with cold diethyl ether (3 x 10 mL) to obtain the crude product. Crude peptides were dissolved in ACN:H_2O and purified by preparatory HPLC.

V. Purification

Purification of peptide starting materials was performed using high performance liquid chromatography (HPLC) on an Agilent 1100 series HPLC equipped with a C-18 reverse phase column with a particle size of 5 µm or Teledyne ISCO ACCQ Prep HP150 equipped with a C-18 reverse phase 9.4x250 mm column with a particle size of 5 µm. All separations involved a mobile phase of 0.1 % formic acid in water (solvent A) and 0.1 % formic

acid in acetonitrile (solvent B). The HPLC method used a linear gradient at RT with a flow rate of 1 mL min^{-1}. The eluent was monitored by absorbance at 220 nm and 254 nm.

VI. Cell Culture Technique

Cells were maintained at 37 °C and 5% CO_2. T-47D cells were cultured in RPMI 1640 media supplemented with 10% (V/V) fetal bovine serum (FBS) and 1% (V/V) penicillin/streptomycin (100 µg/mL).

Cell Lysis. Whole cell lysate was generated by lysing cells on ice in RIPA buffer (50 mM TrisHCl [pH 8], 150 mM NaCl, 1% NP-40, 0.5% sodium deoxycholate, 0.1% SDS) supplemented with protease and phosphatase inhibitors. Lysates were centrifuged 6,500 x g, 10 m at 4 °C, and soluble lysate was collected. Whole cell lysate proteins were separated using 16% SDS-PAGE. SDS-PAGE gels were stained with Coomassie brilliant blue dye.

Figure S1: Small Molecule Characterization and Mechanism for Nitrile Formation

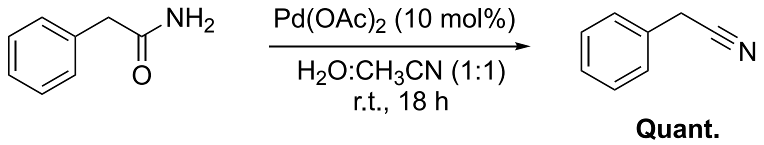

2-phenylacetamide (1.00 g, 6.7 mmol, 1 equiv.) was added to a 25 mL RBF and dissolved in 12 mL of 1:1 H_2O:ACN. Then, $Pd(OAc)_2$ (172 mg, 0.67 mmol, 0.1 equiv.) was added and stirred at room temperature for 18 hours. Upon completion as monitored by TLC, ethylene diamine (500 µL) was added to quench the reaction. The reaction mixture was extracted with EtOAc (2 x 50 mL) and the organic layer was dried over Na_2SO_4, concentrated, and purified by column chromatography (5 x 17 cm column, 100% EtOAc, 18 mL fractions) to afford compound 2-phenylacetonitrile as a clear liquid (866 mg, quant.). Analytical TLC, EtOAc eluent, Rf = 0.69. **^{1}H NMR** (400 MHz, $CDCl_3$): δ = 7.42-7.30 (5H, m), 3.76 (2H, s) ppm. **^{13}C NMR** (101 MHz, $CDCl_3$) δ: 130.0, 129.3, 128.2, 128.1, 118.0, 23.8. Matches literature spectra.

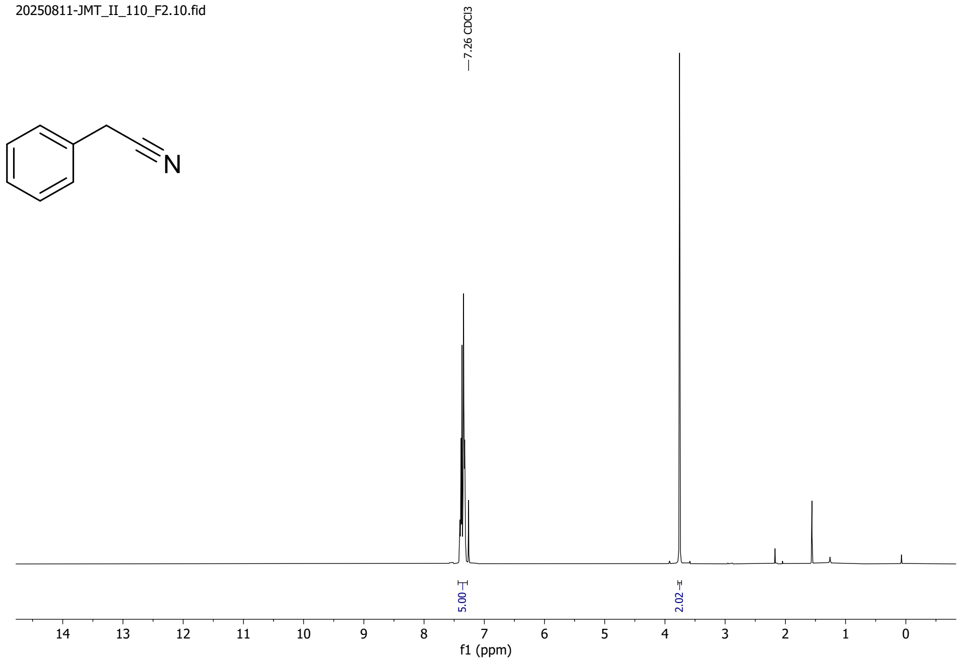

Figure S1a: ^{1}H NMR of 2-Phenylacetonitrile

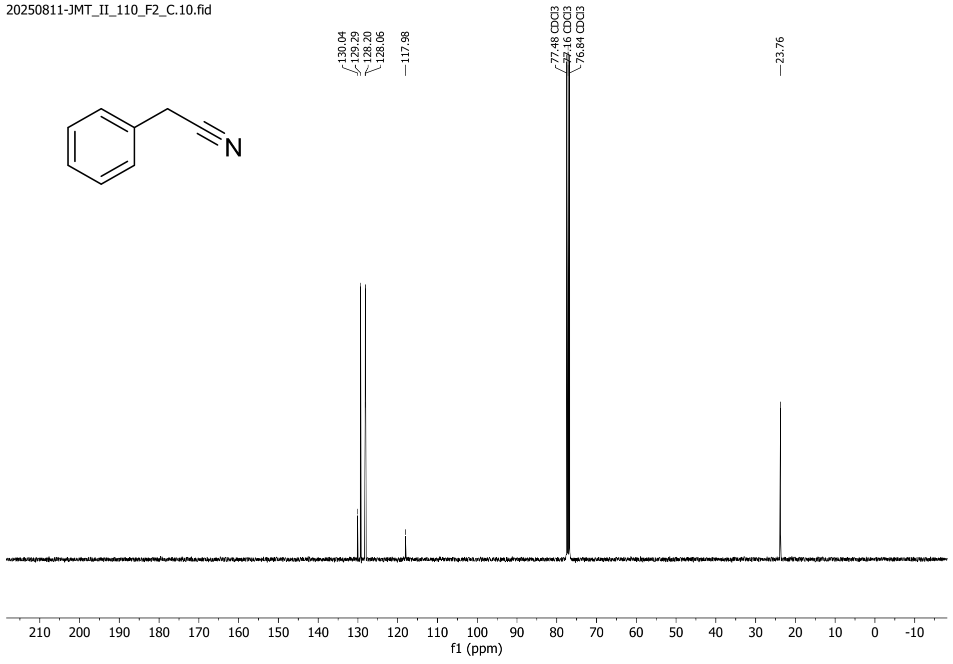

Figure S1b: ^{13}C NMR of 2-Phenylacetonitrile

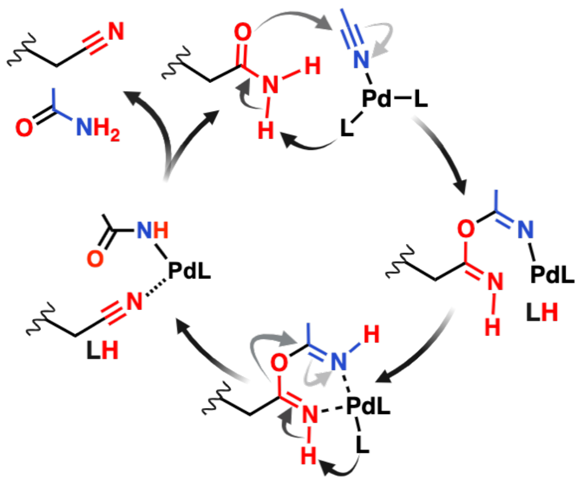

Figure S1c: Proposed Mechanism for Nitrile Formation

Figure S2: Peptide Validation of Asn/Gln Dehydration to Nitrile

To validate formation of nitrile on peptides, we first synthesized peptide H_2N-WRFNGLRG-CO_2H (**S1**) via Fmoc-SPPS.

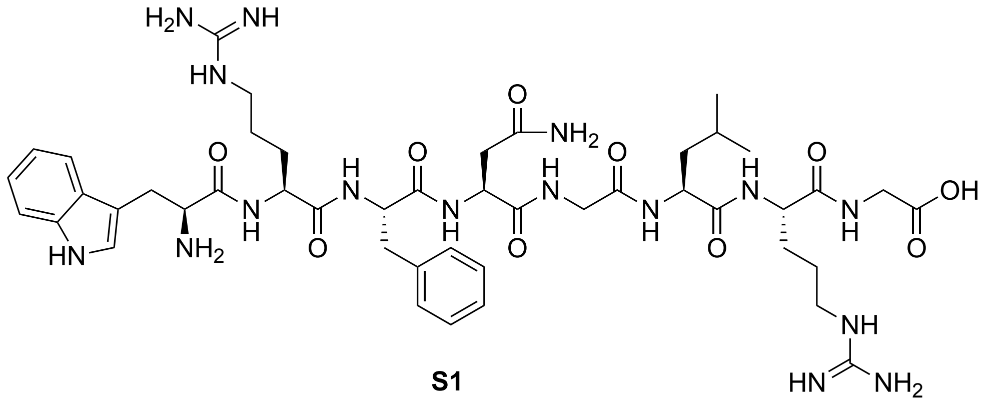

S1

H_2N-WRFNGLRG-CO_2H (S1). LCMS, m/z 1005.5404 (calcd. $[M+H^+]$ = 1005.5377), m/z 503.2741 (calcd. $[(M+2H^+)/2]$ = 503.2725), Purity: > 99% (HPLC analysis at 220 nm). Retention time in HPLC: 10.5 min.

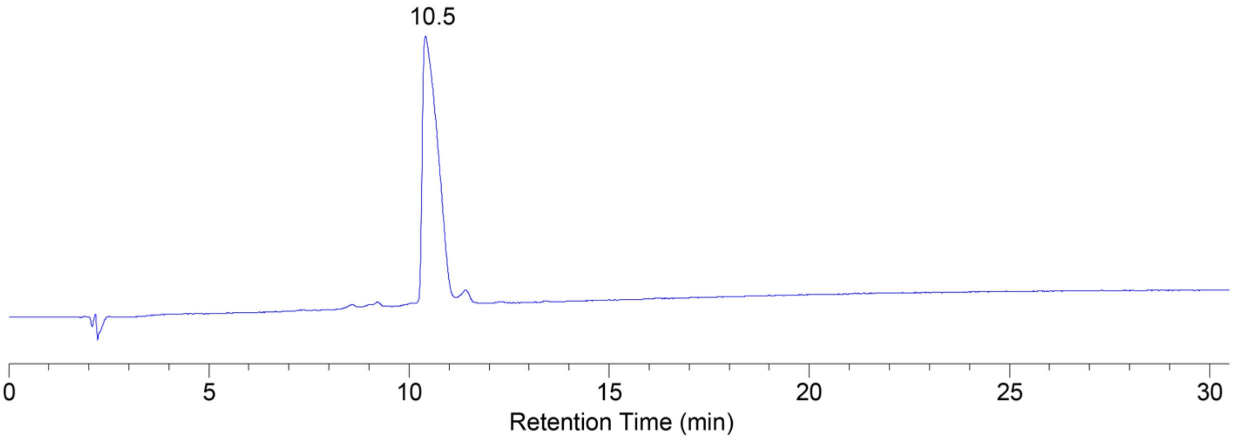

Figure S2a: HPLC Trace for H_2N-WRFNGLRG-CO_2H

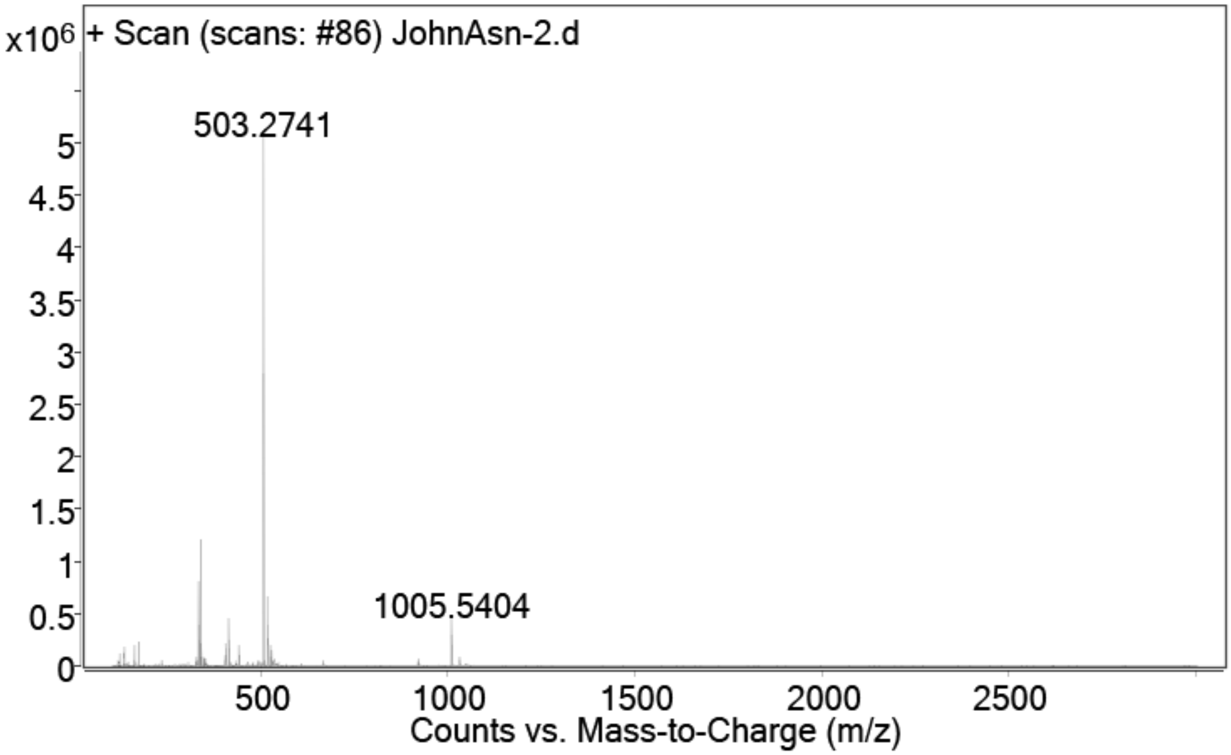

Figure S2b: HRMS for H_2N-WRFNGLRG-CO_2H

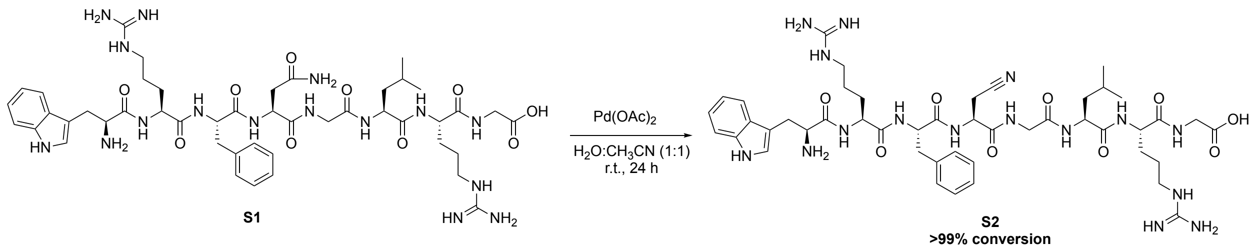

H_2N-WRFNGLRG-CO_2H (**S1**) (1.0 mg, 1 equiv.) was dissolved in 600 µL of 1:1 H_2O:ACN in a 1/2” dram vial. Next, 3 equiv. of $Pd(OAc)_2$ was added in one portion. The reaction was stirred at room temperature for 24 h and analyzed via **HPLC Method A** to determine percent conversion to **S2,** >99% conversion.

H_2N-WRFN(CN)GLRG-CO_2H peptide (S2). LCMS, m/z 987.5278 (calcd. [$M+H^+$] = 987.5271), m/z 494.2702 (calcd. [$(M+2H^+)/2$] = 494.2672), Purity: > 99% (HPLC analysis at 220 nm). Retention time in HPLC: 11.8 min.

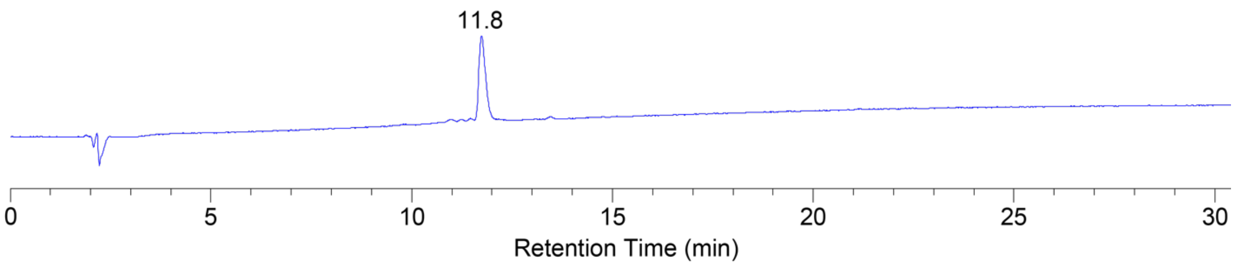

Figure S2c: HPLC Trace for H_2N-WRFN(CN)GLRG-CO_2H

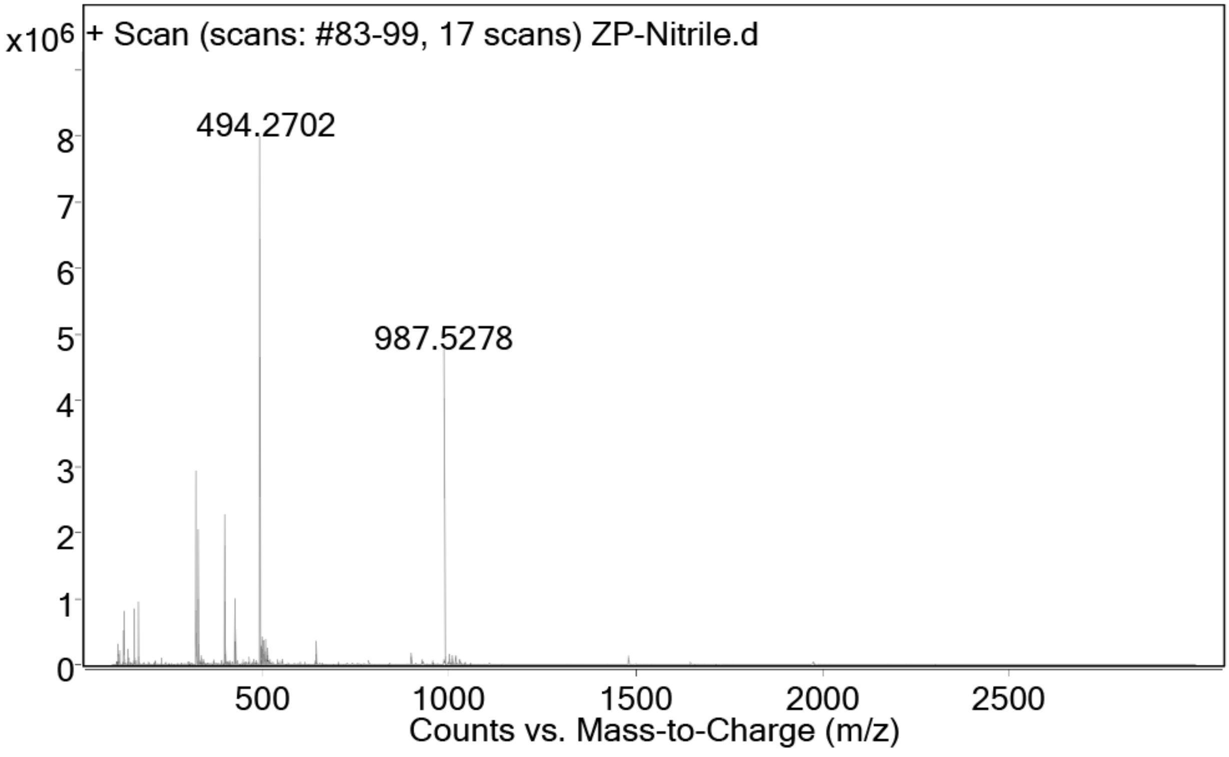

Figure S2d: HRMS for H_2N-WRFN(CN)GLRG-CO_2H

Figure S2e: MS/MS of H_2N-WRFN(CN)GLRG-CO_2H

Biomolecule 1: WRFNGLRG

Biomol	Seq Loc	Rule	Pred Mods	RT	Height	Mass	Tgt Mass	Diff (ppm)
1	A(1-8)	Complete digest, Predicted modifications	dehydration 4	3813.000	3039215	986.5277	986.5199	7.96

ECC (with sample chromatogram)

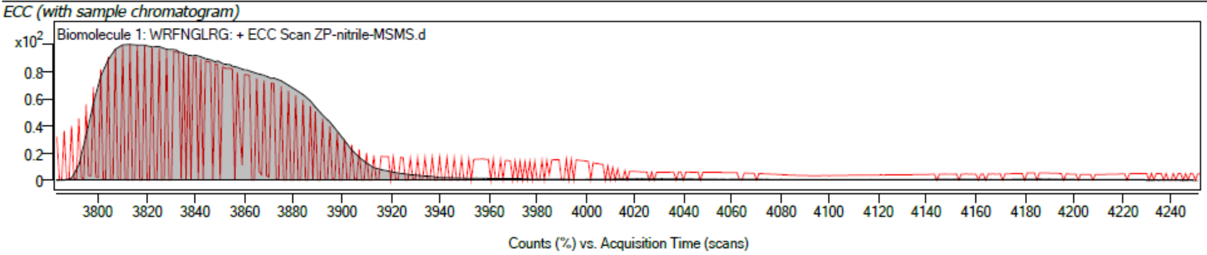

Mass Spectrum (with MFE spectrum, if available)

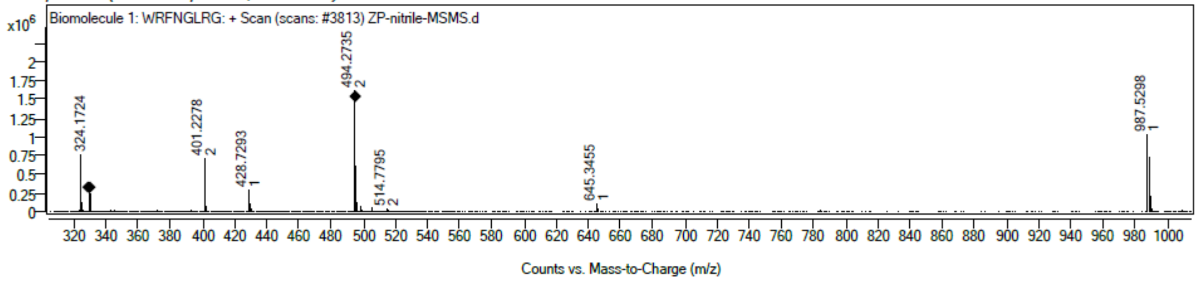

Fragment Spectra (if available)

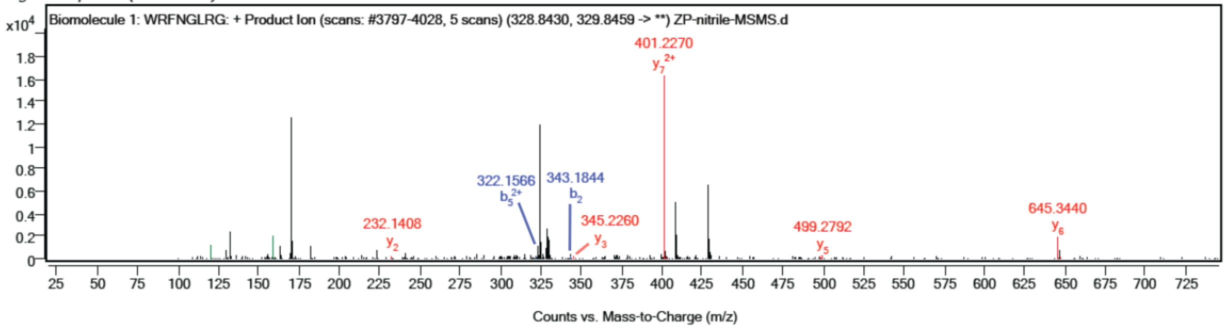

Fragment Spectrum Peaks

m/z	Diff (ppm)	Abund	Ion	Z
232.1408	-1.74	171	y2	
345.2260	-4.46	193	y3	
499.2792	4.93	254	y5	
645.3440	4.14	1934	y6	
401.2270	1.44	16255	y7	
343.1844	9.68	199	b2	
322.1566	6.25	83	b5	
120.0804	3.15	1221	F	
159.0910	4.36	1981	W	
329.8462	3.26	1655	Precursor	
330.1813	1.28	1374	Precursor	
330.5130	9.46	260	Precursor	

Chemoselectivity
To validate the chemoselectivity of Asn/Gln dehydration to nitrile, we took a peptide H_2N-KYWCSMEHR-CO_2H (S3), containing all reactive amino acid side chains, and incubated it with 2 equiv. of $Pd(O_2CCF_3)_2$ for 24 hours. No modification of any residues was observed.

Synthesis of H_2N-KYWCSMEHR-CO_2H (S3)

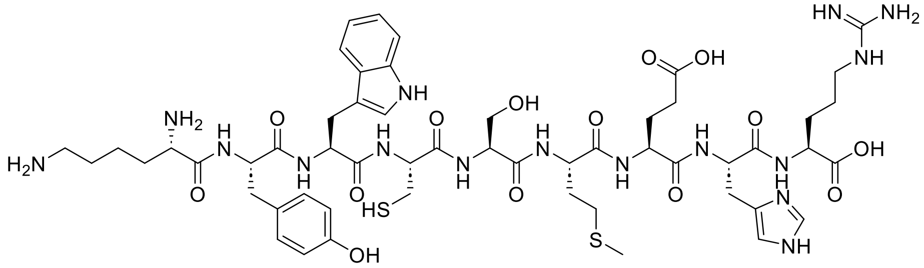

H_2N-KYWCSMEHR-CO_2H (S3): LCMS, *m/z* 1239.5383 (calcd. [M+H^+] = 1239.5398), *m/z* 620.2731 (calcd. [(M+2H^+)/2] = 620.2735), *m/z* 413.8512 (calcd. [(M+3H^+)/3] = 413.8514), Purity: >99% (HPLC analysis at 220 nm). Retention time using **HPLC Method B**: 9.8 min.

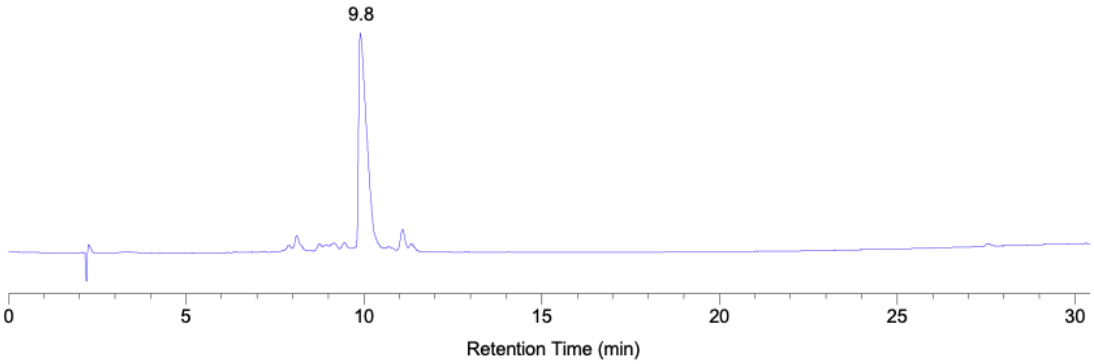

Figure S2f: HPLC Trace for H_2N-KYWCSMEHR-CO_2H

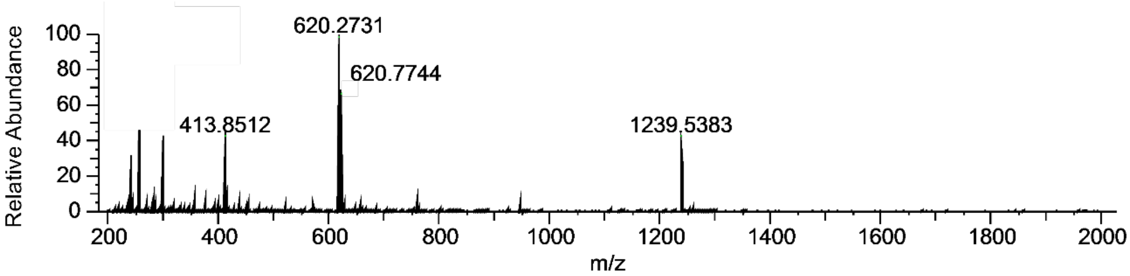

Figure S2g: HRMS for H_2N-KYWCSMEHR-CO_2H

Chemoselectivity Control: Nitrile Formation Conditions

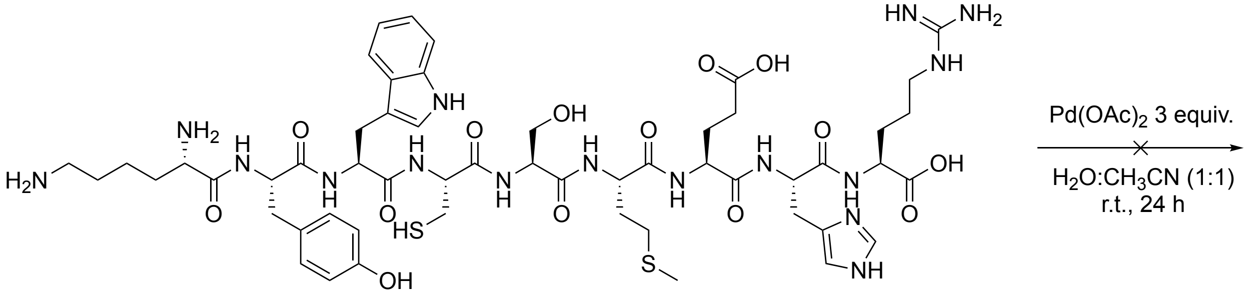

H_2N-KYWCSMEHR-CO_2H (S3) 1 mg (0.8 µmol, 1 equiv.) was dissolved in 600 µL of 1:1 H_2O:ACN in a 1/2" dram vial. Next, Pd(OAc)$_2$ (359 µg, 1.6 µmol, ~2.7 mM, 2 equiv.) was added from a 10 mM stock solution in ACN. The vial was stirred at room temperature for 24 hours then quenched with 3-MPA (1.4 µL, 16 µmol, 20 equiv.). The reaction was analyzed via **HPLC Method A**. No conversion occurred.

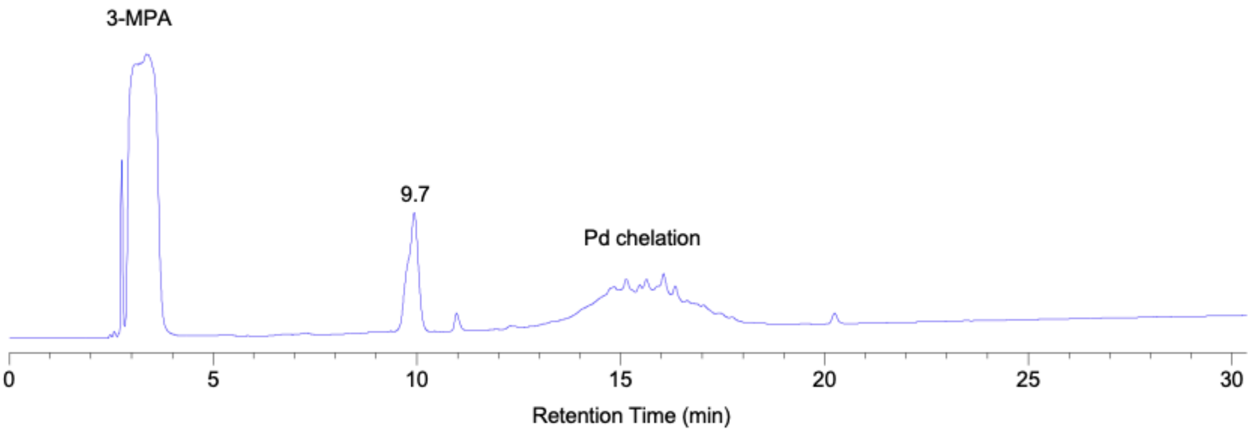

Figure S2h: Crude HPLC Trace for Nitrile Formation Chemoselectivity

Figure S3: Asn/Gln Nitrile Formation on Myoglobin

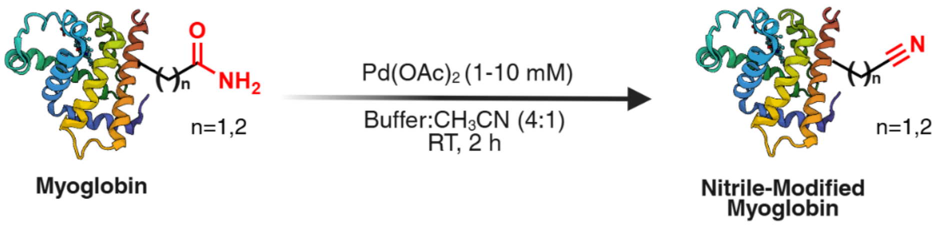

Myoglobin (2 mg, 0.118 mM) was dissolved in 800 µL of NaP buffer (10 mM, pH 7.2) and 200 µL of Pd(OAc)$_2$ (1-10 mM) dissolved in ACN was added. The reaction was stirred at room temperature for 2 h followed by quenching with 500 µL of 1 M solution of aqueous L-cysteine

and 10 µL of 1 M NaOH solution. The crude reaction mixture was passed through an Amicon™ Ultra 3 kDa centrifugal filter and washed with H_2O (7×0.5 mL) to remove the water-soluble Pd complex. The labeled protein was redissolved in 0.1% formic acid in H_2O and analyzed using LC-MS to determine conversion to nitrile (see table below). The "singly-modified" sample (3 mM $Pd(OAc)_2$) was additionally digested using SMART Digest™ Trypsin Kit by Thermo Scientific and analyzed by LC-MS/MS, with Q26 and Q128 identified as the sites of nitrile modification.

Table S1: Modification of Myoglobin with varying palladium concentrations

Concentration of $Pd(OAc)_2$	Unmodified Protein	1 Nitrile	2 Nitrile	≥ 3 Nitrile	Overall Conversion
1 mM (0.22 mg)	>95%	n.d.	n.d.	n.d.	<5%
2 mM (0.45 mg)	79%	21%	n.d.	n.d	21%
3 mM (0.67 mg)	43%	45%	12%	n.d.	57%
5 mM (1.12 mg)	17%	40%	30%	13%	83%
10 mM (2.25 mg)	n.d.	11%	24%	65%	>95%

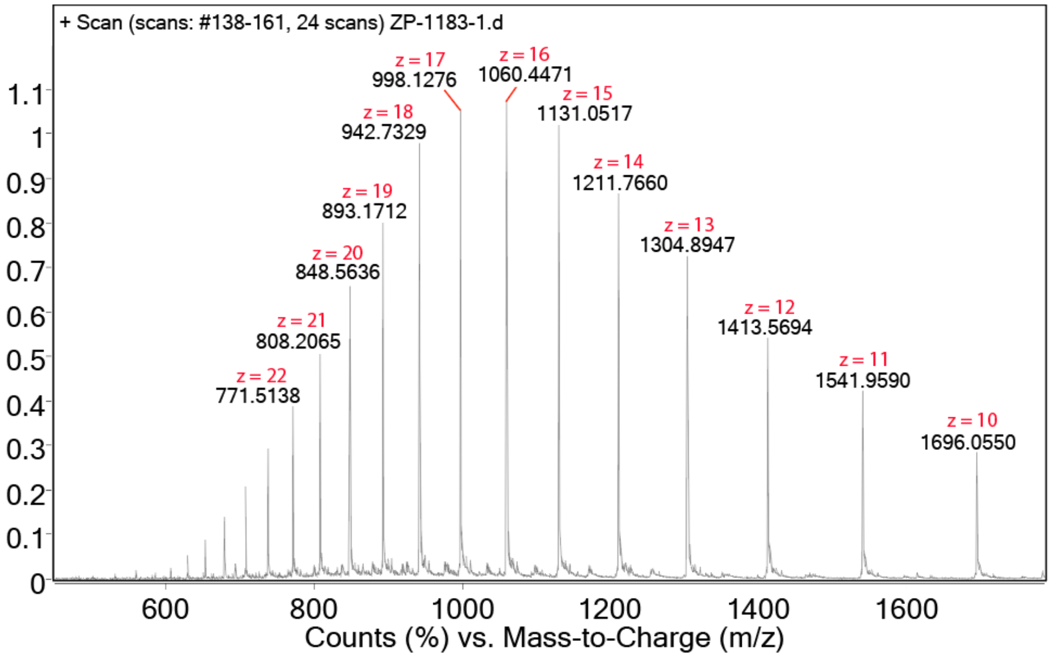

Figure S3a: Intact MS of Modified Myoglobin –1 mM $Pd(OAc)_2$

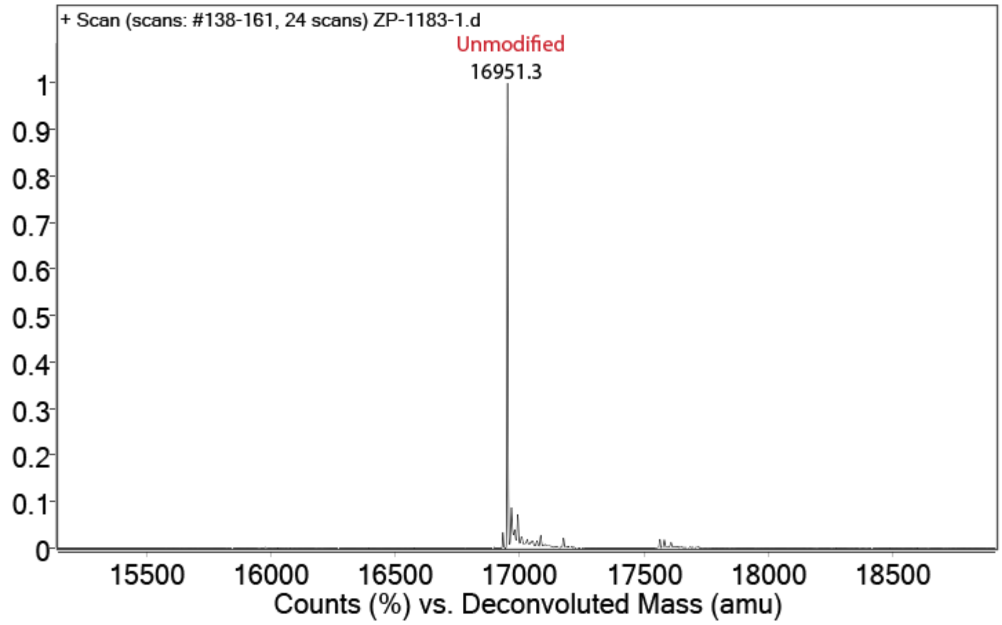

Figure S3b: Deconvoluted MS of Modified Myoglobin – 1 mM Pd(OAc)$_2$

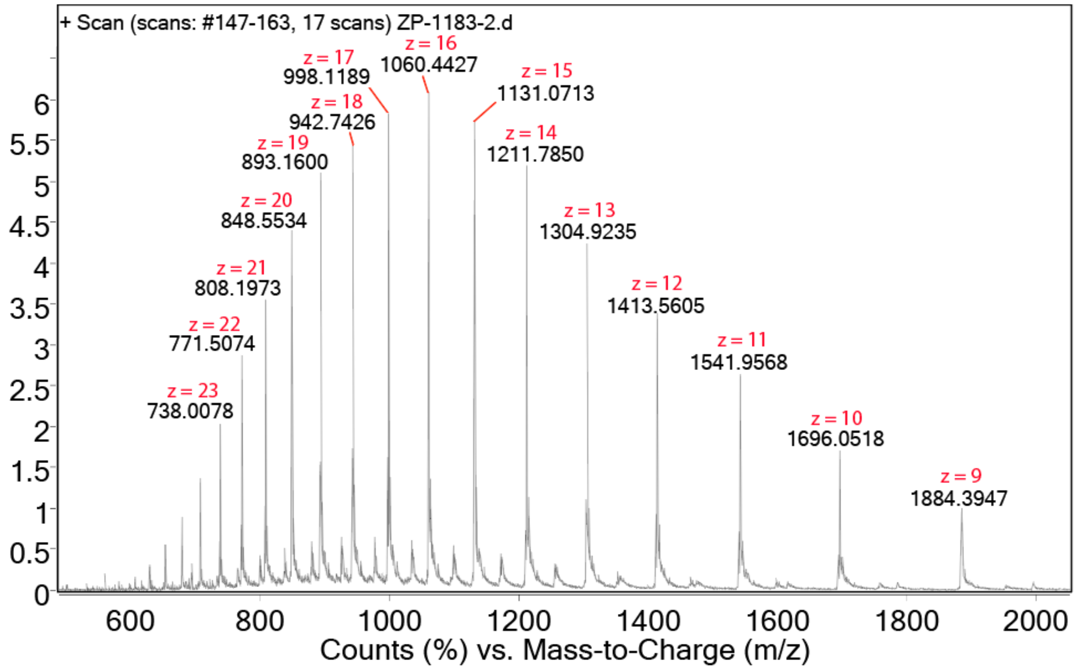

Figure S3c: Intact MS of Modified Myoglobin –2 mM Pd(OAc)$_2$

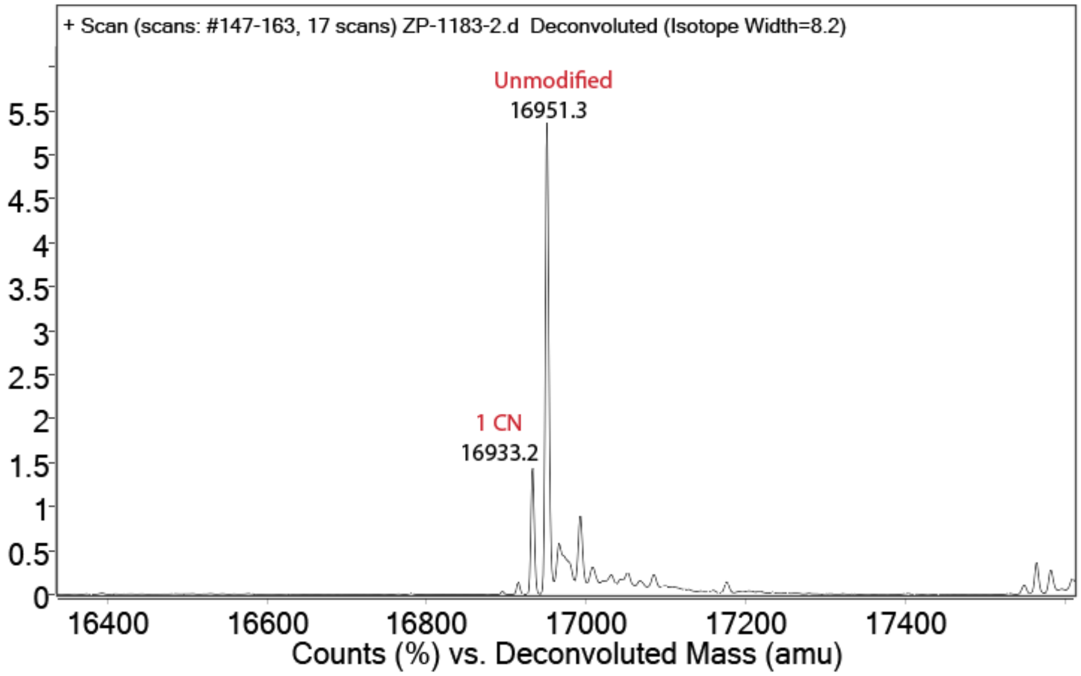

Figure S3d: Deconvoluted MS of Modified Myoglobin –2 mM Pd(OAc)$_2$

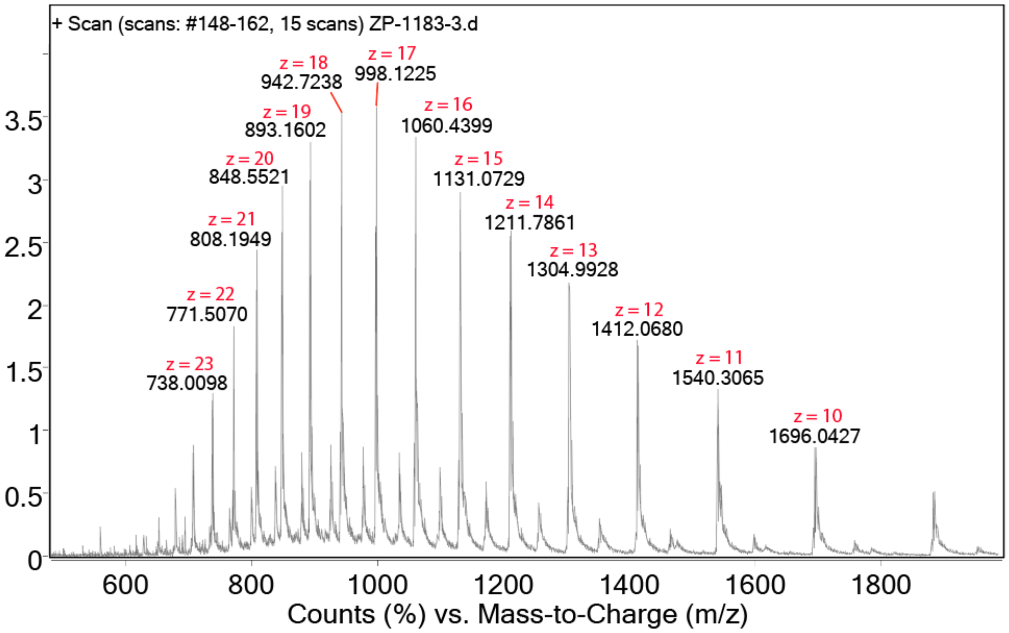

Figure S3e: Intact MS of Modified Myoglobin –3 mM Pd(OAc)$_2$

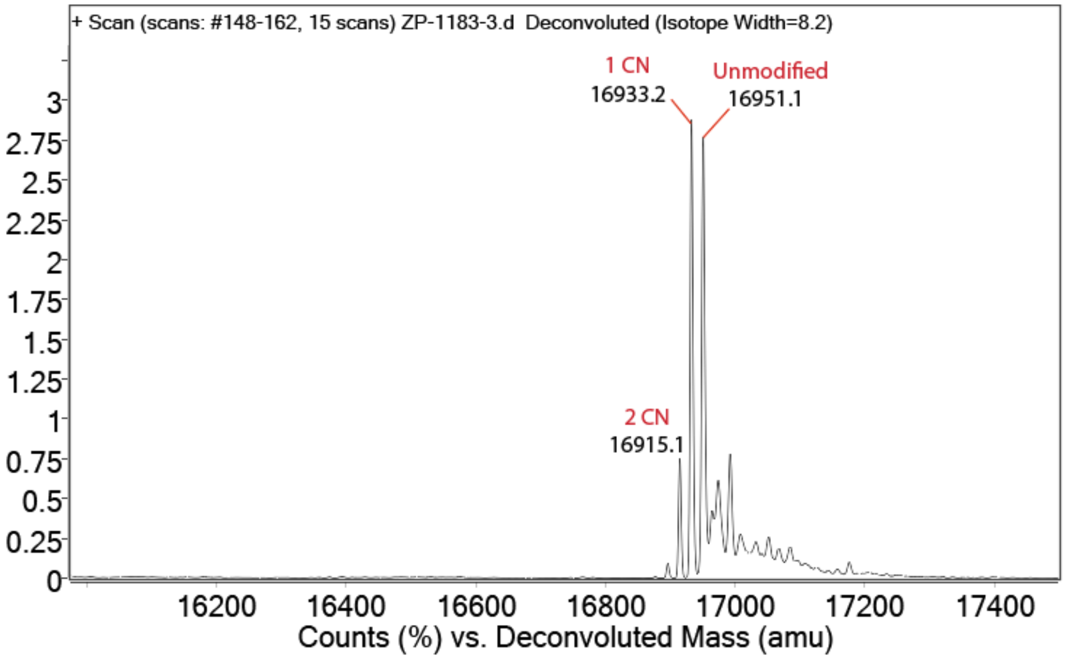

Figure S3f: Deconvoluted MS of Modified Myoglobin –3 mM Pd(OAc)$_2$

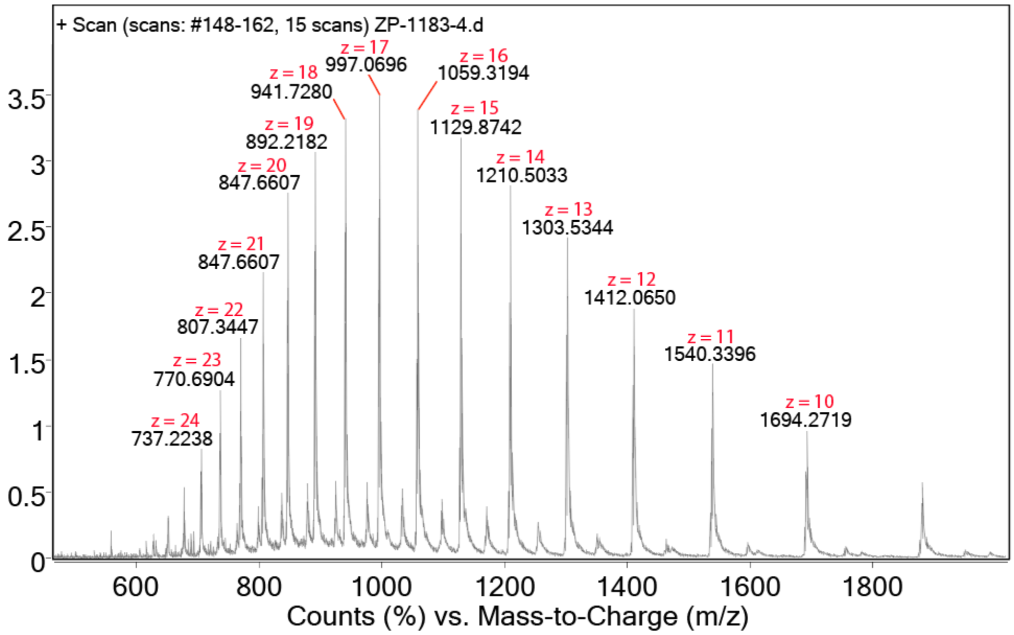

Figure S3g: Intact MS of Modified Myoglobin –5 mM Pd(OAc)$_2$

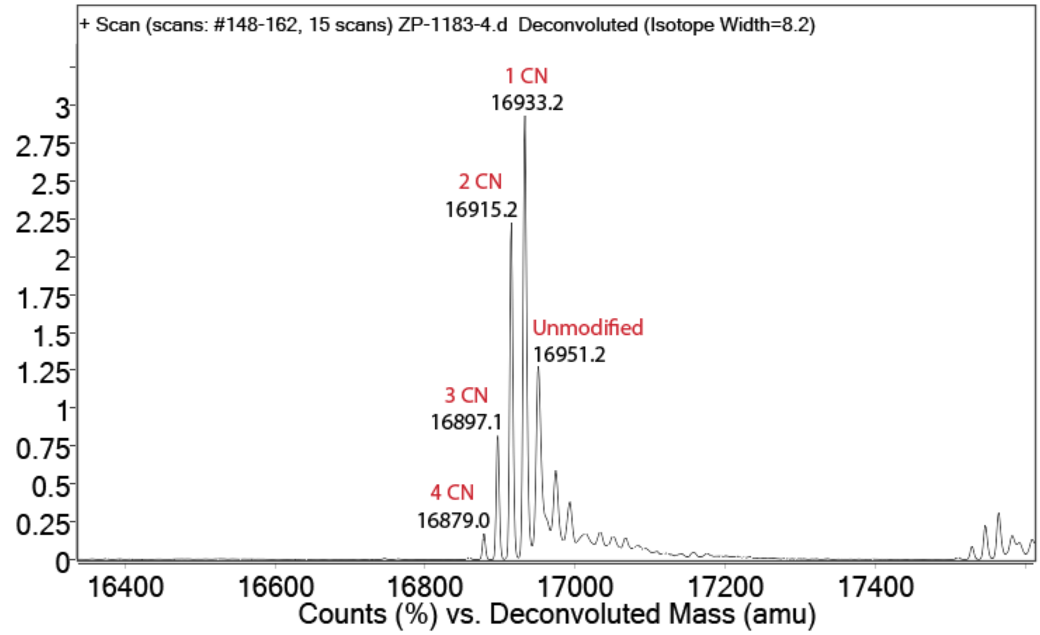

Figure S3h: Deconvoluted MS of Modified Myoglobin –5 mM Pd(OAc)₂

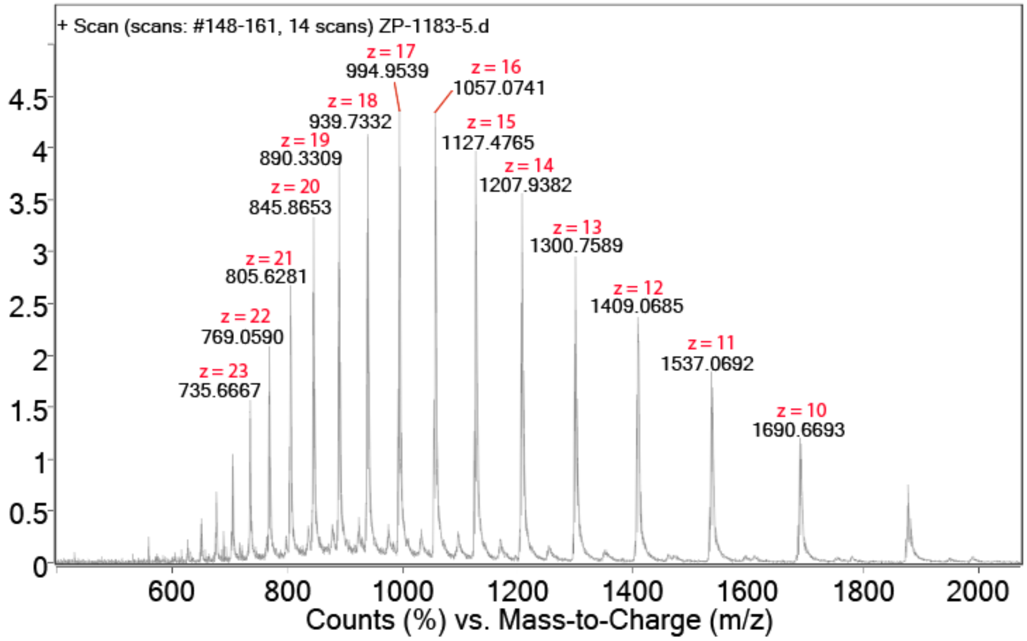

Figure S3i: Intact MS of Modified Myoglobin –10 mM Pd(OAc)₂

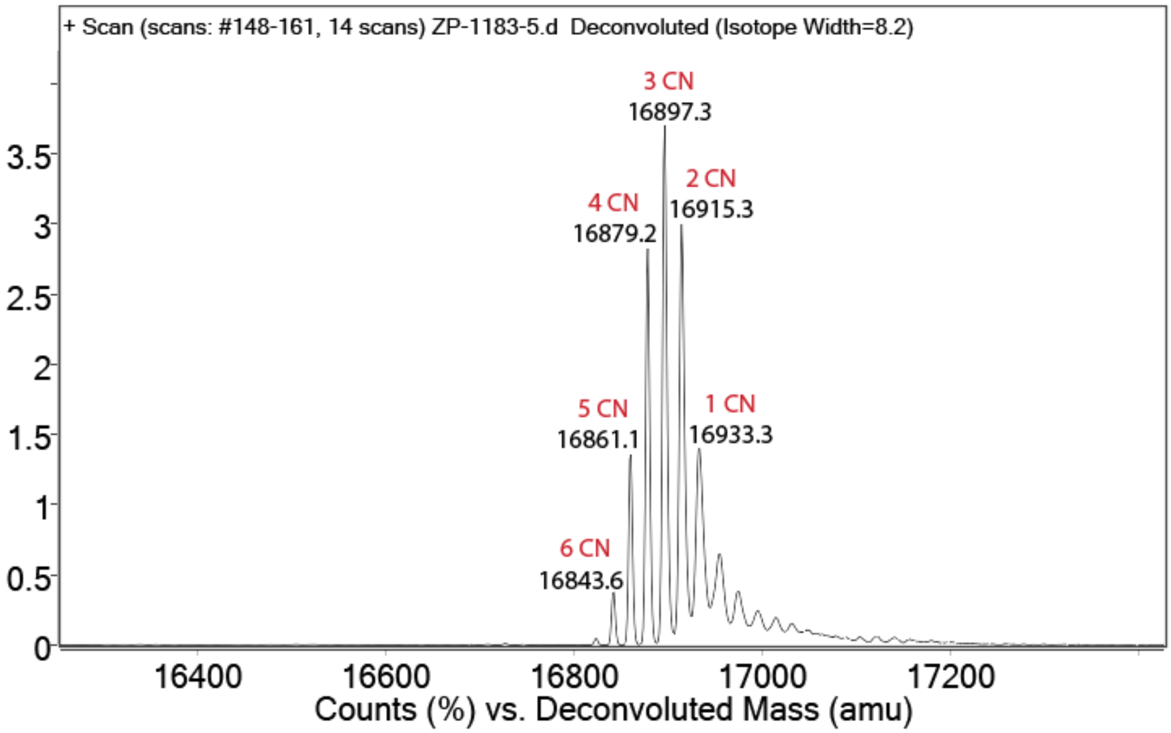

Figure S3j: Deconvoluted MS of Modified Myoglobin –10 mM $Pd(OAc)_2$

Figure S3k: MS/MS Analysis of Digested Myoglobin

(Singly Modified by 3 mM $Pd(OAc)_2$)

Sequence Summary

Confirmation Status	% Coverage	Sequence Name
Confirmed	100.00	1WLA\MYOGLOBINE\Equus caballus

Identified peptide fragment ***(1 site)****: GLSDGEWQQVLNVWGK (Sequence: AA 1-16, Q8)*

Biomol	Seq Loc	Rule	Pred Mods	RT	Height	Mass	Tgt Mass	Diff (ppm)
30	A(1-16)	Complete digest, Predicted modifications	dehydration 8	5142.000	207144	1796.8816	1796.8846	-1.66

ECC (with sample chromatogram)

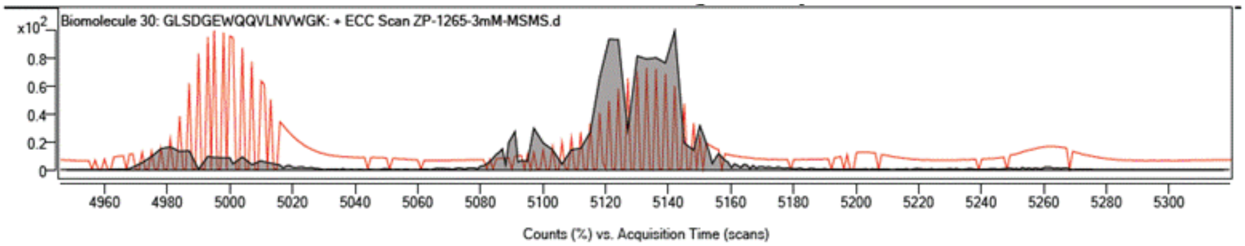

Mass Spectrum (with MFE spectrum, if available)

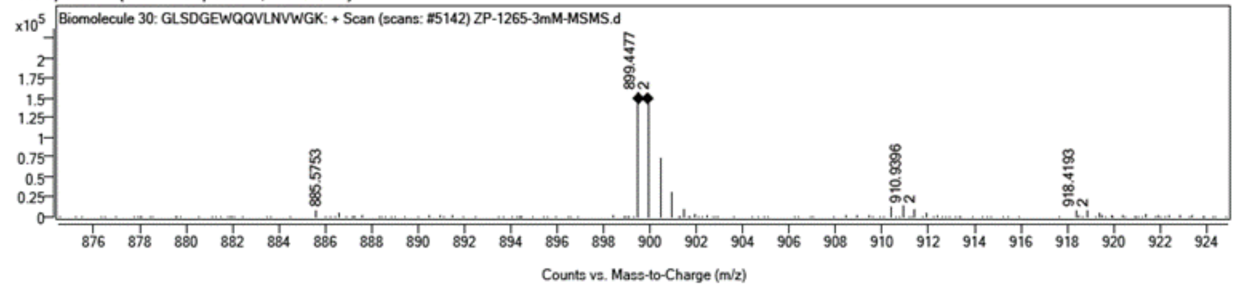

Fragment Spectra (if available)

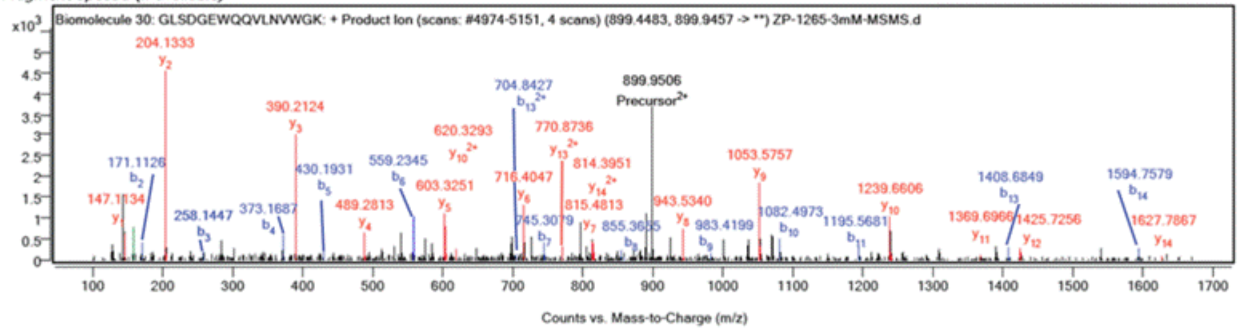

m/z	Diff (ppm)	Abund	Ion	Z
147.1134	-4.30	624	y1	
204.1333	4.74	4540	y2	
390.2124	2.97	2999	y3	
489.2813	1.45	640	y4	
603.3251	-0.31	1104	y5	
716.4047	5.99	1310	y6	
815.4813	-4.78	397	y7	
943.5340	2.12	744	y8	
1053.5757	7.91	1843	y9	
1239.6606	2.21	1027	y10	
1369.6966	9.25	112	y11	
1425.7256	1.27	263	y12	
1627.7867	-0.22	97	y14	
620.3293	9.70	253	y10	
770.8736	9.37	316	y13	
814.3951	2.05	444	y14	
171.1126	1.45	411	b2	
258.1447	0.37	204	b3	
373.1687	8.36	592	b4	
430.1931	0.43	195	b5	
559.2345	2.30	1034	b6	
745.3079	9.70	416	b7	
855.3655	-2.77	230	b8	
983.4199	1.90	152	b9	
1082.4973	-6.58	512	b10	
1195.5681	5.11	313	b11	
1408.6849	0.47	303	b13	
1594.7579	4.39	269	b14	
704.8427	5.28	128	b13	
159.0901	9.94	790	W	
899.4484	1.34	2870	Precursor	
899.9506	0.73	3679	Precursor	
900.4394	14.99	746	Precursor	

Fragment Spectrum Peaks

*Identified peptide fragment **(1 site)**: VEADIAGHGQEVLIR (Sequence: AA 17-31, Q26)*

Biomol	Seq Loc	Rule	Pred Mods	RT	Height	Mass	Tgt Mass	Diff (ppm)
11	A(17-31)	Complete digest, Predicted modifications	dehydration 10	3681.000	874549	1587.8362	1587.8369	-0.46

ECC (with sample chromatogram)

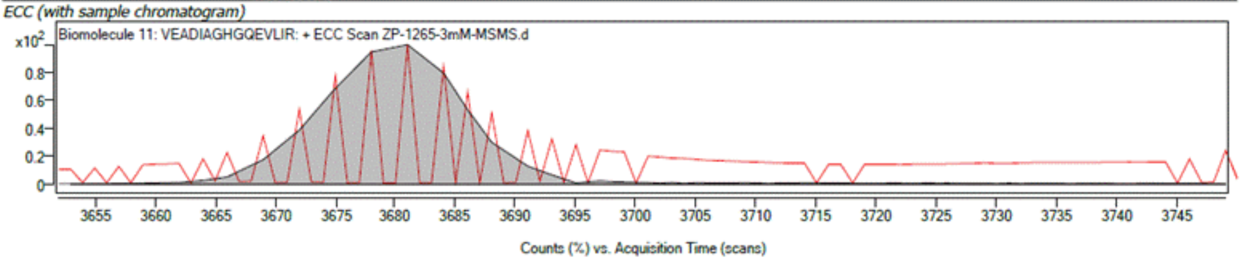

Mass Spectrum (with MFE spectrum, if available)

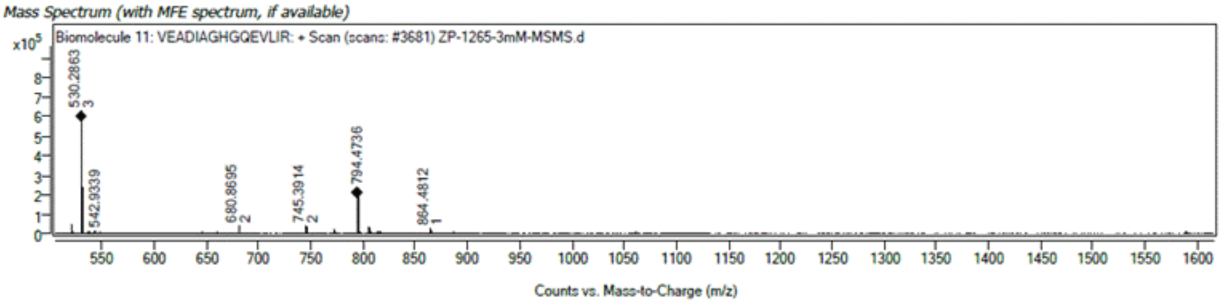

Fragment Spectra (if available)

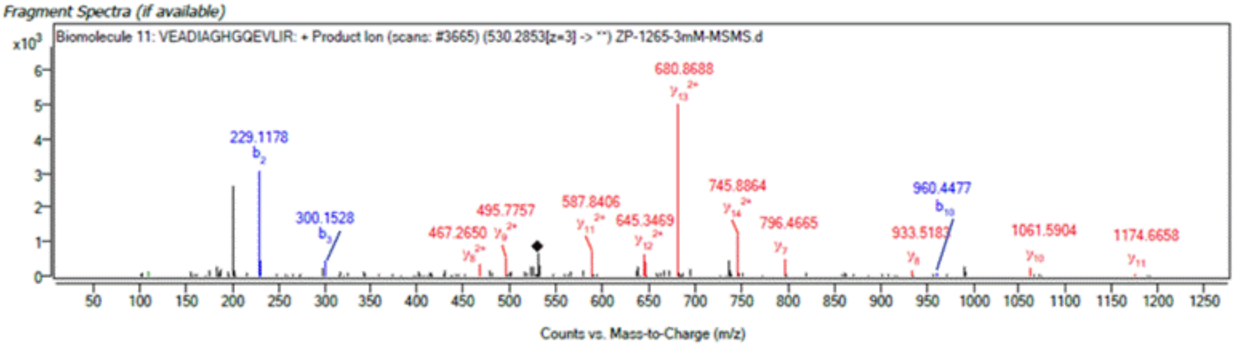

Fragment Spectrum Peaks

m/z	Diff (ppm)	Abund	Ion	Z
796.4665	1.38	484	y7	
933.5183	8.79	165	y8	
1061.5904	-5.04	233	y10	
1174.6658	2.83	55	y11	
467.2650	3.96	342	y8	
495.7757	3.76	501	y9	
587.8406	-4.13	749	y11	
645.3469	7.41	631	y12	
680.8688	2.08	5033	y13	
745.8864	9.08	1226	y14	
229.1178	2.00	3053	b2	
300.1528	8.75	430	b3	
960.4477	5.92	96	b10	
110.0707	5.04	100	H	
530.2837	4.89	664	Precursor	
530.6138	13.04	256	Precursor	
531.2881	2.92	410	Precursor	

Identified peptide fragment ***(1 site)****: HPGDFGADAQGAMTK (Sequence: AA 119-133, Q128)*

Biomol	Seq Loc	Rule	Pred Mods	RT	Height	Mass	Tgt Mass	Diff (ppm)
95	A(119-133)	Complete digest, Predicted modifications	dehydration 10	3046.000	128551	1483.6505	1483.6514	-0.60

ECC (with sample chromatogram)

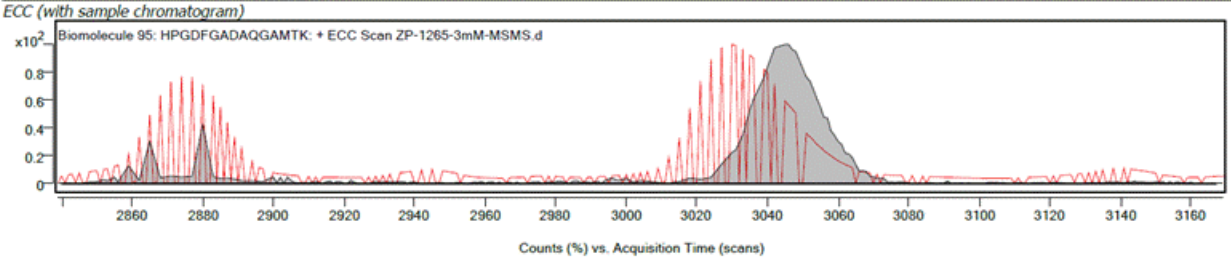

Mass Spectrum (with MFE spectrum, if available)

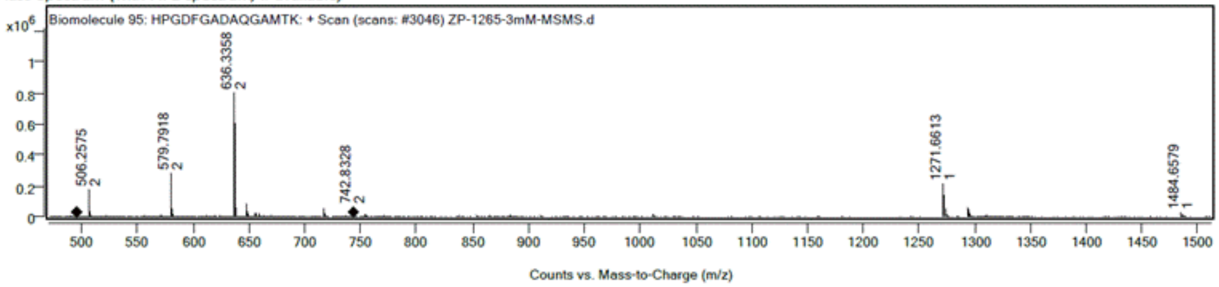

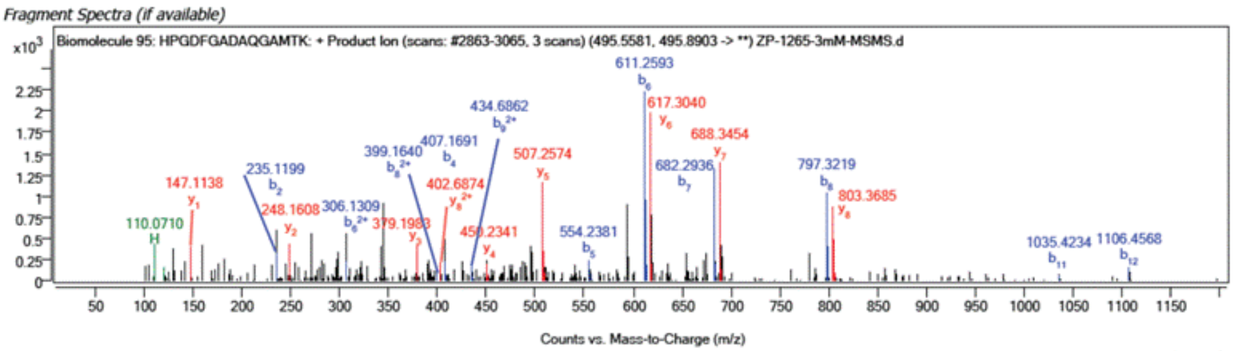

Fragment Spectrum Peaks

m/z	Diff (ppm)	Abund	Ion	Z
147.1138	-6.58	405	y1	
248.1608	-1.31	436	y2	
379.1983	6.93	426	y3	
450.2341	8.86	202	y4	
507.2574	4.23	1160	y5	
617.3040	5.76	1990	y6	
688.3454	-1.02	1397	y7	
803.3685	3.87	869	y8	
402.6874	9.16	215	y8	
235.1199	-4.17	317	b2	
407.1691	-4.17	225	b4	
554.2381	-4.16	247	b5	
611.2593	-3.42	2233	b6	
682.2936	1.13	1326	b7	
797.3219	-0.72	1035	b8	
1035.4234	4.32	55	b11	
1106.4568	7.40	157	b12	
306.1309	4.50	221	b6	
399.1640	0.61	83	b8	
434.6862	-7.80	170	b9	
110.0710	2.22	428	H	
120.0810	-1.54	163	F	

Figure S4: Activity Assay Using Modified Myoglobin

Bioactivity Assay: Oxidation of *O*-Phenylenediamine with Hydrogen Peroxide Using Modified Myoglobin

Enzymatic assay of myoglobin activity before and after labeling was checked by oxidation of *o*-phenylenediamine with hydrogen peroxide. Oxidation of *o*-phenylenediamine to 2,3-diaminophenazine was monitored at 426 nm using Agilent Cary UV-Vis Compact. Citric acid-Na_2HPO_4 buffer was prepared by mixing 0.1 M citric acid and 0.2 M Na_2HPO_4 (1:1). Homogeneous nitrile labeled myoglobin (1 mg) obtained by the above procedure (3 mM Pd entry) and unlabeled native myoglobin (1 mg) were dissolved in 1 mL of citric acid-Na_2HPO_4 buffer separately in a cuvette. To the protein samples in cuvette was added 20 µL of 0.1 M *o*-phenylenediamine and 2 µL of 1 M hydrogen peroxide in water. The reaction was fully mixed by gently vortexing of the cuvette. The cuvettes were placed in Agilent Cary UV-Vis Compact and absorbance was measured at 426 nm every 5 min for a period of 1 h. To make a blank, 20 µL of 0.1 M o-phenylenediamine and 2 µL of 1 M hydrogen peroxide were added to 1 mL of citric acid-Na_2HPO_4 buffer and absorbance was measured at 426 nm every 5 min for a period of 1 h.

Sample	Amount of protein	Concentration of H_2O_2	Conc. of o-Phenylenediamine	Total volume
Blank	0	1 mM	1 mM	1 mL
Myo-single-mod (Labeled)	1 mg	1 mM	1 mM	1 mL
Myo-unmod (Native)	1 mg	1 mM	1 mM	1 mL

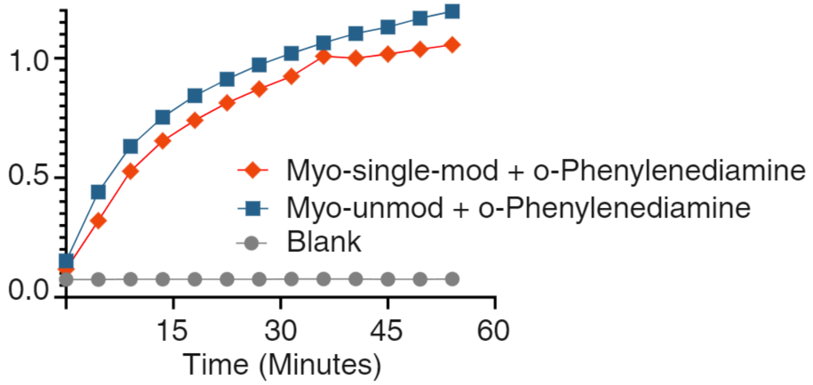

Figure S4a: Absorbance spectra of oxidation of *o*-phenylenediamine

Figure S5: Nitrile Formation Dose-Dependent Proteomics – Lysate Level

Dose-dependent nitrile dehydration of lysates and proteomics analysis. To 4 tubes (individual reactions) of 100 µg of lysate in 400 µL of 4:1 NaP buffer (10 mM, pH 7.2):CH_3CN were treated with freshly prepared acetonitrile solution of $Pd(OAc)_2$ (100 µM – 1 mM). The reaction was stirred at room temperature for 2 h. The proteins were acetone precipitated, followed by digestion using SMART Digest™ Trypsin Kit by Thermo Scientific.

LC-MS/MS.

Desalted samples were resuspended in Buffer A (0.1% FA in water) and the peptide amount was determined by Pierce™ Quantitative Peptide Assays & Standards (Thermo Fisher Scientific) according to manufacturer instructions. Samples were either transferred to LC-MS/MS vials or directly loaded onto EvoTips (EvoSep). Samples in vials were injected into a nanoElute UPLC autosampler (Bruker Daltonics) coupled to a timsTOF Pro2 mass-spectrometer (Bruker Daltonics). The peptides were loaded on a 15 cm Aurora Elite CSI column (IonOpticks) and chromatographic separation was achieved using a linear gradient starting with a flow rate of 250 nL/min from 2% Buffer B (0.1% FA in ACN) and increasing to 13% in 42 min, followed by an increase to 23% B in 65 min, 30% B in 70 min, then the flow rate was increased to 300 nL/min and 80% B in 85 min, this was kept for 5 min. For samples loaded onto EvoTips, the predefined 20 SPD Whisper Zoom method was used. For both methods, the mass-spectrometer operated in positive polarity for data

collection using a data-dependent acquisition (ddaPASEF) mode. The scan cycle consisted of one full scan followed by 10 or 7 MS/MS scans, for the nanoElute or EvoSep methods, respectively. Precursors with intensity of over 2500 (arbitrary units) were picked for fragmentation and precursors over the target value of 20,000 were dynamically excluded for 1 min. Precursors below 700 Da were isolated with a 2 Th window and ones above with 3 Th. All spectra were acquired within an m/z range of 100 to 1700 and fragmentation energy was set to 20 eV at 0.6 1/K0 and 59 eV at 1.60 1/K0.

Database search (MSFragger).

MS raw files were searched FragPipe GUI (version 20 or 22) with MSFragger (version 3.8 or 4.1) as the search algorithm. Protein identification was performed with the human Swisspot database (20′456 entries) with acetylation (N-terminus), and oxidation on methionine was set variable modification. To account for the mass shift introduced by the different chemical handles a variable mass shift of 18.0106 Da on Asparagine and Glutamine with a maximal occurrence of 3 respectively. For samples with intended deamidation, deamidation on Asparagine and Glutamine was included as further variable modification. Carbamidomethylation of cysteine residues was considered a fixed modification. Trypsin was set as the enzyme with up to two missed cleavages. The peptide length was set to 7–50, and the peptide mass range of 500–5000 Da. For MS2-based experiments, the precursor tolerance was set to 20 ppm and fragment tolerance to 20 ppm. Peptide spectrum matches (PSMs) were adjusted to a 1% false discovery rate using Percolator. For label-free quantification, match-between-runs were enabled. All downstream analysis was performed in R. Individual samples were normalized to the mean of all quantified peptides.

Data S1: Excel sheet of analysis and raw data files are attached as a supplementary document (excel-lysate-analysis)

Figure S5a: Mass Change Specificity towards Asn/Gln:

We conducted a closed search for -18.0106 Da on Ser/Thr/Asp/Glu (maximum of 2 occurances). We did observe this mass change on those residues inherent in proteomic samples. However, we observe a stark increase in modification on N/Q as Pd concentration is increased from 100 µM to 1000 µM, while signal for S/T/D/E does not significantly change

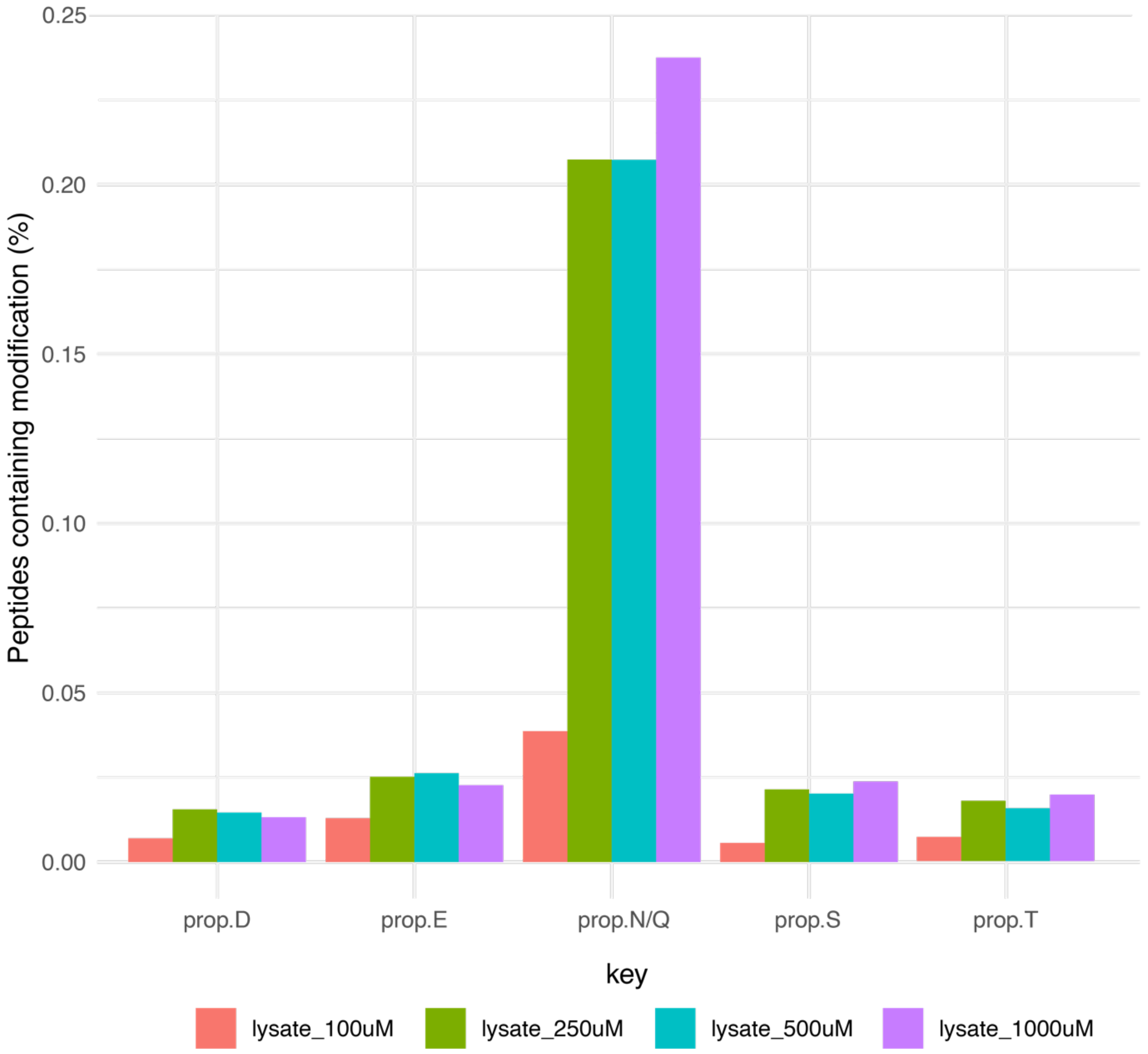

Figure S5b: Mass Change Search Histogram

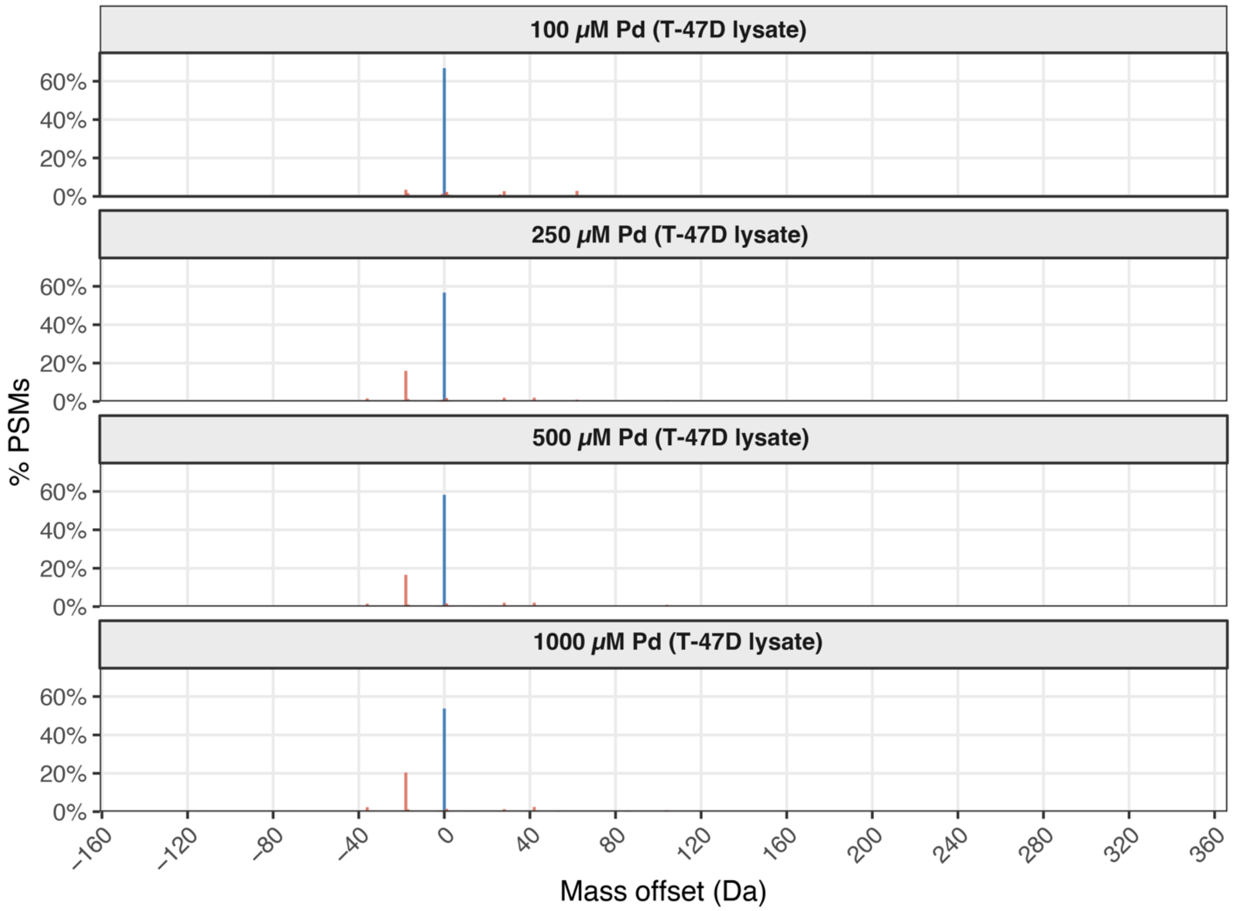

Figure S5c: Representative y and b ion spectra:

Peptides containing 1 modified Asn or Gln

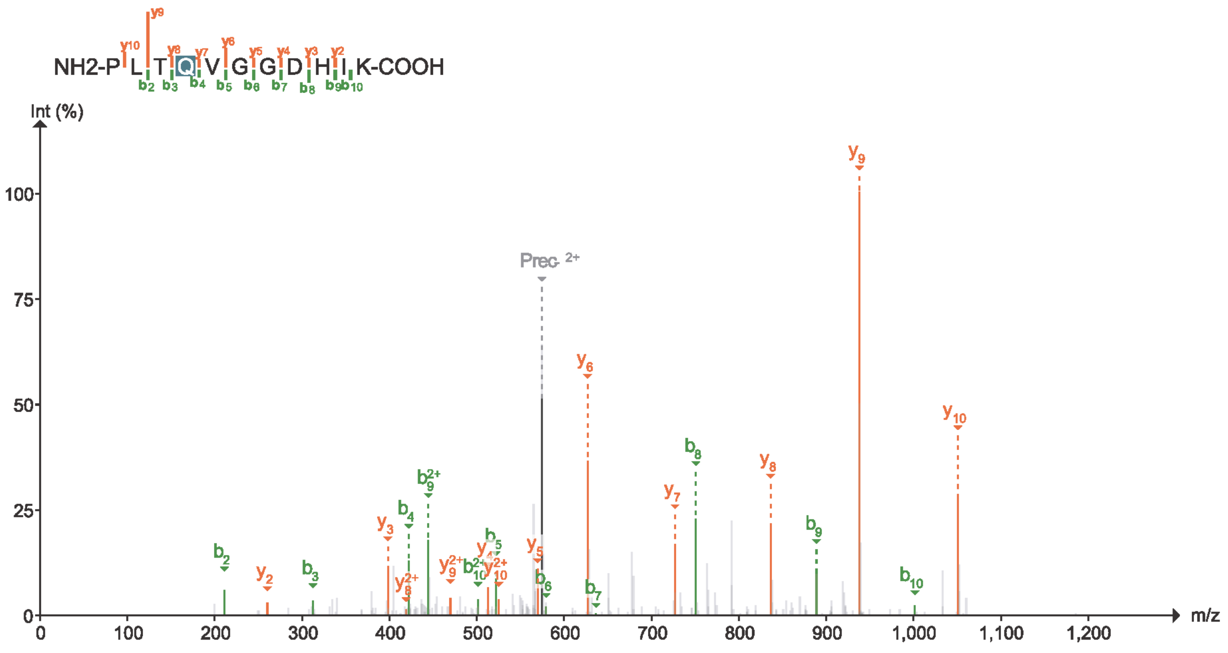

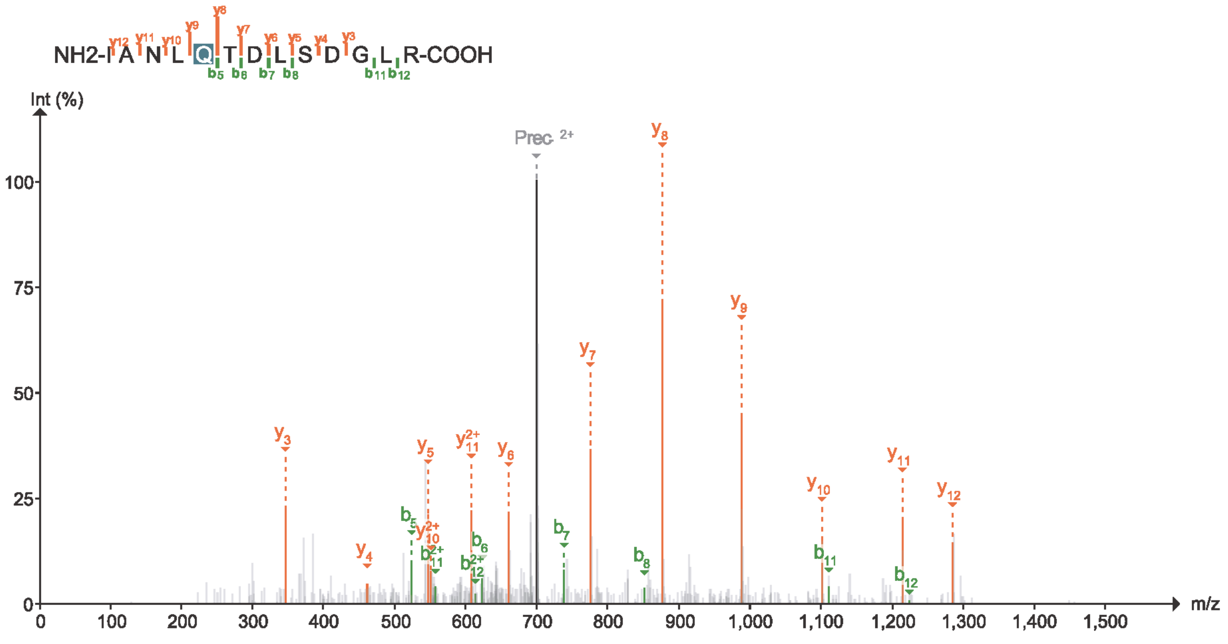

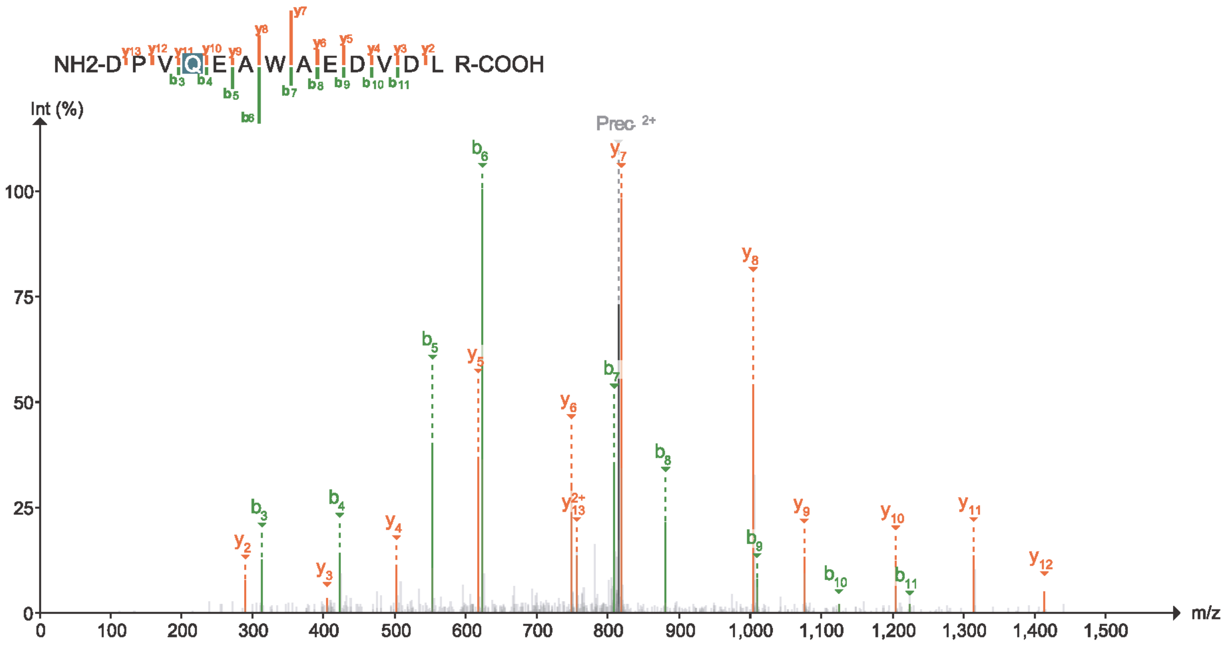
NH2-DPVQEAWAEDVDL R-COOH
Int (%)
Prec 2+
m/z

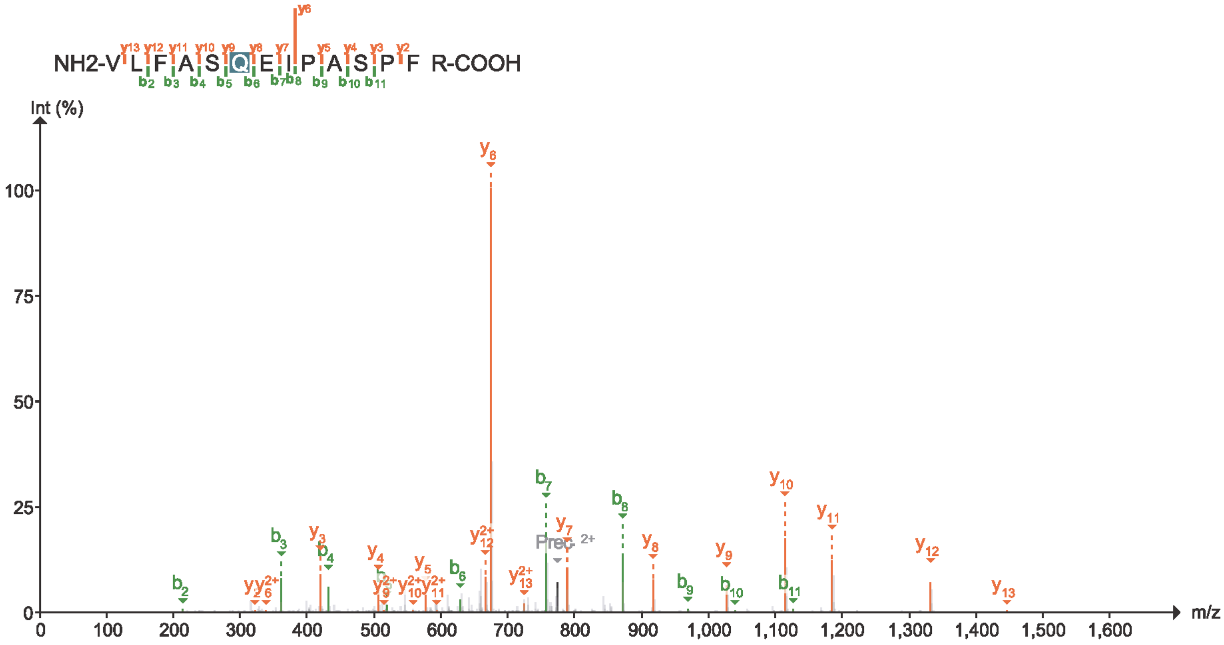
NH2-VLFASQEIPASPF R-COOH
Int (%)
Prec 2+
m/z

Peptides containing two Asn/Gln modifications

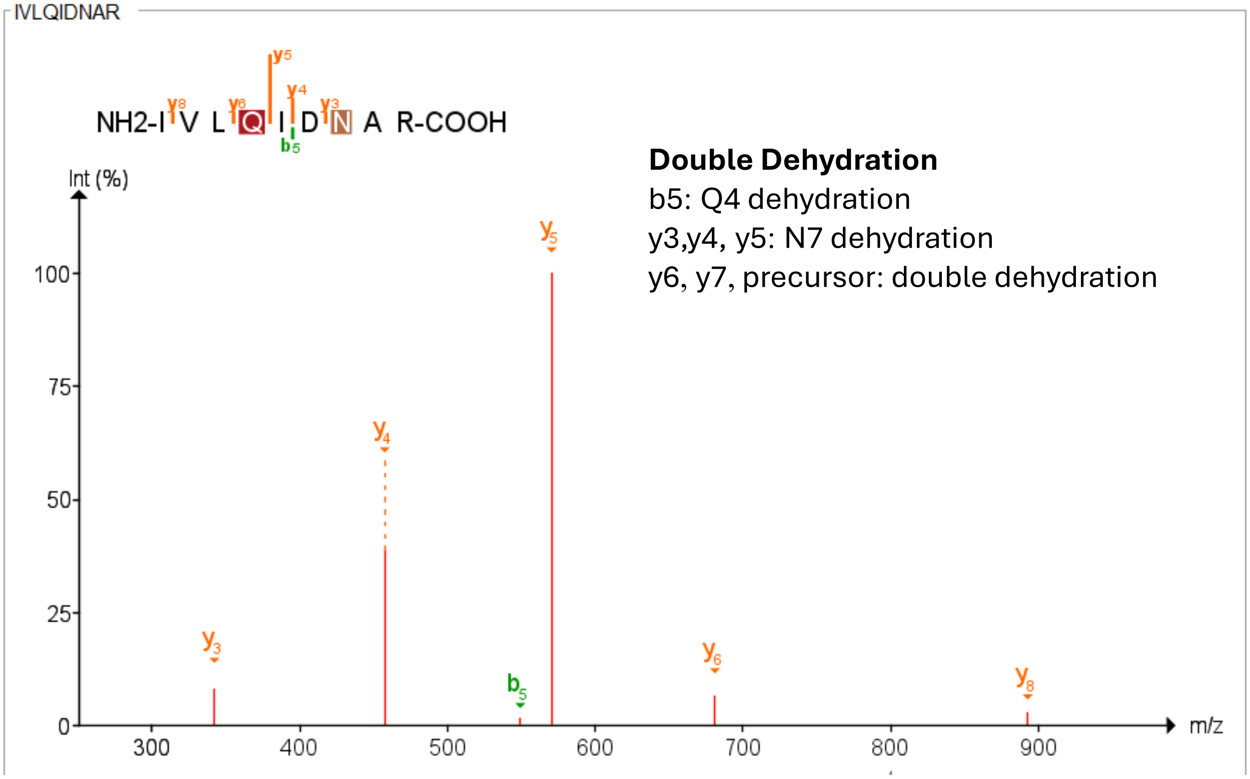

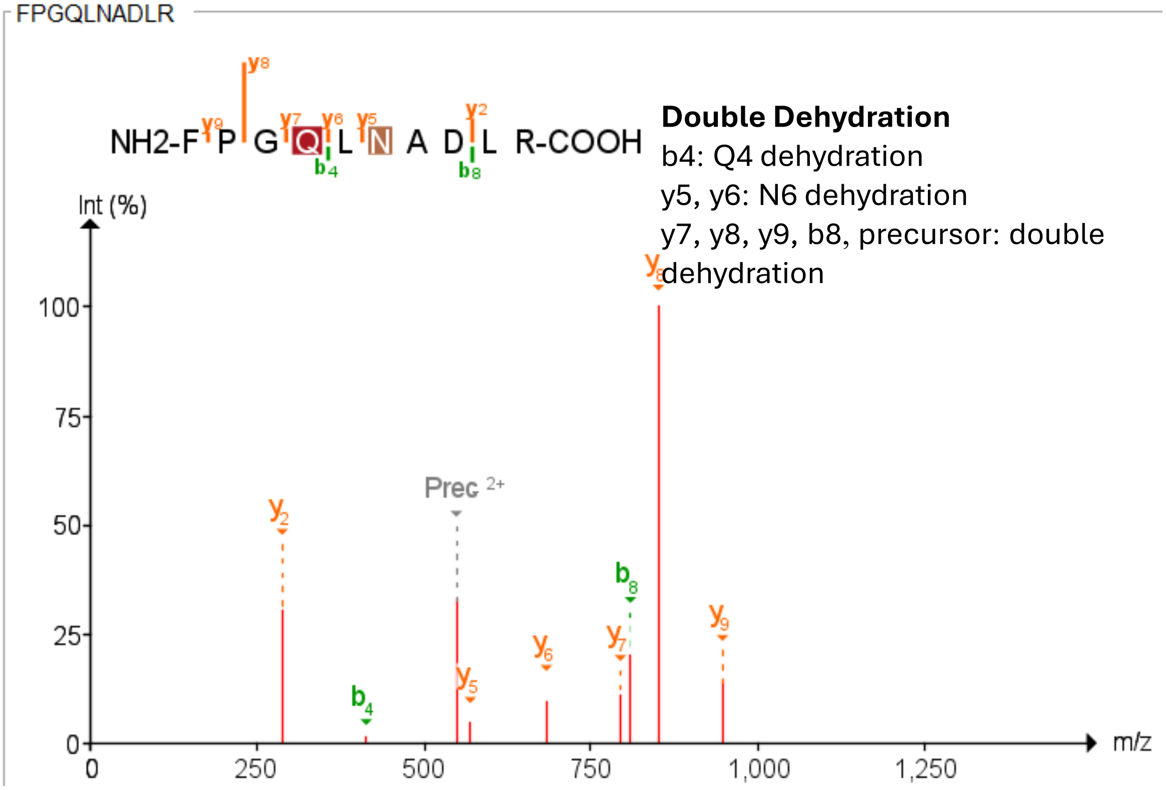

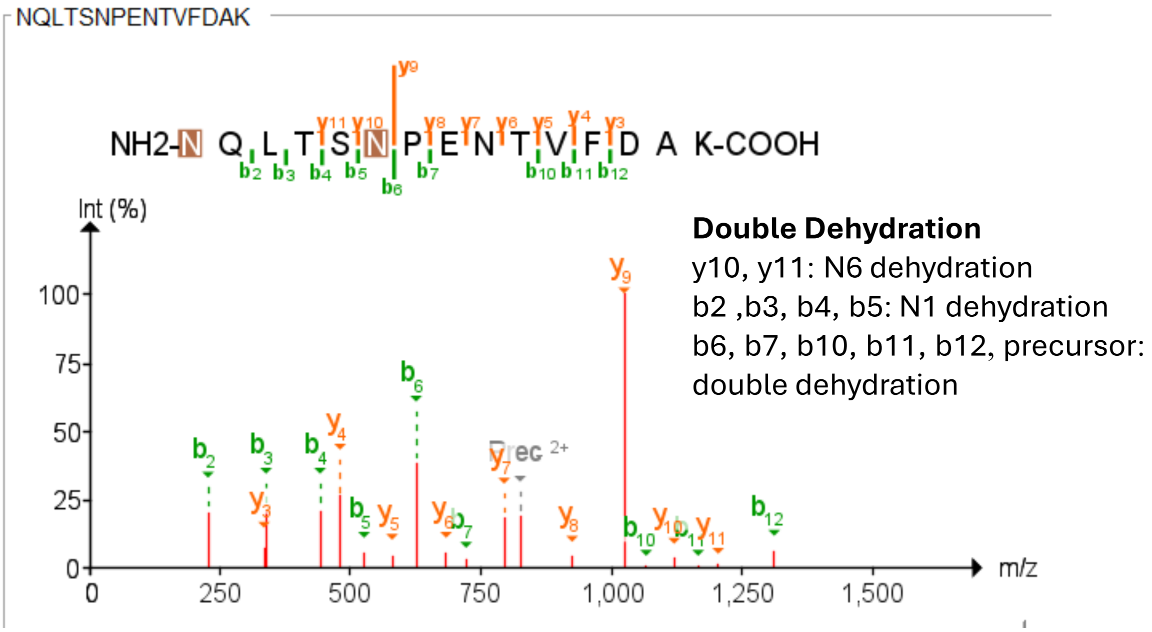

Summary of results:

Unique peptides: Excel list containing all quantified peptides were imported into RStudio and data frame of quantification columns (Intensity, Max LFQ intensity, matchtype) for each concentration extracted. Peptides in each concentration (100 µM – 1 mM) were sorted and filtered based on Max LFQ intensity (>0) and Match type of "MSMS" or Match Between Runs "MBR". Peptides found via MBR are separated for clarity. Duplicate entries were removed to obtain unique proteins for each concentration. List of proteins are found in:

Excel-lysate-analysis (sheet 1, 0 µM)

Excel-lysate-analysis (sheet 2, 100 µM)

Excel-lysate-analysis (sheet 3, 250 µM)

Excel-lysate-analysis (sheet 4, 500 µM)

Excel-lysate-analysis (sheet 5, 1000 µM)

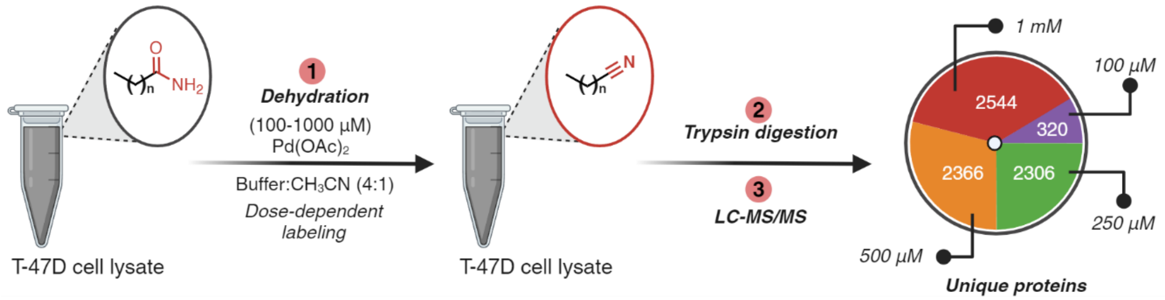

Figure S5d: Hyper-reactive Asn/Gln sites: To identify hyper-reactive Asn/Gln sites in human proteome, Excel list containing all quantified peptides were imported into RStudio and data frame of quantification columns (Intensity, Max LFQ intensity, match type) for each concentration extracted. Peptides identified in all 4 concentrations (100 µM – 1 mM) were extracted, followed by filtration based on Max LFQ intensity (>0) and Match type of "MSMS" across all 4 concentrations. MBR matched peptides were not included for this analysis. Duplicate entries were removed to obtain unique 445 hyper-reactive Asn/Gln sites. List of peptides are found in:

Excel-lysate-analysis (sheet 6, hyper-reactive peptides)

Figure S5e: Sequence motif of modified Asn/Gln sites: To identify the sequence motif of modified Asn/Gln sites, Excel list containing the sequences of hyper-reactive Asn/Gln sites were utilized. Sequences containing 4 residues from the left and 4 residues from the right of modified Asn/Gln sites were utilized, with Asn or Gln as fixed positions. Sequence motif was generated using "probability logo generator for biological sequence motif" plogo v1.2.0[2]

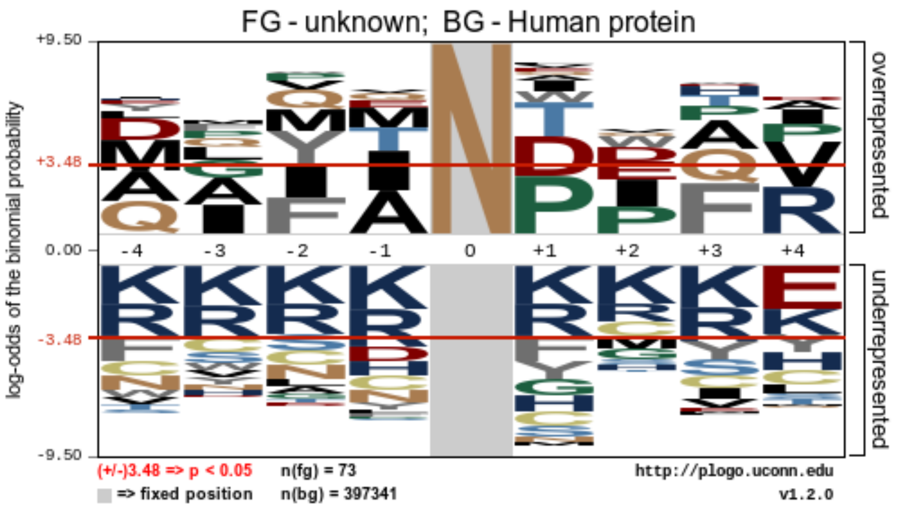

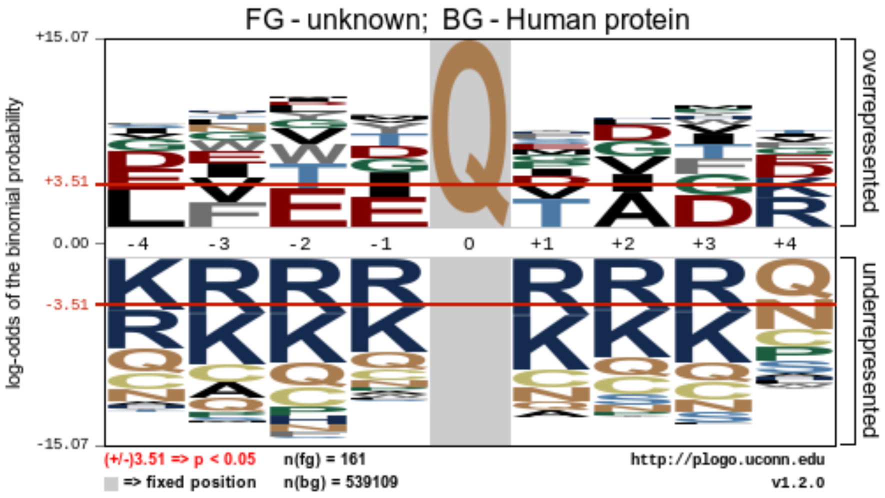

Figure S5f: Gene Ontology (GO) Analysis: To evaluate the biological processes and localization of modified proteins, Excel list containing all hyper-reactive Asn/Gln proteins were utilized. Gene ID of protein targets were extracted followed by GO analysis using ShinyGO 0.77.[3] FDR cut-off was set at 0.05%. List of genes are found in:

Excel-lysate-analysis (sheet 7, GO Gene Id)

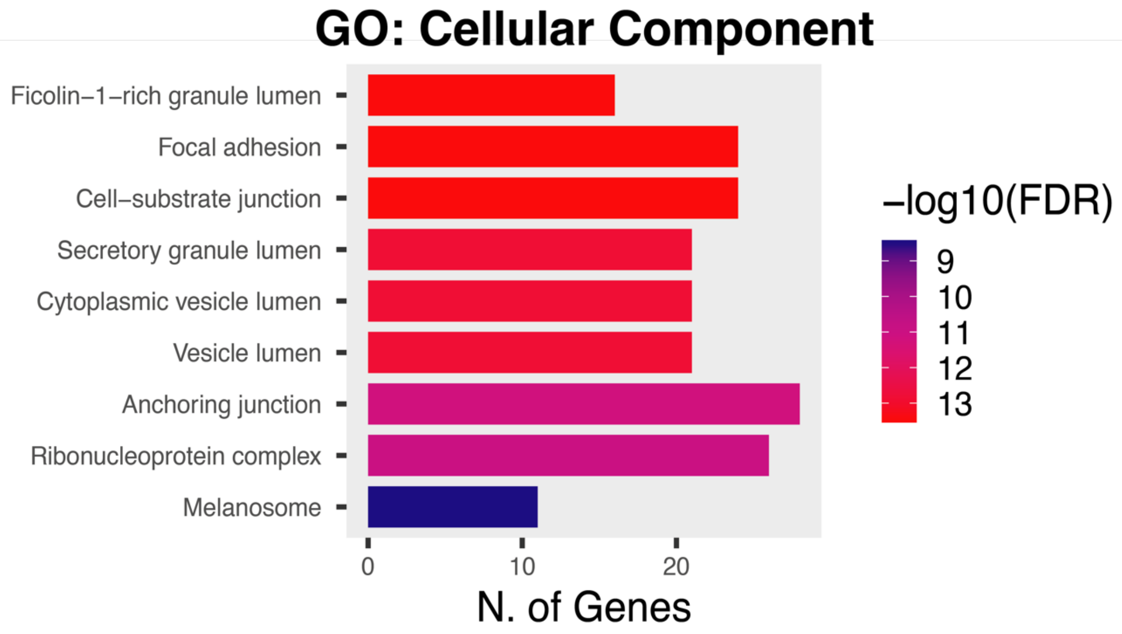

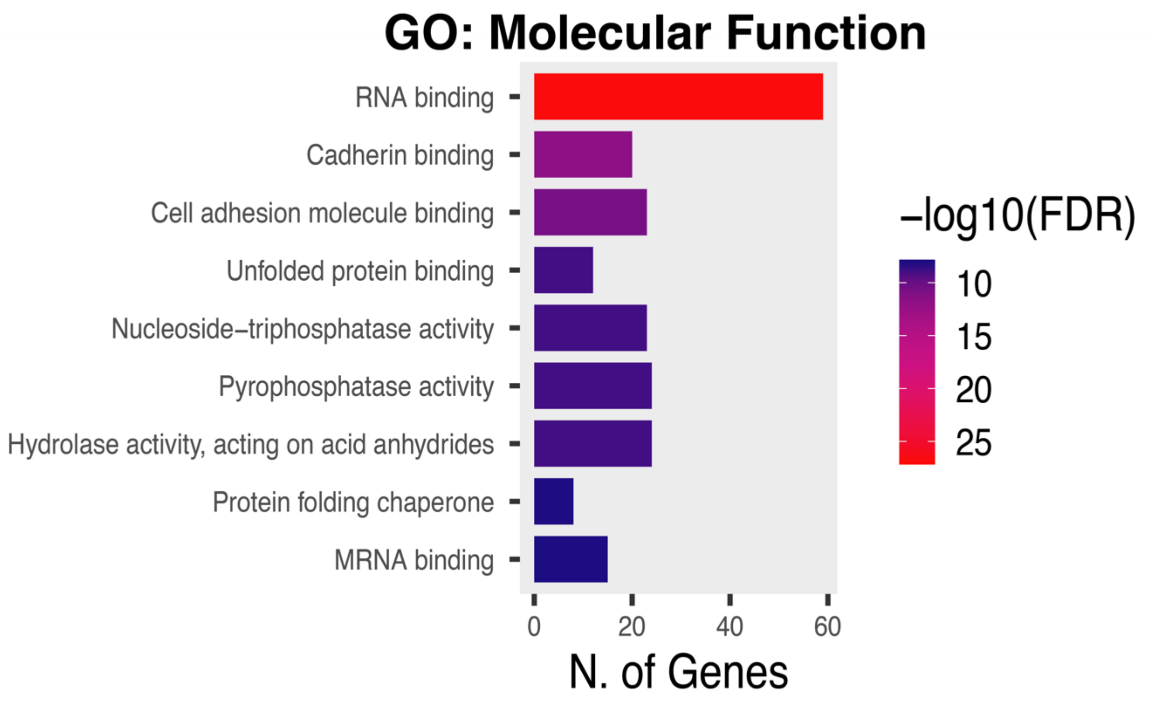

Figure S6: Cell Viability Studies

Flow cytometry analysis of cell death by Acetonitrile (ACN): T-47D cells were treated with ACN (0.1% to 2%) in media for 2 h. After incubation, cells were washed with PBS, detached with trypsin, and stained with Annexin V and PI, according to manufacturer's protocol. Annexin V (AV) conjugated to FITC was used to determine apoptosis and propidium iodide (PI) was used to determine necrosis within the cell population. Cells were analyzed via flow cytometry on a

BD FACSympony A3 cell analyzer within 1 hour to quantify cell death. A B515/20 laser was used to detect AV-FITC while a B710/50 laser was used to detect PI. FlowJo software (v. 10.10.0) was used to analyze the cytometry data. Experiment was repeated in duplicate with separate cell passages. No dosages showed a significance decrease in cell viability and maintain well above the 85% cell viability threshold.

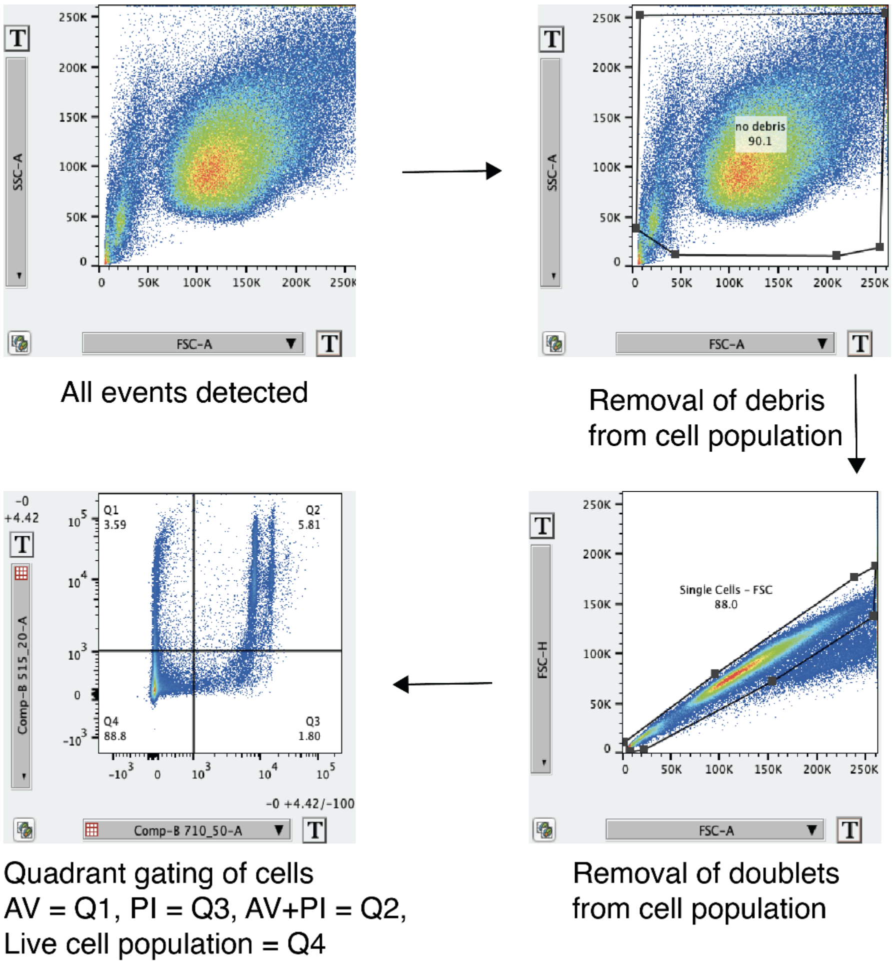

Figure S6a: Flow gating protocol

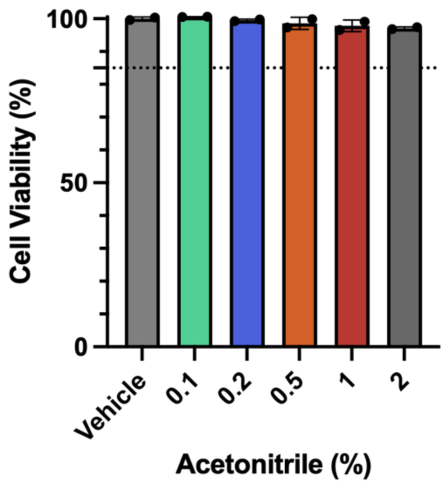

Figure S6b: Cell viability with varying ACN dosage: T-47D cells dosed with varying dosages of ACN in media for 2 h analyzed via AV/PI flow cytometry analysis. Experiment repeated in duplicate

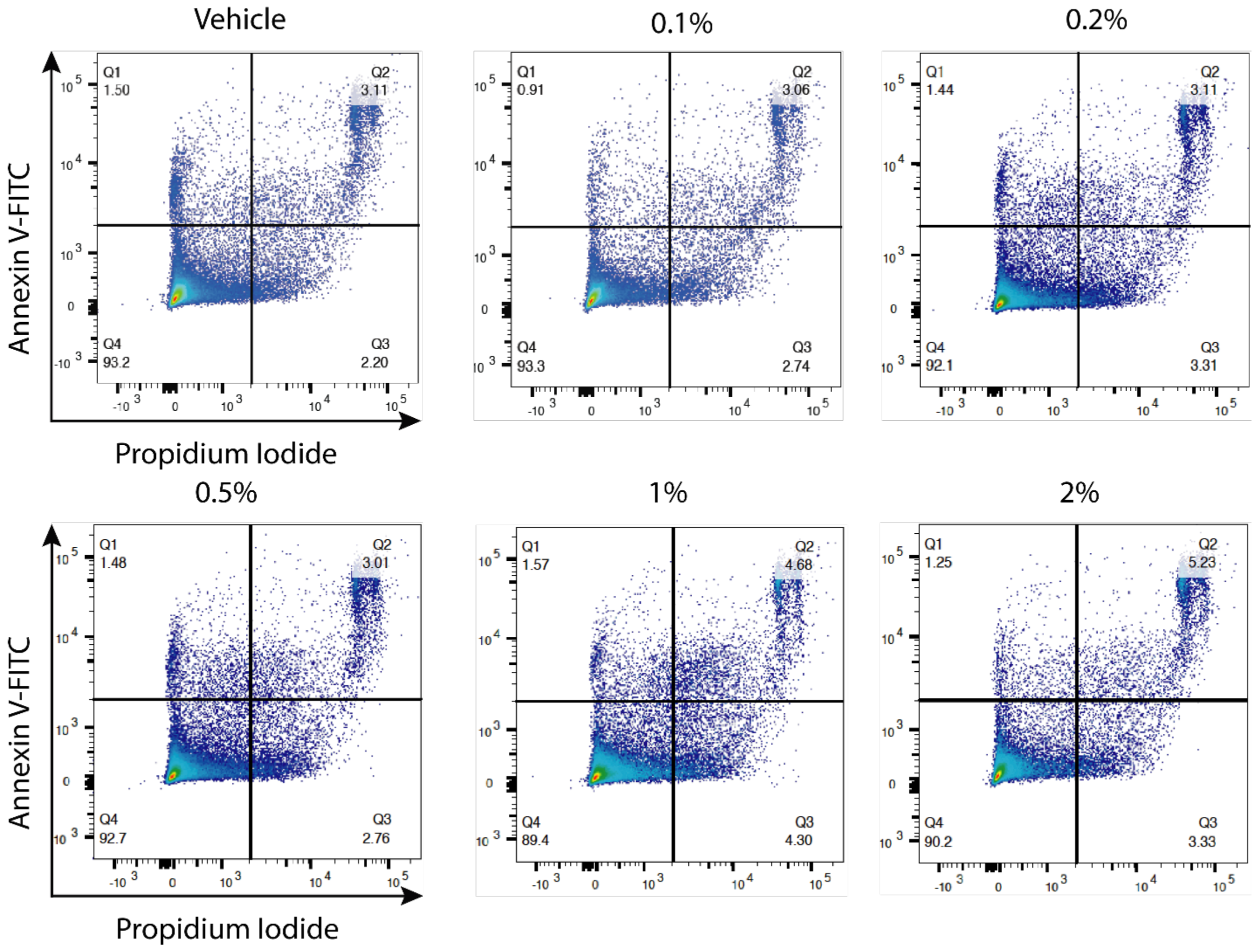

Figure S6c: Representative flow cytometry spectra

Flow cytometry analysis of cell death by $Pd(OAc)_2$: T-47D cells were treated with $Pd(OAc)_2$ (1 µM to 100 µM) in 2% ACN or vehicle (2% ACN) for 2 h. After incubation, cells were washed with PBS, detached with trypsin, and stained with Annexin V and PI, according to manufacturer's protocol. Annexin V (AV) conjugated to FITC was used to determine apoptosis and propidium iodide (PI) was used to determine necrosis within the cell population. Cells were analyzed via flow cytometry on a BD FACSympony A3 cell analyzer within 1 hour to quantify cell death. A B515/20 laser was used to detect AV-FITC while a B710/50 laser was used to detect PI. FlowJo software (v. 10.10.0) was used to analyze the cytometry data. Experiment was repeated in duplicate with separate cell passages. All doses maintained above the 85% cell viability threshold.

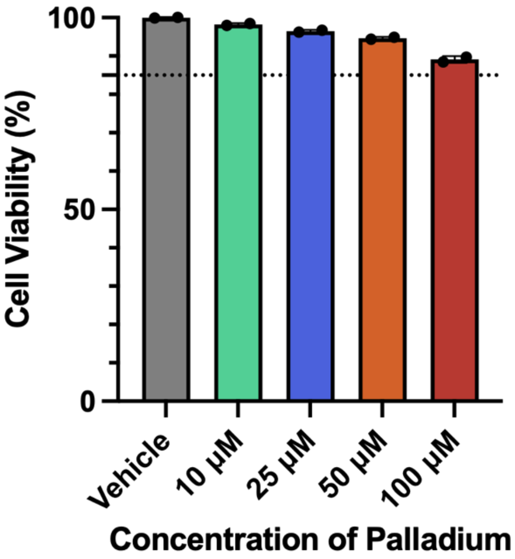

Figure S6d: Cell viability with varying Pd dosage: T-47D cells dosed with varying dosages of ACN in media for 2 h analyzed via AV/PI flow cytometry analysis. Experiment repeated in duplicate

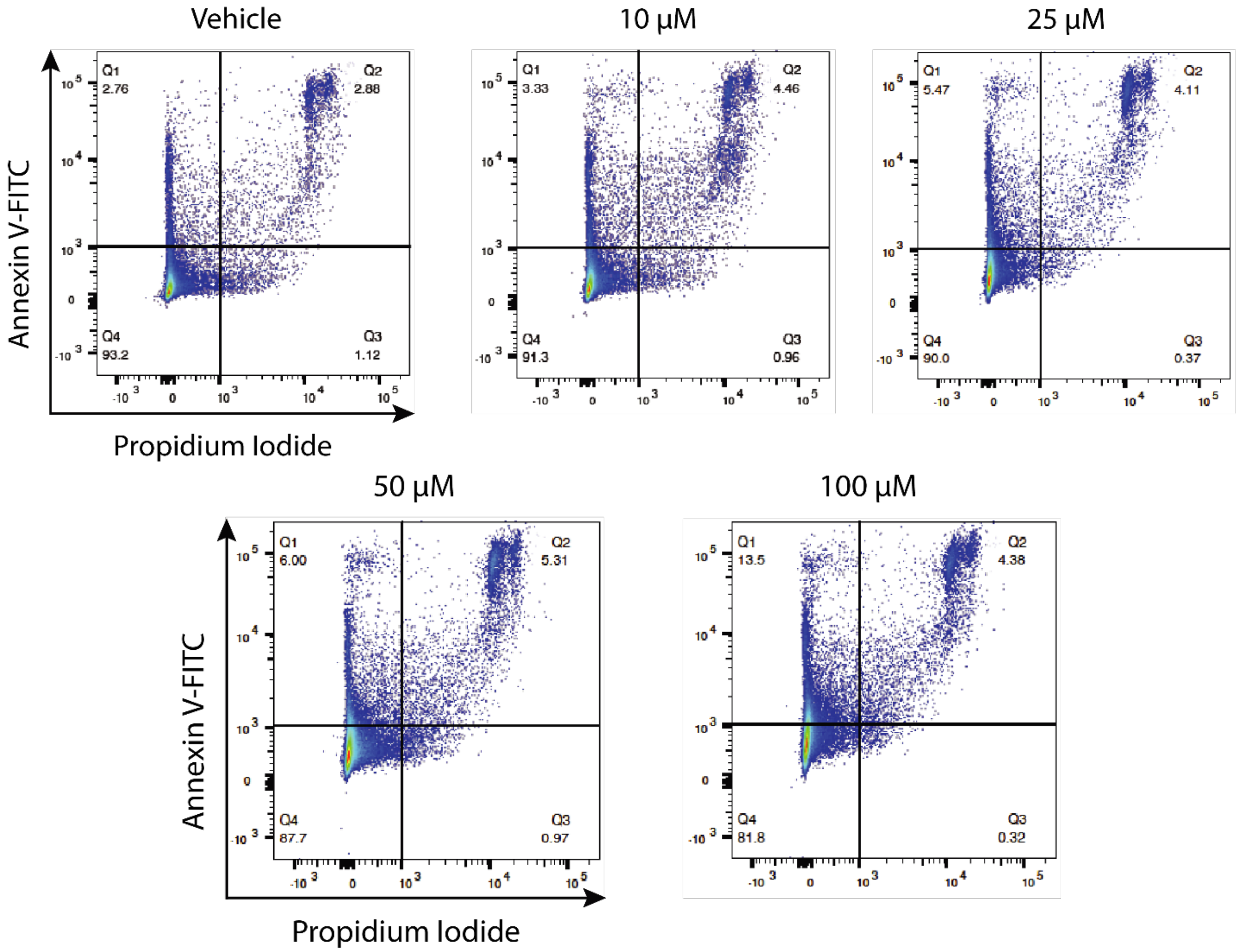

Figure S6e: Representative flow cytometry spectra

Figure S7: Nitrile Formation Dose-Dependent Proteomics – Live Cells

Dose-dependent nitrile-dehydration in live cells.

Live T-47D cells were plated on 6 cm petri dishes supplemented with RPMI 1640 media and incubated for 24 h. Cells were then treated with $Pd(OAc)_2$ (1 µM to 100 µM) and 2% acetonitrile for 2 h. After 2 h, cells were washed 3 times with cold PBS and lysed using RIPA buffer (50 mM Tris HCl [pH 8], 150 mM NaCl, 1% NP-40, 0.5% sodium deoxycholate, 0.1% SDS) supplemented with protease and phosphatase inhibitors. Lysates were centrifuged at 6,500 x g for 10 min at 4 °C, and soluble lysate was collected. 100 µg of lysates were digested using SMART Digest™ Trypsin Kit by Thermo Scientific, followed by analysis using LC-MS/MS.

Data S2: Excel sheet of analysis and raw data files are attached as a supplementary document (excel-liveCell-analysis)

Figure S7a: Representative y and b ion spectra:

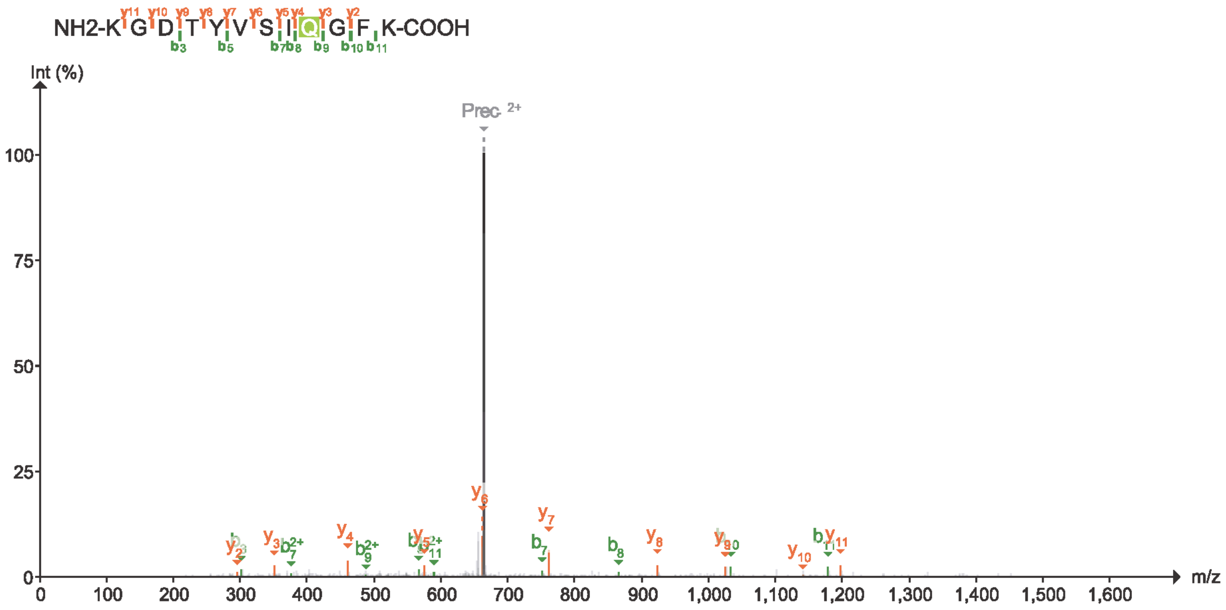

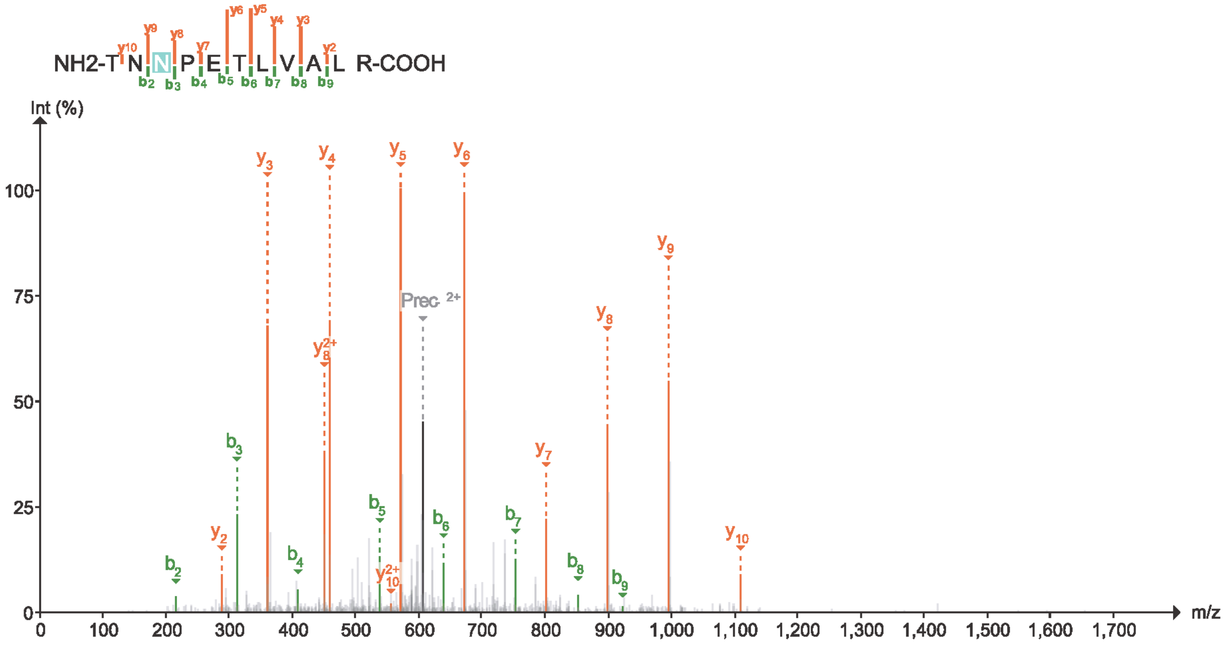

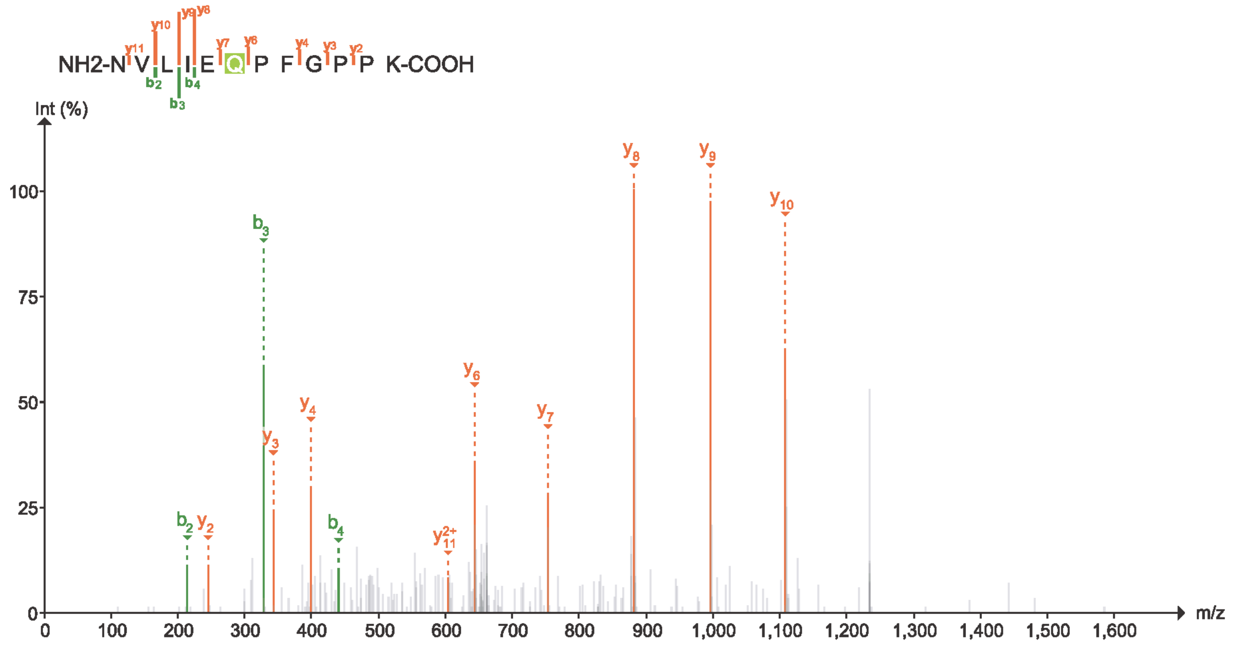

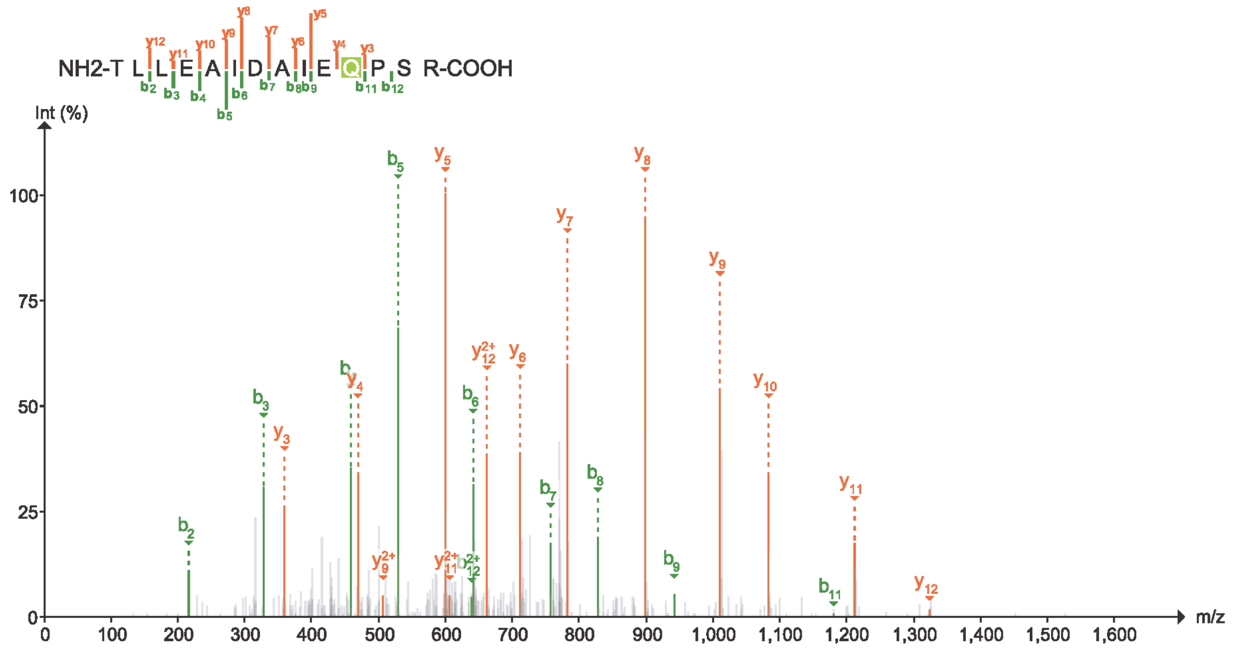

Summary of results:

Unique peptides: Excel list containing all quantified peptides were imported into RStudio and data frame of quantification columns (Intensity, Max LFQ intensity, matchtype) for each concentration extracted. Peptides in each concentration (10 µM – 100 µM) were sorted and filtered based on Max LFQ intensity (>0) and Match type of “MSMS” or Match Between Runs “MBR”.

Peptides found via MBR are separated for clarity. Duplicate entries were removed to obtain unique peptides for each concentration. List of peptides are found in:

Excel-liveCell-analysis (sheet 1, 10 μM)

Excel- liveCell -analysis (sheet 2, 25 μM)

Excel- liveCell -analysis (sheet 3, 50 μM)

Excel- liveCell -analysis (sheet 4, 100 μM)

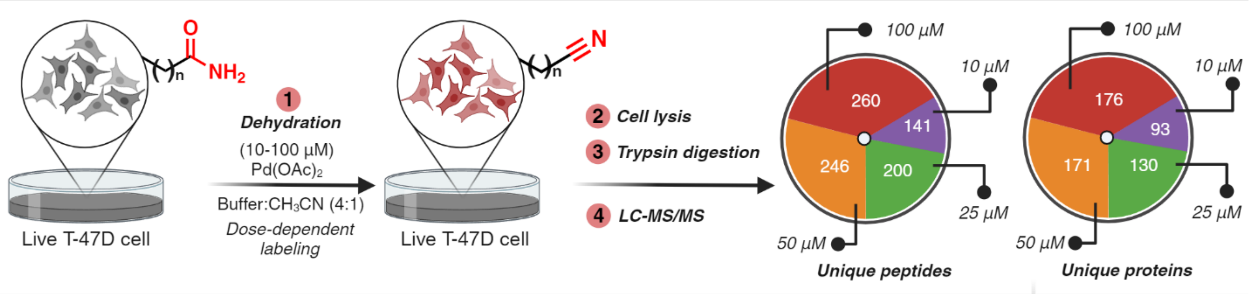

Figure S7b: Hyper-reactive Asn/Gln sites: To identify hyper-reactive Asn/Gln sites in live cell, Excel list containing all quantified peptides were imported into RStudio and data frame of quantification columns (Intensity, Max LFQ intensity, match type) for each concentration extracted. Peptides identified in all 4 concentrations (50 μM – 100 μM) were extracted, followed by filtration based on Max LFQ intensity (>0) and Match type of "MSMS" across all 4 concentrations. No MBR matched peptides were found across all 4 concentrations. Duplicate entries were removed to obtain unique 203 hyper-reactive Asn/Gln sites. List of peptides are found in:

Excel-liveCell-analysis (sheet 5, hyper-reactive peptides)

Figure S7c: Gene Ontology (GO) Analysis: To evaluate the biological processes and localization of modified proteins, Excel list containing all hyper-reactive Asn/Gln proteins were utilized. Gene ID of protein targets were extracted, followed by GO analysis using ShinyGO 0.77.[3] FDR cut-off was set at 0.05%, List of genes are found in:

Excel-liveCell-analysis (sheet 6, GO Gene Id)

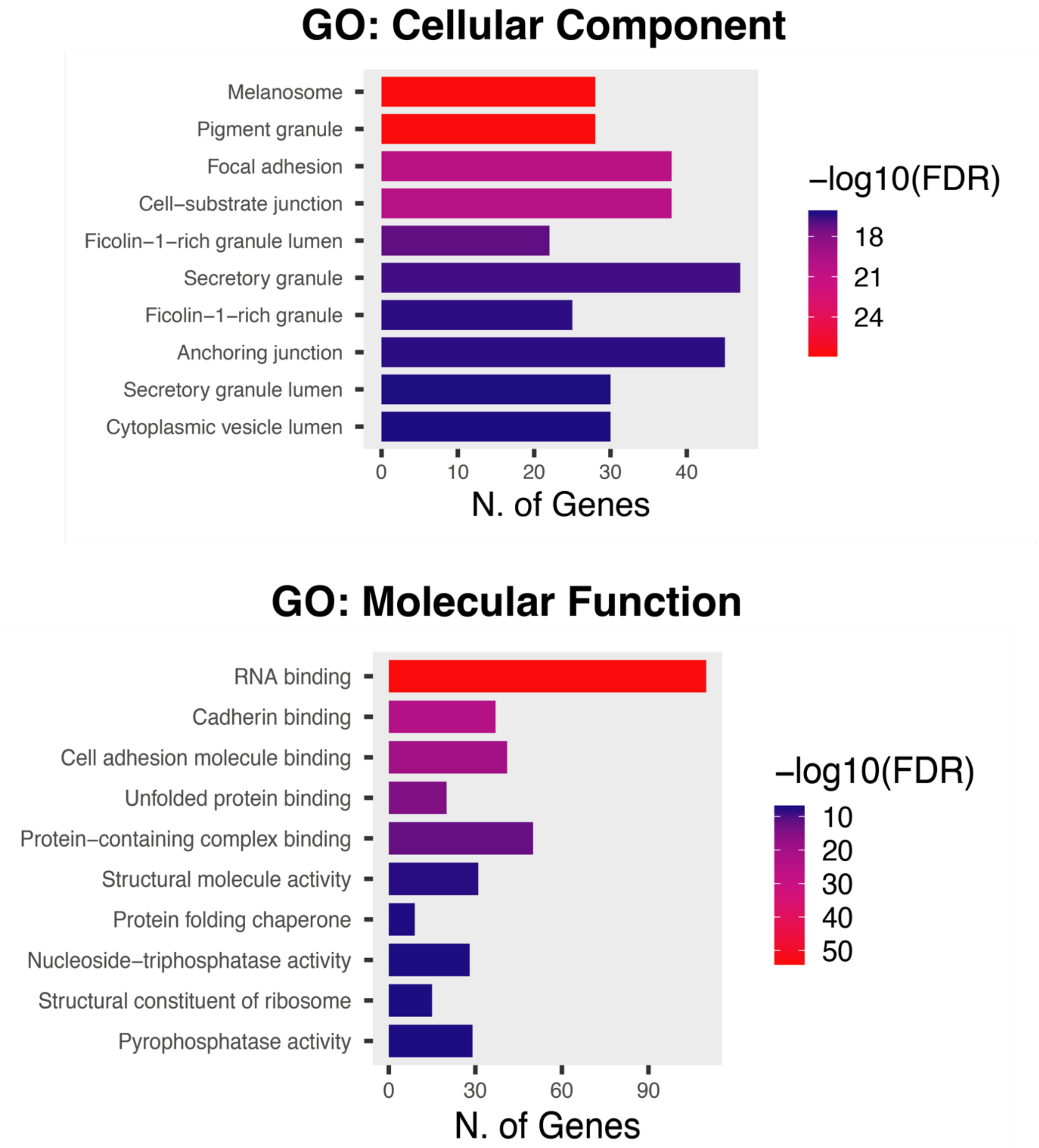

Figure S8: Nitrile Dehydration for Profiling Deamidation Post-Translational Modifications

Time-dependent incorporation of deamidation.

To 3 tubes (individual reactions) of 100 µg of lysate in 400 µL of ammonium bicarbonate buffer (50 mM, pH 8.8) were incubated at 65 °C for 0, 30, 60, or 120 min. Control sample without deamidation was generated by omitting suspension of lysates in ammonium bicarbonate buffer

and heating at 65 °C. Reaction mixtures were passed through an Amicon™ Ultra 3 kDa centrifugal filter and washed with H_2O (2×0.5 mL) to remove the small molecule impurities.

Dose-dependent dehydration reaction of lysates and proteomics analysis.

Deamidated lysates were resuspended in 400 µL of 4:1 NaP buffer (10 mM, pH 7.2):ACN and treated with freshly prepared acetonitrile solution of $Pd(OAc)_2$ (500 µM). The reaction was stirred at room temperature for 2 h. The proteins were acetone precipitated, followed by digestion using SMART Digest™ Trypsin Kit by Thermo Scientific.

LC-MS/MS.

Desalted samples were resuspended in Buffer A (0.1% FA in water) and the peptide amount was determined by Pierce™ Quantitative Peptide Assays & Standards (Thermo Fisher Scientific) according to manufacturer instructions. Samples were either transferred to LC-MS/MS vials or directly loaded onto EvoTips (EvoSep). Samples in vials were injected into a nanoElute UPLC autosampler (Bruker Daltonics) coupled to a timsTOF Pro2 mass-spectrometer (Bruker Daltonics). The peptides were loaded on a 15 cm Aurora Elite CSI column (IonOpticks) and chromatographic separation was achieved using a linear gradient starting with a flow rate of 250 nL/min from 2% Buffer B (0.1% FA in ACN) and increasing to 13% in 42 min, followed by an increase to 23% B in 65 min, 30% B in 70 min, then the flow rate was increased to 300 nL/min and 80% B in 85 min, this was kept for 5 min. For samples loaded onto EvoTips, the predefined 20 SPD Whisper Zoom method was used. For both methods, the mass-spectrometer operated in positive polarity for data collection using a data-dependent acquisition (ddaPASEF) mode. The scan cycle consisted of one full scan followed by 10 or 7 MS/MS scans, for the nanoElute or EvoSep methods, respectively. Precursors with intensity of over 2500 (arbitrary units) were picked for fragmentation and precursors over the target value of 20,000 were dynamically excluded for 1 min. Precursors below 700 Da were isolated with a 2 Th window and ones above with 3 Th. All spectra were acquired within an m/z range of 100 to 1700 and fragmentation energy was set to 20 eV at 0.6 1/K0 and 59 eV at 1.60 1/K0.

Database search (MSFragger).

MS raw files were searched FragPipe GUI (version 20 or 22) with MSFragger (version 3.8 or 4.1) as the search algorithm. Protein identification was performed with the human Swisspot database (20′456 entries) with acetylation (N-terminus), and oxidation on methionine was set variable modification. To account for the mass shift introduced by the different chemical handles a variable mass shift of 18.0106 Da on Asparagine and Glutamine with a maximal occurrence of 3 respectively. For samples with intended deamidation, deamidation on Asparagine and Glutamine was included as further variable modification. Carbamidomethylation of cysteine residues was considered a fixed modification. Trypsin was set as the enzyme with up to two missed cleavages.

The peptide length was set to 7–50, and the peptide mass range of 500–5,000 Da. For MS2-based experiments, the precursor tolerance was set to 20 ppm and fragment tolerance to 20 ppm. Peptide spectrum matches (PSMs) were adjusted to a 1% false discovery rate using Percolator. For label-free quantification, match-between-runs were enabled. All downstream analysis was performed in R. Individual samples were normalized to the mean of all quantified peptides.

Data S3: Excel sheet of analysis and raw data files are attached as a supplementary document (excel-deamidation-analysis)

Summary of results:

Unique peptides: Excel list containing all quantified peptides were imported into RStudio and data frame of quantification columns (Intensity, Max LFQ intensity, matchtype) for each concentration extracted. Peptides in each deamidation time point (0, 30, 60, and 120 min) were sorted and filtered based on intensity (>0) and Match type of "MSMS". Duplicate entries were removed to obtain unique peptides for each concentration. List of peptides are found in:

Excel-deamidation-analysis (sheet 1, 0 minute)

Excel- deamidation -analysis (sheet 2, 30 min)

Excel- deamidation -analysis (sheet 3, 60 min)

Excel- deamidation -analysis (sheet 4, 120 min)

Excel – site localization (sheet 5): Note, this was run with MSFragger v4.4 leading to minor difference in found peptides compared to sheets 1-4.

Sequence motif of modified Asn/Gln sites: To identify the sequence motif of deamidated Asn sites, Excel list containing the sequences of nitrile modified Asn sites not observed after 30 min of deamidation were utilized. Sequences containing 4 residues from the left and 4 residues from the right of modified Asn sites were utilized, with Asn as fixed positions. Sequence motif was generated using "probability logo generator for biological sequence motif" plogo v1.2.0.[2]

Figure S9: Nitrile Dehydration for Profiling N-Glycosylation Post-Translational Modification

General cell culturing and drug treatment.

Culture of *S. cerevisiae* (strain BY4741) was grown in YPD media at 30 °C overnight to saturation. The day cultures were prepared in YPD at an initial OD600 of 0.25 for approximately 4 h at 30 °C to reach mid-log phase. Cells were then collected and transferred into their respective media. *S.*

cerevisiae samples were grown in either YPD or YPD with 2 ug/mL of Tunicamycin at 30 °C. Samples were inoculated at an OD600 of 0.4 except *S. cerevisiae* grown in YPD without Tunicamycin which was inoculated at an OD600 of 0.3 due to the faster doubling time. After 4 h, samples were harvested and washed with ddH_2O. The cells were resuspended in 1 mL of RIPA Buffer and transferred to 2 mL screw-top tubes with O-rings with 200 µL of 0.1 mm silica beads. Samples were then lysed using a bead mill at 5 m/s for 5 min. The lysate was then spun down at max speed for 5 min at 4 °C. The supernatant was collected for analysis.

Dose-dependent dehydration reaction of lysates and proteomics analysis.

Prepared lysates were resuspended in 400 µL of 4:1 NaP buffer (10 mM, pH 7.2):CH_3CN and treated with freshly prepared acetonitrile solution of $Pd(OAc)_2$ (500 µM). The reaction was stirred at room temperature for 2 h. The proteins were acetone precipitated, followed by digestion using SMART Digest™ Trypsin Kit by Thermo Scientific.

LC-MS/MS.

Desalted samples were resuspended in Buffer A (0.1% FA in water) and the peptide amount was determined by Pierce™ Quantitative Peptide Assays & Standards (Thermo Fisher Scientific) according to manufacturer instructions. Samples were either transferred to LC-MS/MS vials or directly loaded onto EvoTips (EvoSep). Samples in vials were injected into a nanoElute UPLC autosampler (Bruker Daltonics) coupled to a timsTOF Pro2 mass-spectrometer (Bruker Daltonics). The peptides were loaded on a 15 cm Aurora Elite CSI column (IonOpticks) and chromatographic separation was achieved using a linear gradient starting with a flow rate of 250 nL/min from 2% Buffer B (0.1% FA in ACN) and increasing to 13% in 42 min, followed by an increase to 23% B in 65 min, 30% B in 70 min, then the flow rate was increased to 300 nL/min and 80% B in 85 min, this was kept for 5 min. For samples loaded onto EvoTips, the predefined 20 SPD Whisper Zoom method was used. For both methods, the mass-spectrometer operated in positive polarity for data collection using a data-dependent acquisition (ddaPASEF) mode. The scan cycle consisted of one full scan followed by 10 or 7 MS/MS scans, for the nanoElute or EvoSep methods, respectively. Precursors with intensity of over 2500 (arbitrary units) were picked for fragmentation and precursors over the target value of 20,000 were dynamically excluded for 1 min. Precursors below 700 Da were isolated with a 2 Th window and ones above with 3 Th. All spectra were acquired within an m/z range of 100 to 1700 and fragmentation energy was set to 20 eV at 0.6 1/K0 and 59 eV at 1.60 1/K0.

Database search (MSFragger).

MS raw files were searched FragPipe GUI (version 20 or 22) with MSFragger (version 3.8 or 4.1) as the search algorithm. Protein identification was performed with the human Swisspot database (20′456 entries) with acetylation (N-terminus), and oxidation on methionine was set variable

modification. To account for the mass shift introduced by the different chemical handles a variable mass shift of 18.0106 Da on Asparagine and Glutamine with a maximal occurrence of 3 respectively. For samples with intended deamidation, deamidation on Asparagine and Glutamine was included as further variable modification. Carbamidomethylation of cysteine residues was considered a fixed modification. Trypsin was set as the enzyme with up to two missed cleavages. The peptide length was set to 7–50, and the peptide mass range of 500–5,000 Da. For MS2-based experiments, the precursor tolerance was set to 20 ppm and fragment tolerance to 20 ppm. Peptide spectrum matches (PSMs) were adjusted to a 1% false discovery rate using Percolator. For label-free quantification, match-between-runs were enabled. All downstream analysis was performed in R. Individual samples were normalized to the mean of all quantified peptides.

Data S4: Excel sheet of analysis and raw data files are attached as a supplementary document (excel-glycosylation-tunicamycin-analysis)

Summary of results:

Unique peptides: Excel list containing all quantified peptides were imported into RStudio and data frame of quantification columns (Intensity, Max LFQ intensity, matchtype) for each concentration extracted. Peptides in each deamidation time point (0, 30, 60, and 120 min) were sorted and filtered based on intensity (>0) and Match type of "MSMS". Duplicate entries were removed to obtain unique peptides for each concentration. List of peptides are found in:

Excel-glycosylation-tunicamycin-analysis (sheet 1, No Tunicamycin both reps and no_duplicate)

Excel-glycosylation-tunicamycin-analysis (sheet 2, With Tunicamycin both reps and no_duplicate)

Excel – site localization (sheet 3): Note, this was run with MSFragger v4.4 leading to minor difference in found peptides compared to sheets 1-2.

Figure S9a: Gene Ontology (GO) Analysis: To evaluate the Biological process (BP), Molecular Function (MF), and Cellular Compartment (CC) of modified proteins, Excel list containing glycosylated Asn and Gln sites were utilized. Gene ID of protein targets were extracted followed by GO analysis using ShinyGO 0.77.[3] FDR cut-off was set at 0.05%.

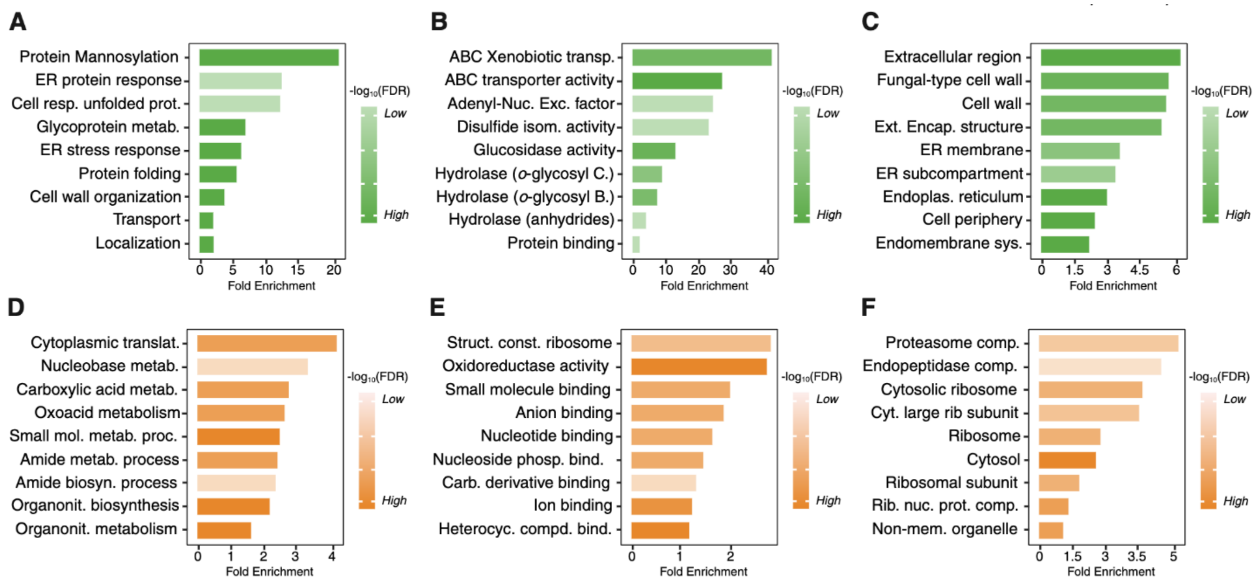

Dehydration reaction mediated chemoproteomics profiling of asparagine N-glycosylation. (A) GO-biological process analysis of previously reported glycoproteins observed in TM-treated sample. **(B)** GO-molecular function analysis of previously reported glycoproteins observed in TM-treated sample. **(C)** GO-cellular compartment analysis of previously reported glycoproteins observed in TM-treated sample. Gene list of glycoproteins was utilized as input in ShinyGO, with input and analysis species set to *S. cerevisiae.* All genes in the *S. cerevisiae* genome were used as the enrichment background. Terms with a p-value < 0.01, a minimum count of 3, and an enrichment factor > 1.5 were utilized. p-values were calculated based on the cumulative hypergeometric distribution, and q-values are calculated using the Benjamini-Hochberg procedure. **(D)** GO-biological process analysis of novel glycoproteins observed in TM-treated sample. **(E)** GO-molecular function analysis of novel glycoproteins observed in TM-treated sample. **(F)** GO-cellular compartment analysis of novel glycoproteins observed in TM-treated sample. Gene list of down-regulated proteins was utilized as input in ShinyGO, with input and analysis species set to *S. cerevisiae.* All genes in the *S. cerevisiae* genome were used as the enrichment background. Terms with a p-value < 0.01, a minimum count of 3, and an enrichment factor > 1.5 were utilized. p-values were calculated based on the cumulative hypergeometric distribution, and q-values are calculated using the Benjamini-Hochberg procedure.

Biological process analysis (A) of nitrile modified glycoproteins showed involvement in protein mannosylation, endoplasmic reticulum (ER) protein response, and the response to unfolded proteins, indicating a robust cellular adaptation to impaired glycosylation. This observation is consistent with the role of tunicamycin in blocking N-linked glycosylation, thereby triggering the unfolded protein response (UPR) pathway.[4,5] Molecular functions analysis (B) showed the notable enrichment of proteins associated with ABC transporter activity, adenyl nucleotide exchange factor activity, and various hydrolase activities, suggesting a cellular effort of TM-treated cells to manage xenobiotic stress and maintain homeostasis.[6] Lastly, cellular component analysis (C)

highlights significant enrichment in proteins localized to the extracellular region, cell wall, and ER membrane, emphasizing the widespread impact of tunicamycin on membrane-associated and secretory pathways.

Interestingly, GO analysis of novel glycoproteins identified by the dehydration platform further highlights the impact of tunicamycin treatment on various other biological processes, molecular functions, and cellular components in *S. cerevisiae* (D, E, and F). Biological processes analysis (D) revealed a marked enrichment of proteins involved in cytoplasmic translation, nucleobase and carboxylic acid metabolism, and amide biosynthesis, suggesting a shift in cellular metabolism and protein synthesis activities. Molecular function analysis (E) showed a significant enrichment in structural constituents of the ribosome, oxidoreductase activity, and various binding activities (small molecule, anion, and nucleotide binding), reflecting a heightened need for protein synthesis machinery and redox homeostasis under stress conditions. Furthermore, the cellular component analysis (F) revealed a notable enrichment of proteins associated with the proteasome complex, endopeptidase complex, and cytosolic ribosome, indicating an increased turnover of misfolded proteins and a reliance on the proteasomal degradation pathway.

Figure S10: Nitrile Dehydration for Profiling N-Glycosylation in Pathogenic and Non-Pathogenic Yeast

General cell culturing and drug treatment.

Culture of *C. albicans* (strain SC5314) was grown in YPD media at 30 °C overnight to saturation. The day cultures were prepared in YPD at an initial OD600 of 0.25 for approximately 4 h at 30 °C to reach mid-log phase. Cells were then collected and transferred into their respective media. The yeast form of *C. albicans* samples were grown in RPMI 1640 + 10% ddH_2O at 30 °C and the hyphae form of *C. albicans* was grown in RPMI 1640 + 10% FBS at 37 °C. After 4 h, samples were harvested and washed with ddH_2O. The cells were resuspended in 1 mL of RIPA Buffer and transferred to 2 mL screw-top tubes with O-rings with 200 µL of 0.1 mm silica beads. Samples were then lysed using a bead mill at 5 m/s for 5 min. The lysate was then spun down at max speed for 5 min at 4 °C. The supernatant was collected for analysis.

Dose-dependent dehydration reaction of lysates and proteomics analysis.

Prepared lysates were resuspended in 400 µL of 4:1 NaP buffer (10 mM, pH 7.2):CH_3CN and treated with freshly prepared acetonitrile solution of $Pd(OAc)_2$ (100 µM). The reaction was stirred at room temperature for 2 h. The proteins were acetone precipitated, followed by digestion using SMART Digest™ Trypsin Kit by Thermo Scientific.

LC-MS/MS. Digested samples were resuspended in Buffer A (0.1% FA in water) and the peptide amount was determined by Pierce™ Quantitative Peptide Assays & Standards (Thermo Fisher

Scientific) according to manufactures instructions. Samples were injected into a nanoElute UPLC autosampler (Bruker Daltonics) coupled to a tims TOF Pro2 mass-spectrometer (Bruker Daltonics). The peptides were loaded on a 25 cm Aurora ultimate CSI C18 column (IonOpticks) and chromatographic separation was achieved using a linear gradient starting with a flow rate of 250 nL/min from 2% Buffer B (0.1% FA in ACN) and increasing to 13% in 42 min, followed by an increase to 23% B in 65 min, 30% B in 70 min, then the flow rate was increased to 300 nL/min and 80% B in 85 min, this was kept for 5 min. The mass-spectrometer operated in positive polarity for data collection using a data-dependent acquisition (ddaPASEF) mode. The cycle time was 1.17 s and consisted of one full scan followed by 10 PASEF/MS/MS scans. Precursors with intensity of over 2500 (arbitrary units) were picked for fragmentation and precursors over the target value of 20,000 were dynamically excluded for 1 min. Precursors below 700 Da were isolated with a 2 Th window and ones above with 3 Th. All spectra were acquired within an m/z range of 100 to 1700 and fragmentation energy was set to 20 eV at 0.6 1/K0 and 59 eV at 1.60 1/K0.

Database search (MSFragger). MS raw files were searched FragPipe GUI version 20 with MSFragger (version 3.8) as the search algorithm. Protein identification was performed with the human Swisspot database (20,456 entries) with acetylation (N-terminus), and oxidation on methionine was set variable modification. To account for the mass shift introduced by the different chemical handles a variable mass shift of 18.0106 Da on Asparagine and Glutamine with a maximal occurrence of 3 respectively. Carbamidomethylation of cysteine residues was considered a fixed modification. Trypsin was set as the enzyme with up to two missed cleavages. The peptide length was set to 7–50, and the peptide mass range of 500–5000 Da. For MS2-based experiments, the precursor tolerance was set to 20 ppm and fragment tolerance to 20 ppm. Peptide spectrum matches (PSMs) were adjusted to a 1% false discovery rate using Percolator as part of the Philosopher toolkit (v5). For label-free quantification, match-between-runs were enabled. All downstream analysis was performed in R (version 2023.03.0). Individual samples were normalized to the mean of all quantified peptides.

Data S5: Excel sheet of analysis and raw data files are attached as a supplementary document (excel-glycosylation-pathogen-analysis)

Summary of results:

Unique peptides: Excel list containing all quantified peptides were imported into RStudio and data frame of quantification columns (Intensity, Max LFQ intensity, matchtype) for each concentration extracted. Peptides in each deamidation time point (0, 30, 60, and 120 min) were sorted and filtered based on intensity (>0) and Match type of "MSMS". Duplicate entries were removed to obtain unique peptides for each concentration. List of peptides are found in:

Excel-glycosylation-pathogen-analysis (sheet 1, Yeast both reps and no_duplicate)

Excel-glycosylation-pathogen-analysis (sheet 2, Candida albican both reps and no_duplicate)

Excel – site localization (sheet 5): Note, this was run with MSFragger v4.4 leading to minor difference in found peptides compared to sheets 1-2.

Figure S10a: HeatMap analysis of N-glycosylation Ratio: To evaluate the extent of modification of each nitrile modified peptide in yeast and hyphae forms of *C. albican*, we compared the intensity ratios of each modified peptide to its unmodified counterpart. Results show an increased level of modification for yeast form, suggesting an increased N-glycosylation profile in hyphae form.

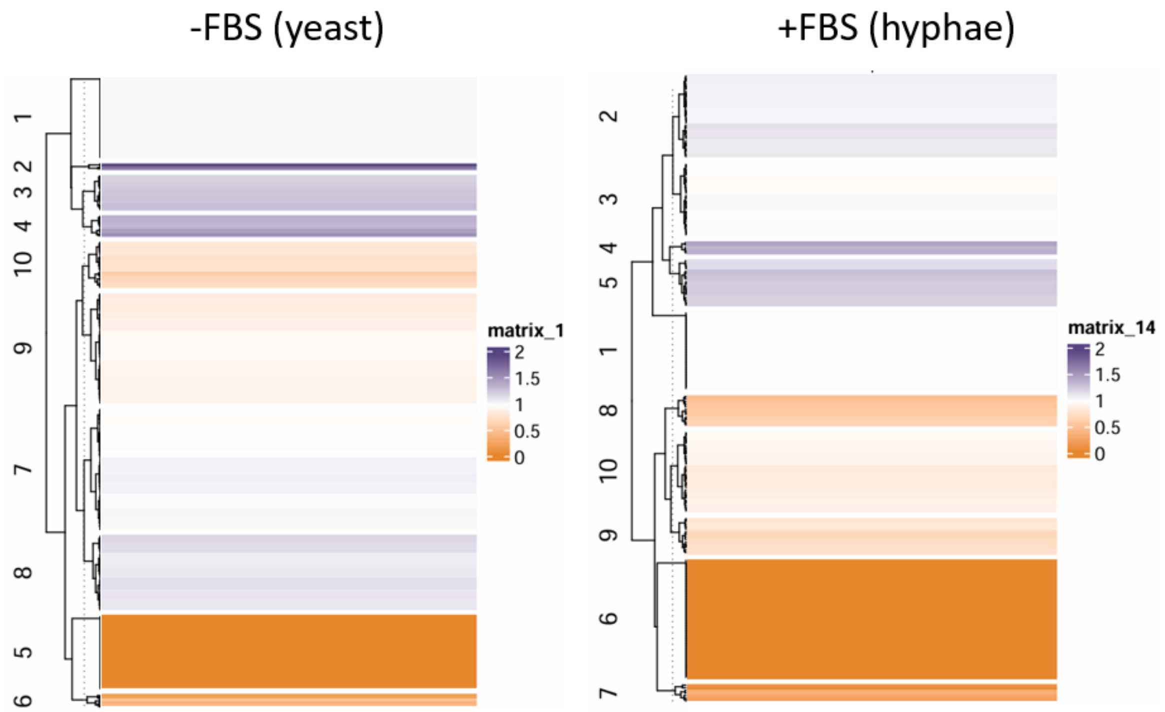

Figure S10b: Gene Ontology (GO) Analysis: To evaluate the Biological process (BP), Molecular Function (MF), and Cellular Compartment (CC) of modified proteins, Excel list containing glycosylated Asn and Gln sites were utilized. Gene ID of protein targets were extracted followed by GO analysis using ShinyGO 0.77.[3] FDR cut-off was set at 0.05%.

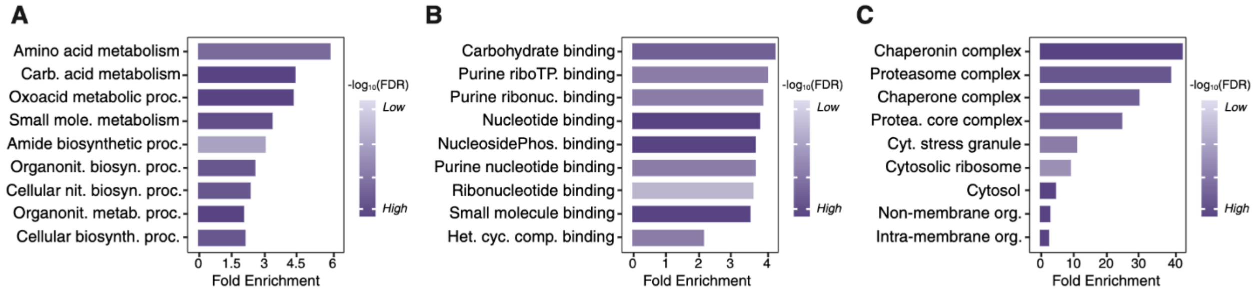

Dehydration reaction for identification of changes in N-glycosylation pattern in pathogenesis.

(A) GO-biological process analysis of proteins with upregulated N-glycosylation in *C. albicans.* **(B)** GO-molecular function analysis of proteins with upregulated N-glycosylation in *C. albicans.* **(C)** GO-cellular compartment analysis of proteins with upregulated N-glycosylation in *C. albicans.* Gene list of glycoproteins was utilized as input in ShinyGO, with input and analysis species set to *C. albicans.* All genes in the *C. albicans* genome were used as the enrichment background. Terms with a p-value < 0.01, a minimum count of 3, and an enrichment factor > 1.5 were utilized. p-values were calculated based on the cumulative hypergeometric distribution, and q-values are calculated using the Benjamini-Hochberg procedure.

We sought to evaluate the functions of upregulated N-glycosylated proteins in pathogenic *C. albicans* (nitrile modified sites observed in yeast form of *C. albicans* but not in the hyphae form). Gene ontology (GO) analysis in N-glycosylated upregulated proteins (A-C) highlights several enriched biological processes, molecular functions, and cellular components that potentially contribute to the increased pathogenic properties of the hyphae form of *C. albicans* compared to non-pathogenic yeast form. Biological processes analysis (A) showed a significant enrichment of pathways involved in amino acid metabolism, carboxylic acid metabolism, and biosynthetic processes, suggesting an enhanced metabolic flexibility in pathogenic *C. albicans* that could support growth and survival in host environment. Molecular function analysis (B) further revealed a strong enrichment in carbohydrate binding and purine ribonucleotide binding activities, which may facilitate adherence to host tissues and immune evasion, key attributes of pathogenicity.[7] Additionally, the cellular component analysis (C) shows a notable enrichment in proteins associated with the chaperonin complex, proteasome complex, and cytosolic ribosome, indicating a robust protein folding and degradation system that facilitate pathogen management of stress conditions encountered during host infection. The presence of stress granules further supports a well-adapted stress response mechanism.[8] Together, these findings suggest that pathogenic *C. albicans* possesses enhanced metabolic adaptability, stress response, and interaction capabilities, which likely contribute to its pathogenicity relative to non-pathogenic *C. albicans*, thus underlining the utility of the dehydration strategy for profiling of N-glycosylation profile of pathogenic and non-pathogenic organisms.

References

1. Chan, W.; White, P. Fmoc Solid Phase Peptide Synthesis: A Practical Approach. Oxford Universty Press, 1999).
2. O'Shea, J. P.; Chou, M. F.; Quader, S. A.; Ryan, J. K.; Church, G. M.; Schwartz, D. pLogo: a probabilistic approach to visualizing sequence motifs. *Nat. Methods* **2013**, *10*, 1211-1212.
3. Ge, S. X.; Jung, D.; Yao, R. ShinyGO: a graphical gene-set enrichment tool for animals and plants. *Bioinformatics* **2020**, *36*, 2628-2629.
4. Guha, P.; Kaptan, E.; Gade, P.; Kalvakolanu, D. V.; Ahmed, H. Tunicamycin induced endoplasmic reticulum stress promotes apoptosis of prostate cancer cells by activating mTORC1. *Oncotarget* **2017**, *8*, 68191-68207.
5. Read, A. & Schröder, M. The Unfolded Protein Response: An Overview. *Biology (Basel)* **2021**, *10*, 384.
6. Lin, B.; Qing, X.; Liao, J.; Zhuo, K. Role of protein glycosylation in host-pathogen interaction. *Cells* **2020**, *9*, 1022.
7. Protter, D. S. W.; Parker, R. Principles and properties of stress granules. *Trends Cell Biol.* **2016**, *26*, 668-679.
